# Sensitive universal detection of blood parasites by selective pathogen-DNA enrichment and deep amplicon sequencing

**DOI:** 10.1186/s40168-020-00939-1

**Published:** 2021-01-02

**Authors:** Briana R. Flaherty, Joel Barratt, Meredith Lane, Eldin Talundzic, Richard S. Bradbury

**Affiliations:** 1 Parasitic Diseases Branch, Division of Parasitic Diseases and Malaria, Center for Global Health, Centers for Disease Control and Prevention, Atlanta, GA USA; 2 Oak Ridge Institute for Science and Education, Oak Ridge, TN USA; 3 Synergy America Inc., Duluth, GA USA; 4 Malaria Branch, Division of Parasitic Diseases and Malaria, Center for Global Health, Centers for Disease Control and Prevention, Atlanta, GA USA; 5 School of Health and Life Sciences, Federation University, Ballarat, Australia

**Keywords:** Molecular parasitology, Amplicon sequencing, Blood microbiota, Parasite biodiversity, Parasite detection, Molecular diagnosis

## Abstract

**Background:**

Targeted amplicon deep sequencing (TADS) has enabled characterization of diverse bacterial communities, yet the application of TADS to communities of parasites has been relatively slow to advance. The greatest obstacle to this has been the genetic diversity of parasitic agents, which include helminths, protozoa, arthropods, and some acanthocephalans. Meanwhile, universal amplification of conserved loci from all parasites without amplifying host DNA has proven challenging. Pan-eukaryotic PCRs preferentially amplify the more abundant host DNA, obscuring parasite-derived reads following TADS. Flaherty et al. (2018) described a pan-parasitic TADS method involving amplification of eukaryotic 18S rDNA regions possessing restriction sites only in vertebrates. Using this method, host DNA in total DNA extracts could be selectively digested prior to PCR using restriction enzymes, thereby increasing the number of parasite-derived reads obtained following NGS. This approach showed promise though was only as sensitive as conventional PCR.

**Results:**

Here, we expand on this work by designing a second set of pan-eukaryotic primers flanking the priming sites already described, enabling nested PCR amplification of the established 18S rDNA target. This nested approach facilitated introduction of a second restriction digestion between the first and second PCR, reducing the proportional mass of amplifiable host-derived DNA while increasing the number of PCR amplification cycles. We applied this method to blood specimens containing *Babesia*, *Plasmodium*, various kinetoplastids, and filarial nematodes and confirmed its limit of detection (LOD) to be approximately 10-fold lower than previously described, falling within the range of most qPCR methods.

**Conclusions:**

The assay detects and differentiates the major malaria parasites of humans, along with several other clinically important blood parasites. This represents an important step towards a TADS-based universal parasite diagnostic (UPDx) test with a sufficient LOD for routine applications.

Video Abstract

**Supplementary Information:**

The online version contains supplementary material available at 10.1186/s40168-020-00939-1.

## Background

The versatility of targeted amplicon deep sequencing (TADS) enables detection of new pathogens as well as known pathogens that are difficult-to-culture and has facilitated the detailed characterization of bacterial, viral, and fungal communities from diverse biological and environmental specimens. These technologies are often used to characterize communities of microbial pathogens in human clinical samples and have been applied to the detection of antimicrobial resistance as part of routine surveillance networks [1–9]. However, the application of TADS and other next-generation sequencing (NGS)-based technologies, such as shotgun metagenomics, to the characterization of eukaryotic communities comprised of protozoa and helminths has been slow to advance, and the potential use of these technologies for parasite detection in routine diagnostic settings has not been rigorously explored.

Due to its non-specific nature, shotgun metagenomic approaches may identify unknown and/or unanticipated infectious agents from complex clinical specimens wherein few clues are provided as to the etiological agent of disease. In these circumstances, without metagenomic data, clinicians are limited in their ability to recommend the most appropriate tests for a differential diagnosis, making patient management challenging. Shotgun metagenomic sequencing of cerebrospinal fluid (CSF) has recently been applied to such complex clinical cases with great success, confirming multiple protozoal and helminthic infections, including four cases of *Balamuthia mandrillaris*-induced granulocytic amoebic encephalitis [10–12], one of *Taenia solium* neurocysticercosis [6], and four cases of meningitis caused by *Angiostrongylus cantonensis* [7, 13]. Only moderate success has been reported when applying metagenomic testing of plasma for residual pathogen DNA, even for viral and bacterial pathogens [14–16]. In a recent study of febrile illness in Uganda, this approach detected a large number of malaria infections not identified by microscopy; however, it also failed to detect some microscopy-confirmed infections [17]. Serum metagenomics has not yet been applied to rarer parasitic infections of the blood, such as trypanosomiasis, babesiosis, and leishmaniasis, or for the detection of helminthic microfilariae.

The success of metagenomic sequencing in detecting pathogens in CSF is potentially owed to the low complexity of CSF in terms of its limited extraneous DNA content compared to other biological matrices, such as tissue and stool. Accordingly, in cases of infectious meningitis or encephalitis involving host white cell inclusions in CSF greater than 200 cell/mm^3^, the sensitivity of CSF analysis is reduced by interference from background sequencing reads derived from host DNA [7]. Development of parasite-specific diagnostic primer sets is an obvious solution for overcoming the impact of host DNA interference, but this has been hampered by the conserved nature of eukaryotic housekeeping loci (e.g., genes encoding the rRNA subunits), causing pan-parasitic primers to co-amplify host DNA and obscuring parasitic infections (Flaherty 2018). This mechanism of interference has also hampered the application of direct metagenomic sequencing of parasite DNA from specimen matrices containing high numbers of nucleated cell types, such as blood and tissue [18]. Using primers not broadly specific for all eukaryotes, protozoan infections have been detected incidentally via TADS. For example, following amplification of a fragment of 28S rDNA using primers designed to be broadly specific for fungi, Gomez et al. [19] identified two *Toxoplasma gondii* infections and one *Trypanosoma cruzi* infection from brain tissue, as well as one *Leishmania* spp. infection from a skin biopsy [19]. That assay was described as having “partial protozoal coverage” although it was not designed to detect parasites specifically [19].

Targeted amplicon deep sequencing of loci amplified using pathogen-specific primers has facilitated successful detection and identification of parasitic infections in blood and feces, both from human and animal samples [20–26]. These assays have generally been restricted to a single genus or genetically similar group, such as trypanosomes, rhabditid and strongylid helminths, or protozoa [22–25]. While this strategy effectively mitigates the impact of interfering host DNA on the sensitivity of TADS-based diagnostics, it still requires prescient knowledge of the parasite the diagnostician expects to detect. Ultimately, when utilizing genus or species-specific primers, the value of TADS as pan-specific diagnostic approach is lost.

A pan-parasitic TADS method with potential diagnostic utility was recently described by Flaherty et al. [18]. That method used a broadly specific pan-eukaryotic primer pair targeting a region of the 18S rDNA gene that possessed restriction enzyme cut sites only in vertebrates. This facilitated restriction enzyme digestion of human 18S rDNA specifically, as the human 18S amplicon possessed these restriction sites that are absent in blood protozoa and filarial nematodes. This digestion step, when performed on total DNA extracts prior to PCR, significantly reduced the number of host-derived reads following TADS. This concomitantly increased the number of reads recovered from blood parasites and enabled detection of parasites in some specimens that appeared negative when undigested specimens were tested. This method detected all protozoa and helminths investigated, which included the most commonly observed parasites in human blood [18]. A drawback of this method was its limit of detection (LOD), which was comparable to most conventional PCR assays. The method was therefore less amenable to routine parasite diagnosis when compared to the more sensitive, cheaper, and less time-consuming qPCR methods already available [18]. Irrespective of this limitation, the study confirmed that taking advantage of the restriction endonuclease cut sites present in the human 18S rDNA is an effective approach for selective amplification of homologous parasite DNA; it reduced host-derived reads by more than 50%. Additionally, the number of parasite-derived reads increased by a factor of 5 to 10 times compared to their paired undigested samples using this procedure [18].

**Fig. 1:**
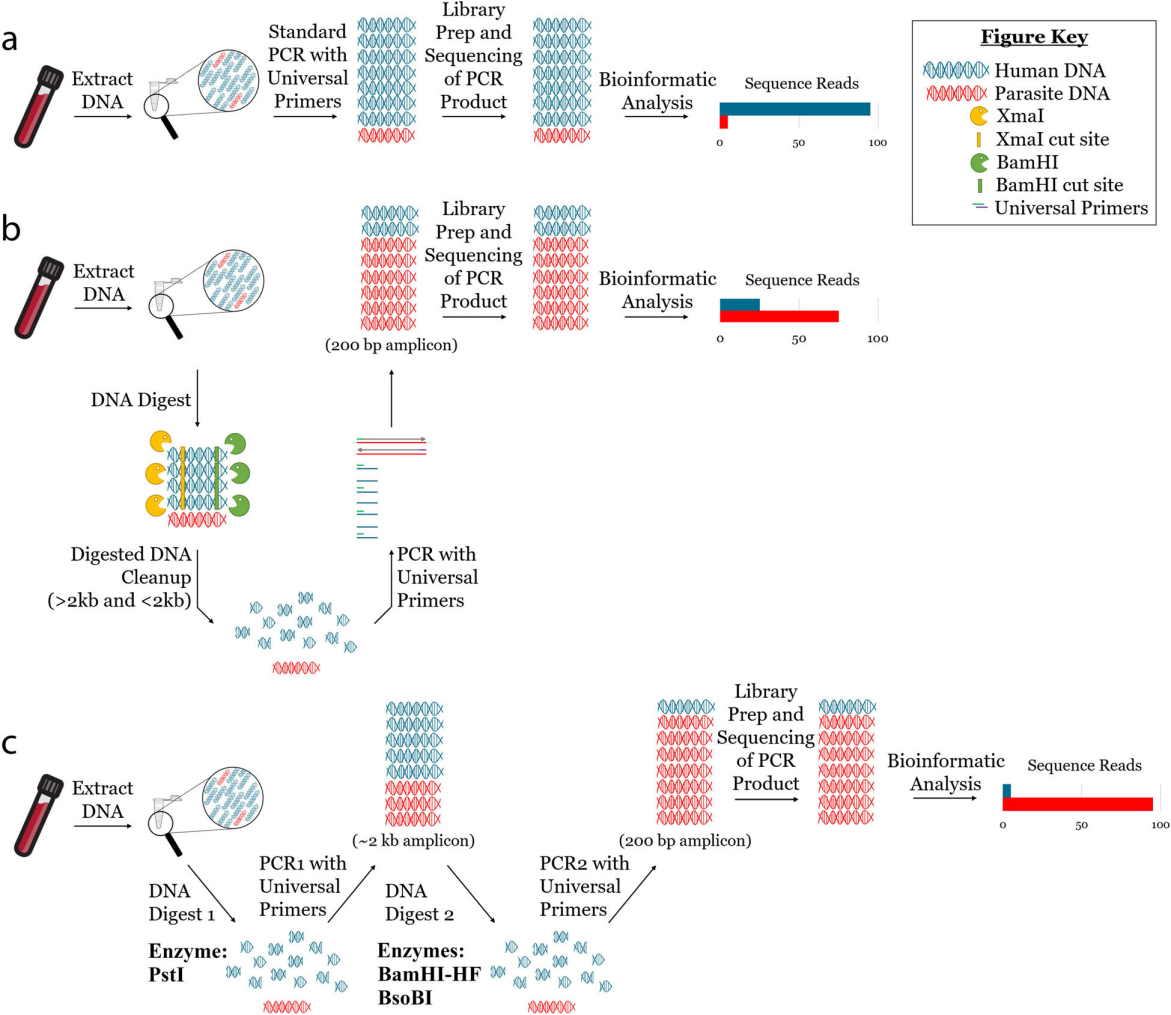
Graphical representation of our modified UPDx assay involving nested PCR amplification and two restriction digestion steps and its comparison to earlier UPDx assays. **a** Conventional PCR with universal primers amplifies primarily host DNA and yields sequencing reads almost entirely derived from the host. **b** Selective amplification of non-host eukaryote DNA via host-specific restriction enzyme digestion prior to PCR alters the ratio of amplifiable host to parasite DNA, increasing the relative mass of parasite-derived amplicon post-PCR and improving the sensitivity of parasite detection via NGS. **c** Modification of the assay described in **b** to a nested approach, facilitating an additional restriction enzyme digestion and additional amplification cycles, reduces the number of host-derived reads and enhances detection of parasite reads via NGS

In light of the promise of this method, we sought to expand on the observations of Flaherty et al. [18], towards the development of a TADS-based, pan-parasite diagnostic test for blood with a sufficient LOD to be viable for routine use. We designed a second set of pan-eukaryotic primers that flank the priming sites previously described [18], enabling nested PCR amplification of the original 18S rDNA target. This two-step nested PCR approach facilitated introduction of a secondary restriction enzyme digestion step performed between the first and second PCR, reducing the proportional mass of amplifiable host-derived DNA while increasing the number of PCR amplification cycles (Fig. 1). We applied this assay to human blood specimens confirmed positive for various parasites using routine diagnostic methods (e.g., qPCR and microscopy) including several specimens containing multiple parasite species. Our assay is approximately 10-fold more sensitive than that described by Flaherty et al. [18] and has a LOD comparable to (and exceeding) some published real-time PCRs. This marked improvement in our LOD makes our nested universal parasite diagnostic (UPDx) test more amenable for routine parasite detection in a diagnostic laboratory setting than any previously described TADS-based method for parasites.

## Methods

### Assay design and study rationale

The TADS-based method previously described by Flaherty et al. [18] utilized a pair of pan-eukaryote PCR primers targeting an ~ 200-bp region of 18S rDNA with BamHI-HF and XmaI restriction cut sites present in all vertebrates assessed but absent in all blood protozoa and helminths, to the best of our knowledge. These sites could be exploited to digest human DNA within whole DNA extracts in a targeted fashion, prior to PCR, to encourage preferential amplification of parasite DNA. Given the demonstrated utility of this locus [18], we sought to utilize the same locus but introduce several modifications that increase its amenability to routine use. To improve the assays’ LOD, we designed an additional set of pan-eukaryotic primers with priming sites flanking the original ~ 200-bp target described by Flaherty et al. [18], facilitating a nested PCR amplification of the same amplicon, which increased the number of amplification cycles.

This nested approach also introduced an opportunity for two restriction enzyme digestions to be performed: one on the total DNA extract prior to the first PCR (Digest 1: D1) and a second performed on the product of the first PCR but preceding the second PCR (Digest 2: D2). The larger ribosomal DNA region captured by the new “outer” nested primers contains a PstI restriction enzyme cut site within the human 18S rDNA target sequence, which was utilized as the restriction digestion target for D1. The second digestion is performed between the first and second PCR, taking advantage of restriction sites originally described by Flaherty et al. [18]. However, a modification of the original assay was implemented, by replacing XmaI with the BsoBI restriction enzyme during D2, noting that BsoBI also has restriction sites within our target amplicon that do not exist for the blood protozoa and filarial nematodes examined here, and confirming an additional choice for restriction enzyme digestion during D2. While it may be of no consequence for this assay, we note that BsoBI is not sensitive to CpG methylation. CpG methylation is an epigenetic mechanism utilized by all mammals for gene silencing as well as during embryonic development [27], and XmaI activity is impaired by CpG methylation.

To assess the performance of our modified nested TADS-based approach, we compare it directly to the original method described by Flaherty et al. [28] and to a qPCR used for routinely detecting *Plasmodium falciparum* in the Parasitic Diseases Branch at CDC. We apply our modified assay to an assortment of blood specimens positive for a range of parasites comprising either blood from healthy donors spiked with parasites or their DNA, or positive clinical specimens confirmed as part of the routine diagnostic activities performed in the Parasitic Diseases Branch at CDC. The precise methodologies are detailed in later sections.

### Source of samples

The majority of human clinical blood samples used in this study were originally submitted to the CDC Parasitic Diseases Branch for confirmatory diagnosis of parasitic infections. Following diagnosis, samples were de-identified and frozen in 200 μL aliquots at − 80 °C for later use. De-identification involved the collector of the specimens providing an aliquot to the authors of this study in an ambiguously marked container (e.g., “*P. falciparum* specimen 1”) so that its linkage back to the patients was not possible. Samples acquired in this manner include the following: *P. falciparum*, *Plasmodium vivax*, *Plasmodium malariae*, *Plasmodium ovale*, *Babesia microti*, *Babesia divergens*-like variant MO1, *T. cruzi* (HIV-positive and HIV-negative clinical samples), *Loa loa*, and NPF (no parasite found)—used as negative controls. A blood spot on filter paper collected in the Democratic Republic of Congo from a person with *Mansonella perstans* microfilaremia was provided by Dr. Vita Cama of the CDC Parasitic Diseases Branch. Acute *T. cruzi* infection EDTA blood DNA extracts were generously provided by Dr. Stella Chenet and Dr. Maria Isabel Jercic of the Institute of Public Health, Chile (Santiago). *B. divergens* was obtained from the infected blood of laboratory-raised gerbils, and *Babesia duncani* was obtained from the infected blood of laboratory-raised guinea pigs, routinely maintained in CDC’s animal facility (Table 1). Bioinformatic analysis confirmed that the restriction enzyme cut sites were present in all the respective animal species for all relevant animal samples utilized here. For some rare blood-borne parasites (*Plasmodium knowlesi* and *Brugia malayi*), either animal blood or human blood collected during previous studies and stored at − 80 °C were used. Rare blood parasites that could not be acquired as true clinical samples at the time of the study were recreated by spiking uninfected human blood with cultured parasites—*Leishmania infantum*, *Leishmania donovani*, *T. cruzi*, and *Trypanosoma brucei* subsp. *rhodesiense* cultures were added to whole human blood at a ratio of 1:100. All blood samples were collected into EDTA anticoagulant. Full details regarding specimen source, matrix, original parasite identification method, and DNA extraction method are provided in Table 1. Simulated mixed parasite infections were prepared according to the descriptions in Table S1, which can be found in Supplementary File S1 on page 4.

**Table 1:** Host, source, and original identification method of samples used in this study

Parasite	Specimen number	Sample type	Host	Species identification diagnostic method/s
*Plasmodium falciparum*	Specimen 1	EDTA blood	*Homo sapiens*	Microscopy and PCR w/s [29]
*Plasmodium vivax*	Specimen 2	EDTA blood	*Homo sapiens*	Microscopy and PCR w/s [29]
*Plasmodium ovale*	Specimen 3	EDTA blood	*Homo sapiens*	Microscopy and PCR w/s [29]
*Plasmodium malariae*	Specimen 4	EDTA blood	*Homo sapiens*	Microscopy and PCR w/s [29]
*Plasmodium knowlesi*	Specimen 5	EDTA blood	*Macaca mulatta*	Microscopy
*Babesia microti*	Specimen 6	EDTA blood	*Homo sapiens*	Microscopy and PCR w/s [30]
*Babesia divergens*	Specimen 7	EDTA blood	*Meriones unguiculatus*	Microscopy and PCR w/s [30]
*Babesia duncani*	Specimen 8	EDTA blood	*Meriones unguiculatus*	Microscopy and PCR w/s [30]
*Babesia divergens*-like variant MO1	Specimen 9	EDTA blood	*Homo sapiens*	Microscopy and PCR w/s [30]
*Trypanosoma cruzi* (infections)	Specimens 10 and 11	EDTA blood	*Homo sapiens*	Real-time PCR (Qvarnstrom 2012)
*Trypanosoma cruzi* (culture)	Specimen 12	RPMI culture in EDTA blood	*Homo sapiens*	Microscopy
*Trypanosoma brucei*	Specimen 13	HMI-9 culture in EDTA blood	*Homo sapiens*	Microscopy
*Leishmania infantum*	Specimen 14	RPMI culture in EDTA blood	*Homo sapiens*	Microscopy and PCR w/s [31]
*Leishmania donovani*	Specimen 15	RPMI culture in EDTA blood	*Homo sapiens*	Microscopy and PCR w/s [31]
*Brugia malayi*	Specimen 16	EDTA blood	*Felis catus*	Microscopy
*Loa loa*	Specimen 17	EDTA blood	*Homo sapiens*	Real-time PCR [32]
*Mansonella perstans*	Specimen 18	Blood spot on filter paper	*Homo sapiens*	Real-time PCR [33, 34]
NPF (no parasite found)	N/A	EDTA blood	*Homo sapiens*	Microscopy, PCR [29–31]

*w/s* with Sanger sequencing of PCR product, *CDC* Centers for Disease Control and Prevention, *FR3* Filariasis Research Reagent Resource Center, *PDB* Parasitic Diseases Branch, *UGA* University of Georgia, *NIH* National Institutes of Health

### 3D7 parasite culture

*Plasmodium falciparum* 3D7 parasites were grown according to routine methods [28]. Parasites were cultured in human O^+^ red blood cells at 4% hematocrit under a gas mixture of 90% nitrogen, 5% oxygen, and 5% CO_2_. Cultures were maintained in 25 or 75 cm^2^ tissue culture flasks at 37 °C and in complete culture medium made up of RPMI containing 25 mM HEPES, 0.05 mg/mL hypoxanthine, 2.2 mg/mL NaHCO_3_, 0.5% inactivated O^+^ human serum, 2 g/L glucose, and 0.01 mg/mL gentamicin. Culture growth was assessed daily via Giemsa stain and passaged or supplemented as necessary.

### Primer design

To design pan-eukaryote “outer” nested primers, Geneious software (Biomatters Inc, Newark, NJ, USA) was used to align the 18S rRNA genes from publicly available sequences of 29 species of protozoa, 30 species of helminths, and *Homo sapiens*. A list of these sequences is provided in Supplementary File S1, Appendix A. The PCR1 (outer) primer sequences are as follows: TTGATCCTGCCAGTAGTCATATGC (outer forward) and GGTGTGTACAAAGGGCAGG GAC (outer reverse), generating a PCR product of approximately 2 kb. Inner primers for the universal nested PCR were identical to those previously described by Flaherty et al. [18], with primer sequences as follows: CCGGAGAGGGAGCCTGAGA (inner forward) and GAGCTGGAATTACCGCGG (inner reverse), generating a PCR product of approximately 200 bp. Primers were synthesized at the CDC Biotechnology Core Facility.

### Universal Blood Parasite Detection

DNA from 200 μL of parasite-free or parasite-infected whole blood was extracted using a QIAamp DNA Blood Mini QIACube Kit (Qiagen) and a Qiagen QIACube for automated sample preparation. Samples and negative extraction controls were processed according to the kit protocol and eluted into 50 μL of Qiagen Buffer EB. Following extraction, 8.5 μL of DNA extract was digested for 2 h at 37 °C with 10 units of PstI (0.50 μL) and 1 μL of 10X CutSmart Buffer (digest D1). Digested DNA (2 μL of that digest) was subsequently amplified (PCR1) in a volume of 25 μL per reaction. For PCR1 cycling, samples were denatured at 98.0 °C for 30 s followed by 15 cycles of 98.0 °C for 10 s, primer annealing at 67.0 °C for 30 s and extension at 72.0 °C for 2 min, and a final extension at 72.0 °C for 2 min. Thereafter, the entire 25 μL product of PCR1 was again digested for 1 h at 37 °C by directly adding 10 units of BamHI-HF (0.5 μL), 10 units of BsoBI (1 μL), and 2.5 μL of 10X CutSmart Buffer (digest D2) to the original PCR tube. Two microliters of that digested product were then transferred into PCR2 (20 μL per reaction). All restriction enzymes were purchased from NEB (Ipswich, MA, USA). For PCR2, samples were denatured at 98.0 °C for 30 s followed by 30 cycles of 98.0 °C for 10 s, primer annealing at 67.0 °C for 30 s and extension at 72.0 °C for 45 s, and a final extension at 72.0 °C for 2 min. All PCR reactions (both PCR1 and PCR2) contained Q5 Buffer, dNTPs, forward and reverse primers (1.25 μL of 10 μM stock), Q5 High GC Enhancer, and Q5 High-Fidelity DNA Polymerase (NEB, Ipswich, MA, USA), in the concentrations specified in the manufacturer’s instructions. Only the reaction volumes varied between PCR1 (25 μL) and PCR2 (20 μL). Each PCR run was accompanied by at least three negative controls—DNA extracts of parasite-negative blood. To establish the conditions described here, assay parameters were optimized systematically using DNA from cultures of *Leishmania infantum* spiked into human blood and *P. knowlesi*-containing *Macaca mulatta* blood; optimal conditions were considered to be those that most reduced the number of vertebrate-derived reads in the final sequence dataset while maximizing the number of parasite-derived reads. An overview of these optimization experiments is provided in Supplementary File S1. Specimens were processed using the method described here in addition to comparing different restriction digestion procedures: specimens prepared using only Digest 1, only Digest 2, or both digestion steps. Each of these three reaction conditions was applied to the same specimens in triplicate and sequenced on three separate MiSeq sequencing runs. These results were compared to a single replicate prepared for each specimen analyzed using the method described by Flaherty et al. [18]. The significance of differences between all conditions were assessed using a 2-way ANOVA with Tukey’s multiple comparisons posttest.

### Amplicon sequencing

Following PCR2 samples were analyzed on a 1.5% agarose gel, cleaned with the Monarch PCR & DNA Cleanup Kit (< 2 kb), and eluted with 20 μL elution buffer, and final amplicon concentration was determined using a Qubit 2.0 Fluorometer with the Qubit dsDNA High Sensitivity Assay Kit (Life Technologies, Grand Island, NY, USA). These final concentrations were used downstream to normalize the DNA during library preparation. Samples were then diluted 1:5 in NEB Elution Buffer and transferred to the CDC Biotechnology Core Facility’s Genome Sequencing Lab for library preparation and DNA amplicon sequencing using the NEBNext Ultra DNA Library Prep Kit for Illumina (NEB), the MiSeq Reagent Nano Kit v2 (Illumina), and sequencing on an Illumina MiSeq Sequencing platform (Illumina). Alternatively, the same library preparation and sequencing procedure as previously described was performed within the Parasitic Diseases Branch at CDC and sequenced on the CDC Division of Parasitic Diseases and Malaria Sequencing Facility’s Illumina MiSeq. Each specimen was analyzed as a single replicate using this workflow, except for the specimens included in the limit of detection experiments (described below) which were performed in triplicate. All raw reads have been made publicly available on the NCBI Sequence Read Archive under BioProject accession number PRJNA437674. Specimens relevant to the present study under this BioProject include the text “nUPDx method validation” in their title.

### Bioinformatic analysis

Analysis of sequence data was undertaken using a custom workflow designed in Geneious (Geneious Prime, version 11: www.geneious.com). This workflow first removed primer sequence from either end of the Illumina reads (250 bases, paired end) using the Trim Ends plugin allowing 3 mismatches and a minimum match length of 5 bases. Illumina adapter trimming was next performed using BBDuk. Low-quality ends were trimmed at either end (minimum Phred score of 20), and reads shorter than 50 bases in length were discarded. Paired reads were then merged using default parameters. Next, a BLASTN search was performed against a database constructed from human 18S rDNA sequences and some human 18S pseudogenes (GenBank Accession numbers: X03205.1, XR_003508809.1, NG_055289.1, NG_054751.1, AC129664.7) with the percent identity set to 99%, a word size of 11, and the qcov_hsp_perc flag set to 60. Reads obtaining BLASTN hits to this database using these parameters were discarded. The remaining reads were assembled using the Geneious de novo assembler applying the following custom parameters: a minimum overlap of 50 bases and a minimum overlap identity of 100%. The resulting haplotypes were next subjected to a BLASTN search (default parameters) against a reference database containing a set of 18S sequences compiled from a range of parasites (GenBank Accession numbers are provided in Tables 2 and 3), and the BLASTN hit/s obtaining the nearest match to the haplotype/s detected were exported to text. Finally, the proportion of merged reads used to construct each haplotype was calculated as a percentage of the total number of merged reads generated for that specimen. To distinguish between positive and negative results, we employed the same cutoff system described by Flaherty et al. [18], where the number of merged reads used to construct a parasite-derived haplotype was calculated as a proportion of the total number of merged reads generated for that specimen. This was used to determine the specimens’ status as positive or negative, based on the number of parasite-derived reads detected in parasite-negative blood specimens, as per Flaherty et al. [18]. To investigate the ability of the sequenced amplicons to differentiate between various parasite taxa, a cluster dendrogram was generated from the haplotypes generated from various specimens. Haplotypes were aligned in Geneious and exported as a fasta file. The alignment was imported into R, and a distance matrix was generated including gaps in the distance measurement, using the “seqinr” R package. This matrix was clustered using hierarchical agglomerative nesting (AGNES) in the R package “cluster,” version 2.0.6. AGNES was performed using Ward’s clustering method with all other parameters set to default. The resulting tree was visualized using the R package “ggtree.”

**Table 2:** UPDx (double digest) results obtained for clinical validation specimens confirmed positive for parasites by other diagnostic methods

Parasite in sample (specimen #)	Reads assembled	Total reads	% Reads assembled	% Similarity to sequence	GB accession
***Plasmodium falciparum*** **(Specimen 1)**	7457	15,861	47.01	100.0% similar to *Plasmodium falciparum*	XR_002966654.1^b^
	4820	15,861	30.39	100.0% similar to *Plasmodium falciparum*	XR_002273081.2
	239	15,861	1.51	99.4% similar to *Plasmodium falciparum*	XR_002966654.1 ^b^
***Plasmodium vivax*** **(Specimen 2)**	6584	11,546	57.02	100.0% similar to *Plasmodium vivax*	XR_003001206.1
	2820	11,546	24.42	100.0% similar to *Plasmodium vivax*	LT635616.1
	208	11,546	1.80	100.0% similar to *Plasmodium vivax*	U93234.1
***Plasmodium ovale*** **(Specimen 3)**	11,617	15,295	76.00	100.0% similar to *Plasmodium ovale wallikeri*	MG255222.1
	122	15,295	0.798	100.0% similar to *Plasmodium ovale wallikeri*	KY073344.1
***Plasmodium malariae*** **(Specimen 4)**	8754	12,200	71.75	100.0% similar to *Plasmodium malariae*	LT594631.1
***Plasmodium knowlesi*** **(Specimen 5)**	10,119	11,999	84.33	100.0% similar to *Plasmodium knowlesi*	MF370109.1
***Babesia microti*** **(Specimen 6)**	9554	10,770	88.71	100.0% similar to *Babesia microti*	LC314658.1
***Babesia divergens*** **(Specimen 7)**	11,847	13,546	87.5	100.0% similar to *Babesia* sp.	MG944238.1
***Babesia duncani*** **(Specimen 8)**	11,011	12,591	87.5	100.0% similar to *Babesia duncani*	HQ289870.1
***Babesia divergens-*like variant MO1 (Specimen 9)**	10,842	12,323	88.0	100.0% similar to *Babesia* sp.	MG944238.1
***Trypanosoma cruzi*** **(clinical–Specimen 10)**	1882	18,229	10.32	100.0% similar to *Demodex* sp.^a^	MH891494.1
	111	18,229	0.609	99.4% similar to *Demodex* sp.^a^	MH891494.1
***Trypanosoma cruzi*** **(HIV+ clinical–Specimen 11)**	1824	14,897	12.24	100.0% similar to *Trypanosoma cruzi*	AF288661.1
	158	14,897	1.06	100.0% similar to *Trypanosoma cruzi*	CP015675.1
***Trypanosoma cruzi*** **(culture–Specimen 12)**	8852	15,471	57.2	100.0% similar to *Trypanosoma cruzi*	CP015675.1
	182	15,471	1.18	99.4% similar to *Trypanosoma cruzi*	CP015675.1
***Trypanosoma brucei*** **(Specimen 13)**	7928	18,334	43.24	100.0% similar to *Trypanosoma brucei brucei*	XR_002989632.1
	1361	18,334	7.42	99.4% similar to *Trypanosoma brucei brucei*	XR_002989632.1
***Leishmania infantum*** **(Specimen 14)**	11,735	17,418	67.373	100.0% similar to *Leishmania* sp.	GQ332359.1
***Leishmania donovani*** **(Specimen 15)**	11,781	14,653	80.400	100.0% similar to *Leishmania* sp.	GQ332359.1
	110	14,653	0.751	99.4% similar to *Leishmania* sp.	GQ332359.1
***Brugia malayi*** **(Specimen 16)**	1042	13,117	7.944	100.0% similar to *Dirofilaria repens*	MG780293.1
***Loa loa*** **(Specimen 17)**	2003	17,439	11.49	100.0% similar to *Wuchereria bancrofti*	AY843436.1
***Mansonella perstans*** **(Sample 18)**	180	15,196	1.18	100.0% similar to *Filarioidea* sp.	KT907503.1

Note: A 100% match to a sequence does not necessarily indicate that this is the species detected. The amplicon sequenced is sufficient for a species diagnosis for only some parasites. Please refer to Fig. 5 for details

^a^*Demodex* are mites that commonly inhabit the pores in normal human skin and are considered non-pathogenic, we suspect that this represents potential contamination with skin debris from the venipuncture site. *T. cruzi* was not detected in this specimen

^b^Examples where multiple hits to the same accession were observed. These are both shown because the percentage similarity to these accessions is different for these two haplotypes

**Table 3:** Positive detection of multiple parasites in a single specimen using UPDx

Sample name	Spiked analytes	% Similarity to reference sequences	Parasite abbreviation: accession # (% reads assembled^a^)	Total % reads assembled
**Mix 1**	***P. falciparum***	100.0% similar to *Plasmodium falciparum*	Pf: XR_002966654.1 (2.3%), XR_002273081.2 (2.1%)	4.4
	***P. vivax***	100.0% similar to *Plasmodium vivax*	Pv: LT635616.1 (15.8%), XR_003001206.1 (43.9%)	59.7
**Mix 2**	***P. ovale***	100.0% similar to *Plasmodium ovale wallikeri*	Po: MG255222.1 (34.1%), KY073344.1 (1.5%)	35.6
	***P. falciparum***	100.0% similar to *Plasmodium falciparum*	Pf: XR_002966654.1 (13.9%), XR_002273081.2 (9.3%)	23.2
**Mix 3**	***P. falciparum***	100.0% similar to *Plasmodium falciparum*	Pf: XR_002966654.1 (23.1%), XR_002273081.2 (15.1%)	38.2
	***P. malariae***	100%% similar to *Plasmodium malariae*	Pm: LT594631.1 (15.9%)	15.9
**Mix 4**	***P. falciparum***	100.0% similar to *Plasmodium falciparum*	Pf: XR_002966654.1 (5.6%), XR_002273081.2 (4.5%)	10.1
	***P. knowlesi***	100.0% similar to *Plasmodium knowlesi*	Pk: MF370109.1 (55.0)	55.0
**Mix 5**	***P. falciparum***	100.0% similar to *Plasmodium falciparum*	Pf: XR_002966654.1 (1.5%), XR_002273081.2 (1.3%)	2.8
	***P. vivax***	100.0% similar to *Plasmodium vivax*	Pv: LT635616.1 (10.0%), XR_003001206.1 (27.2%), U93234.1 (0.6%)	37.8
	***P. ovale***	100.0% similar to *Plasmodium ovale wallikeri*	Po: MG255222.1 (5.4%)	5.4
	***P. malariae***	100.0% similar to *Plasmodium malariae*	Pm: LT594631.1 (1.6%)	1.6
	***P. knowlesi***	100.0% similar to *Plasmodium knowlesi*	Pk: MF370109.1 (16.8)	16.8
**Mix 6**	***P. falciparum***	100.0% similar to *Plasmodium falciparum*	Pf: XR_002966654.1 (22.4%), XR_002273081.2 (12.5%)	34.9
	***T. cruzi***	100.0% similar to *Trypanosoma cruzi*	Tc: CP015675.1 (20.5%), AF288661.1 (1.2%)	21.7
**Mix 7**	***P. vivax***	100.0% similar to *Plasmodium vivax*	Pv: LT635616.1 (17.4%), XR_003001206.1 (43.8%)	61.2
	***T. cruzi***	100.0% similar to *Trypanosoma cruzi*	Tc: CP015675.1 (2.5%)	2.5
**Mix 8**	***P. falciparum***	100.0% similar to *Plasmodium falciparum*	Pf: XR_002966654.1 (27.0%), XR_002273081.2 (15.0%)	42.0
	***T. brucei***	100.0% similar to *Trypanosoma brucei brucei*	Tb: XR_002989632.1 (10.3%)	10.3
**Mix 9**	***P. falciparum***	100.0% similar to *Plasmodium falciparum*	Pf: XR_002966654.1 (27.0%), XR_002273081.2 (15.0%)	52.2
	***L. loa***	100.0% similar to *Loa loa* and *Wuchereria bancrofti*^b^	Ll: AY843436.1 (0.91%)	0.91
**Mix 10**	***P. falciparum***	100.0% similar to *Plasmodium falciparum*	Pf: XR_002966654.1 (34.6%), XR_002273081.2 (18.4%)	49.2
	***B. malayi***	100.0% similar to filarial nematode sequences^c^	Bm: MG780293.1 (0.55%)	0.55
**Natural mixed infection**	**N/A**	100.0% similar to *Plasmodium falciparum*	Pf: XR_002966654.1 (0.80%), XR_002273081.2 (0.80%)	1.60
		100.0% similar to *Plasmodium malariae*	Pm: LT594631.1 (2.2%)	2.2

^a^Percentage of reads assembled to produce a haplotype that obtained a 100% similarity BLASTN hit to the sequence associated with the accession numbers provided

^b^The sequence generated cannot distinguish between *Loa loa* and *Wuchereria bancrofti*

^c^The sequence generated from this amplicon cannot differentiate between several filarial nematodes. For example, our reference database included a sequence from *Dirofilaria repens* though it is important to note that the nucleotide sequence is identical to *Brugia malayi* at the 18S rDNA region captured by UPDx

### Limit of detection and comparison to qPCR for *P. falciparum*

Cultured *P. falciparum* 3D7 parasites (primarily ring-stage) were washed once with RPMI and subsequently spiked into whole blood to simulate a *P. falciparum* infection. Thick and thin smears of these simulated infections were then fixed with methanol and stained with 2.5% Giemsa stain for assessment of parasitemia. Concurrently, the total number of white blood cells (WBC) in the cell preparation was determined using a Beckman Coulter Ac-T Diff Hematology Analyzer. The cell preparation, which was determined to be at a parasitemia of 58,000 parasites/μL, was serially diluted to generate *P. falciparum* samples in whole blood ranging from 58,000 to 0.0058 parasites/μL. Serial dilutions were processed in triplicate, using the method described here, comparing different restriction digestion procedures: only Digest 1, only Digest 2, or both digestion steps. Each of these reaction conditions was applied to the same specimen aliquots in triplicate and sequenced on three separate MiSeq sequencing runs. Resultant reads were analyzed bioinformatically as described above and compared to a single replicate analyzed using the method described by Flaherty et al. [18]. The significance of differences between all conditions was assessed using a 2-way ANOVA with Tukey’s multiple comparisons posttest. The impact of performing only D1, only D2, and the double digestion procedure was also assessed on specimens positive for all other parasite species examined in this study to ensure that the observed impact of the three digestion procedures on *P. falciparum* could also be generalized to other parasites (as described above). DNA extracted from these same serial dilutions of *P. falciparum* in whole blood was also tested using a routine, duplex, species-specific qPCR designed to detect and differentiate both *P. falciparum* and *P. vivax* as previously described [29]. For this assay, 5 μL of DNA extract was provided as template in a total volume of 25 μL [29]. Two *P. falciparum* clinical samples that had tested positive during routine testing at the CDC molecular diagnostic laboratory were de-identified and used as positive controls. A no-template water control was used as a negative control. Samples were run in triplicate, and an average CT for each dilution was calculated and compared with the nested PCR TADS method. While we were aware that the specimens contained only *P. falciparum*, the assay was prepared exactly as described including a FAM probe for *P. falciparum* and a CY5 probe for *P. vivax*.

## Results

### UPDx with double digestion maximizes reduction of host-derived reads

**Fig. 2:**
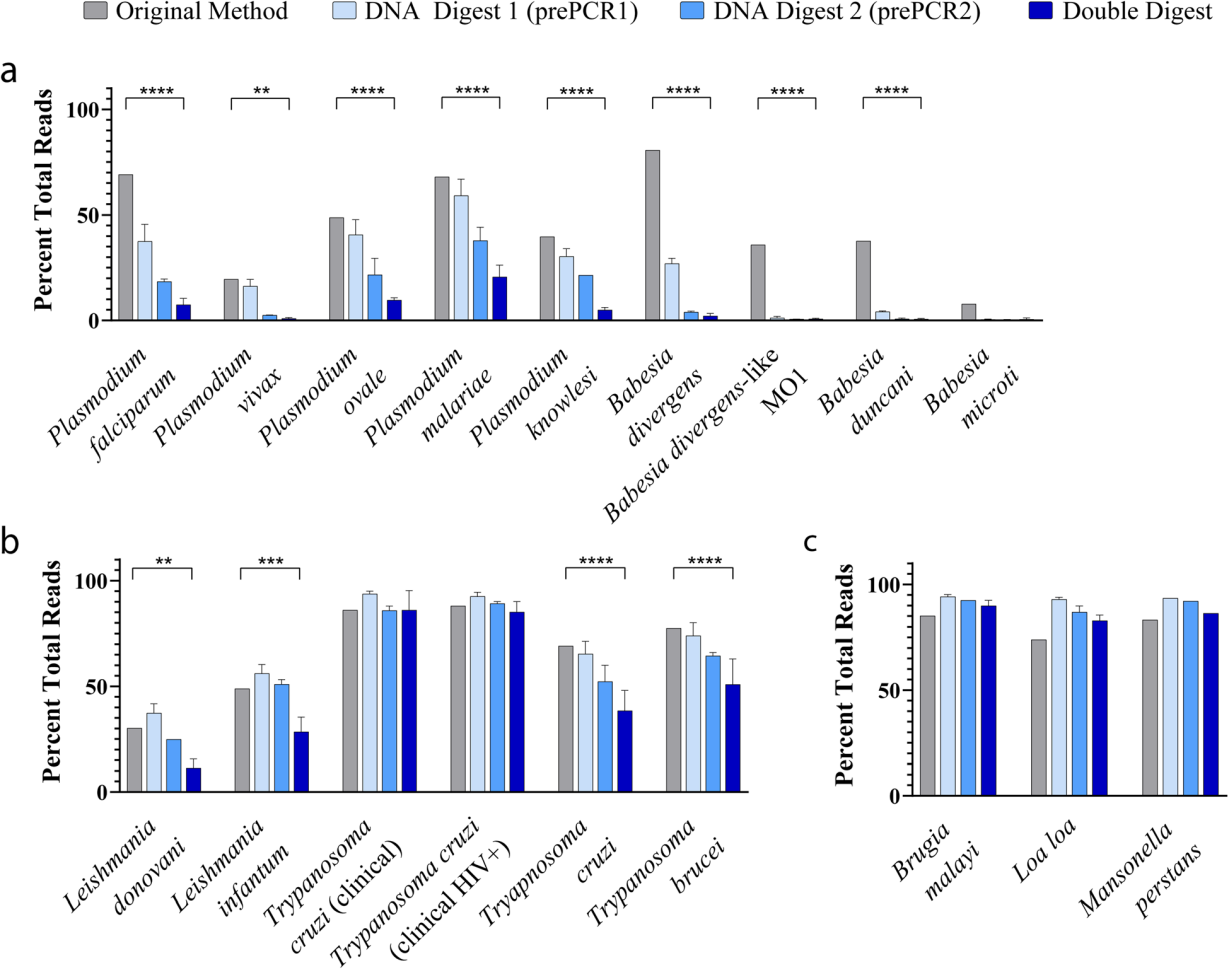
UPDx with double digestion significantly reduces the number of human-derived reads recovered following TADS. Restriction enzyme digestion yields a marked reduction in the percentage of human-derived 18S rDNA reads in parasite-infected blood samples following PCR and TADS. Human-derived read reduction is most pronounced in samples undergoing both restriction Digest 1 and Digest 2, as evidenced by the percentage of human-derived reads in most blood samples containing apixomplexan (**a**) and kinetoplastid (**b**) parasites. However, no visible reduction in human reads is observed in human blood displaying low parasitemia microfilarial infections (**c**, 2-way ANOVA with Tukey’s multiple comparisons posttest, **** *p* < 0.0001, *** *p* < 0.001, ** *p* < 0.01, *n* = 3, mean ± SD)

A substantial reduction in human-derived reads was observed in samples processed via the nested UPDx method with two restriction digests prior to TADS in comparison with the method described by Flaherty et al., which involved restriction digestion of the DNA extract only followed by a single PCR amplification (Fig. 2) [18]. Samples that underwent both Digest 1 (D1: digestion before the first PCR) and Digest 2 (D2: digestion between the first and second PCR) contained fewer host-derived reads than samples that underwent D1 or D2 alone (Fig. 2). Samples that underwent only D2 contained fewer host-derived reads than those subjected to D1 alone, and specimens subjected to D2 alone had fewer host-derived reads than samples processed by the method described by Flaherty et al. [18] (Fig. 2). We compared the earlier amplicon sequencing method [18] to our UPDx approach for 16 blood parasites (Fig. 2), including nine apicomplexans (various *Plasmodium* and *Babesia* species), four kinetoplastids (*Leishmania donovani*, *Leishmania infantum*, *Trypanosoma cruzi* and *Trypanosoma brucei*), and three filarial nematodes (*Brugia malayi*, *Loa loa*, and *Mansonella perstans*).

**Fig. 3:**
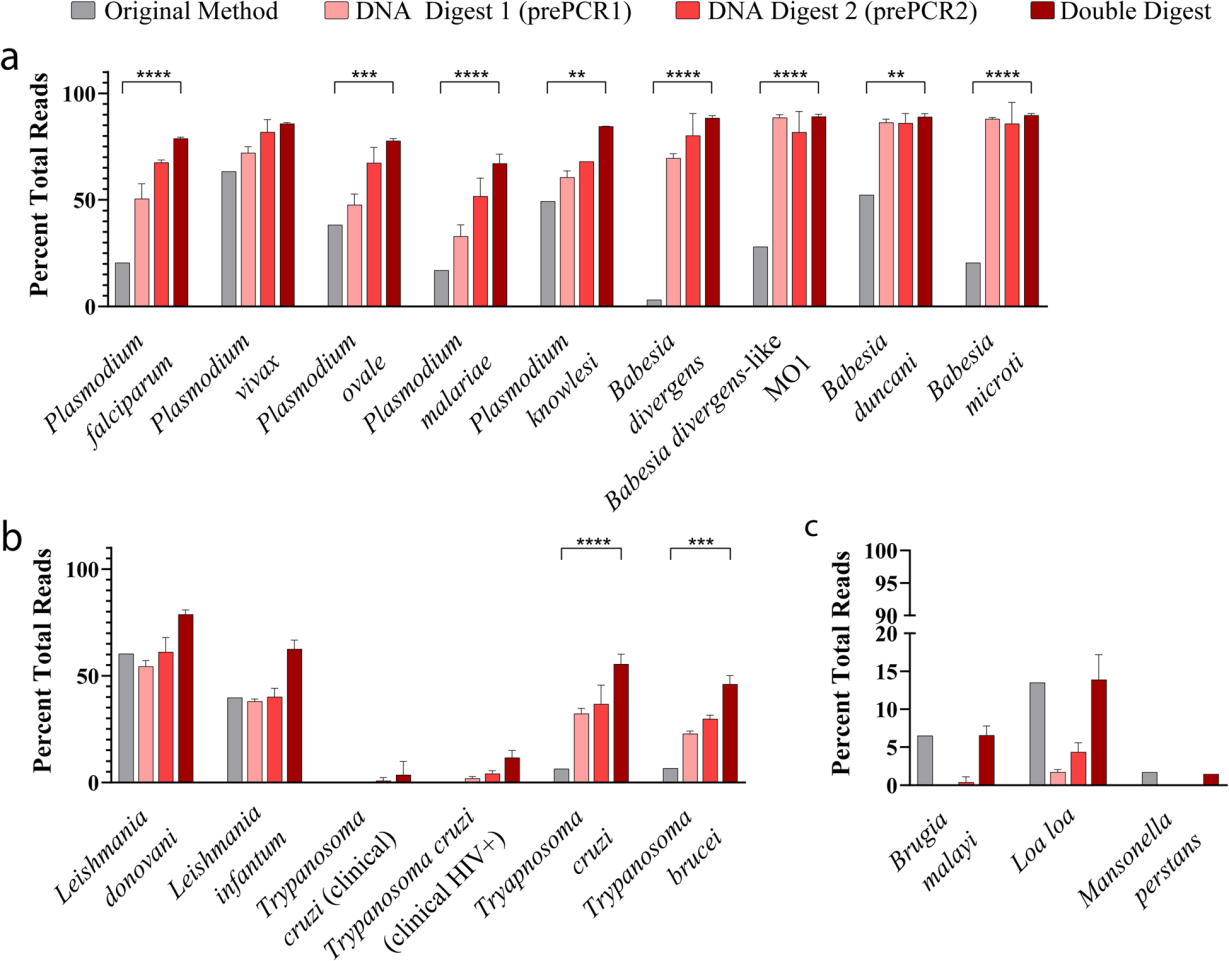
UPDx with double digestion increases the number of parasite-derived reads recovered following TADS. Restriction enzyme digestion yields a substantial increase in the percentage of parasite-derived 18S rDNA reads from parasite-infected whole blood samples following PCR and TADS. The increase in parasite reads is most pronounced in samples undergoing both restriction Digest 1 and Digest 2, as evidenced by the percentage of parasite-derived reads recovered from blood samples containing apixomplexans (**a**), kinetoplastids (**b**), and microfilariae (**c**), (statistical analysis involved a 2-way ANOVA with Tukey’s multiple comparisons posttest, **** *p* < 0.0001, *** *p* < 0.001, ** *p* < 0.01, *n* = 3, mean ± SD)

For eight of nine simulated apicomplexan blood parasite infections, we observed a statistically significant reduction (*p* < 0.01, 2-way ANOVA) in the percentage of host-derived reads detected using UPDx (~ 1% host-derived reads) compared to the original method (~ 20% host-derived reads) (Fig. 2a). For seven of these apicomplexan infections, the reduction was highly significant (*p* < 0.0001), where between ~ 30 and ~ 70% of recovered reads were derived from the host using the earlier method versus between ~ 1 and ~ 20% for UPDx (Fig. 2a). A *Babesia microti* infection did not yield a significant difference between the number of host-derived reads recovered when the specimen was tested using both methods, yet a reduction was still observed for UPDx; less than 1% of reads were host-derived using UPDx, while ~ 8% of reads were host-derived using the original method (Fig. 2a). For the kinetoplastids, a similar significant reduction in host-derived reads was observed in most cases using UPDx compared to the earlier method, except for some clinical *T. cruzi* infections, where no significant difference was observed (Fig. 2b). Similarly, for each of three infections caused by different species of filarial nematode, no significant difference was observed between our UPDx approach and the original method (Fig. 2c). Ultimately, for the majority of infections tested, the proportional reduction in host reads following nested PCR with double digestion (specimens undergoing both D1 and D2) was significantly greater than the proportional reduction in host reads described by Flaherty and colleagues using their method [18] (Fig. 2). Similarly, in the majority of cases, subjecting specimens to the nested UPDx method described here combining both D1 and D2 also significantly increased the number of parasite-derived reads detected following TADs when compared to specimens subjected to nested UPDx with D1 or D2 alone and compared to specimens subjected to the original method described by Flaherty et al. [18] (Fig. 3).

### UPDx has a limit of detection comparable to qPCR

**Fig. 4:**
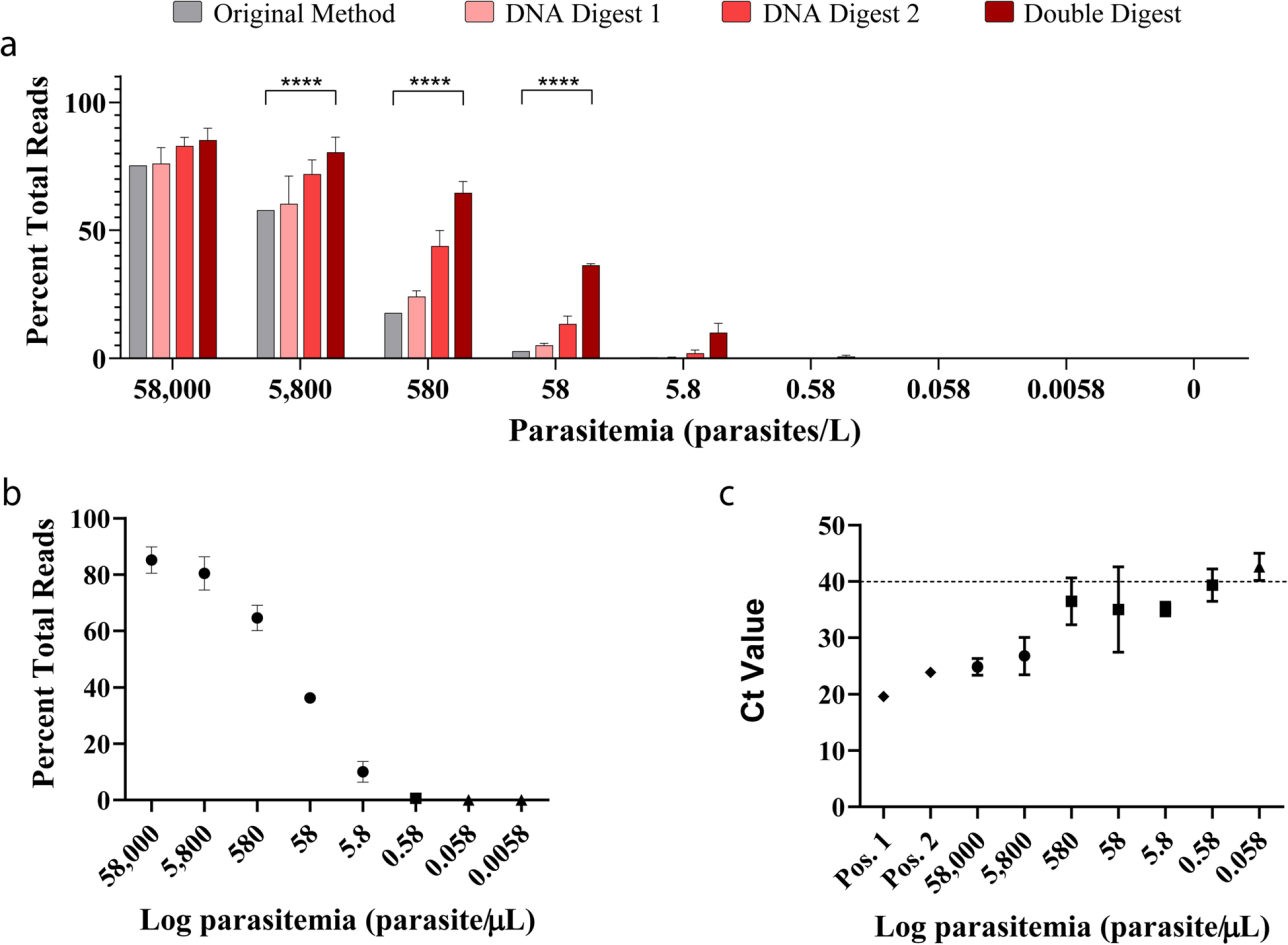
UPDx has a limit of detection similar to standard qPCR. **a** Serial dilutions of *P. falciparum* 3D7 parasites in whole human blood were processed using the original universal parasite detection method (grey bars), the nested method with DNA Digest 1 only (light-colored bars), the nested method with DNA Digest 2 only (medium-colored bars), or the nested method with both DNA Digest 1 and DNA Digest 2 (dark bars). Statistical significance of differences between conditions was assessed using a 2-way ANOVA with Tukey’s multiple comparisons posttest, *p* < 0.0001, *n* = 3, mean ± SD. **b** Nested PCR with both restriction Digest 1 and Digest 2 detected *P. falciparum* at a limit of 0.58 parasites/μL in 2/3 samples (each point is the average of 2 or 3 replicates, error bars are ± 1 SD, circles = parasites were detected in 3/3 replicates, squares = parasites were detected in 2/3 replicates, and triangles = parasites were detected in fewer than 2 replicates). **c** Analysis of the same samples by qPCR demonstrated that positive amplification curves were observed between 0.58 and 5.8 parasites/μL (each point represents the average of three replicates, error bars are ± SD, diamonds are positive controls; Pos. 1 and Pos. 2, circles = detection of parasites in 3/3 replicates, squares = detection of parasites in 2/3 replicates, and triangles = detection of parasites in 0/3 replicates). Percentage total reads refers to the percentage of all reads generated that were used to construct haplotypes identical to *P. falciparum* 18S sequences with the GenBank Accessions XR_002966654.1 and XR_002273081.2

Assessment of our UPDx assay’s limit of detection (LOD), by applying the method to a 10-fold serially diluted blood sample panel containing *Plasmodium falciparum* of known parasitemia (range 58 × 10^3^–0 parasites/μL), indicated that the number of reads mapping to *P. falciparum* reference sequences post-sequencing was highest for the double-digested samples at all concentrations (Fig. 4a, b). The LOD for double-digested samples was approximately 0.58 parasites/μL which is 10-fold lower than the LOD for samples subjected to only D1 or D2 alone and approximately 10-fold more sensitive than the method described by Flaherty et al. [18] (Fig. 4a). For one of the three replicates diluted to 0.58 parasites/μL analyzed using the double digestion UPDx protocol, no parasite reads were detected. Despite this, a direct comparison of the performance of a qPCR routinely used within the Parasitic Diseases Branch at CDC [29] applied to the same serially diluted samples also obtained a positive amplification curve for only 2 of 3 replicates at a concentration of 0.58 parasites/μL (Fig. 4c). Furthermore, the qPCR assay generated a positive amplification curve for only 2 of 3 replicates at all concentrations less than 5800 parasites/μL, while all three replicates were consistently positive using UPDx at all concentrations greater than 0.58 parasites/μL, indicative of a more consistent performance. At a parasite concentration of 5.8 parasites/μL using the UPDx approach, between 400 and 500 parasite-derived reads were detected in each of the three replicates, representing an unambiguous positive result from UPDx; a cutoff of 20 reads was determined for these experiments as previously described [18]. Similarly, for the two positive UPDx results obtained at a parasite concentration of 0.58 parasites/μL, between 100 and 300 parasite reads were detected, which is also indicative of a clear positive result. Additionally, a single replicate at a parasite concentration of 0.058 parasites/μL obtained a positive result using UPDx, though only 31 parasite-derived reads were detected. A plot of the log-transformed data from double-digested specimens also supported a limit of detection of 0.58 parasite/μL, allowing us to make a conservative LOD estimate of near or below 1 parasite/μL for our UPDx method (Fig. 4b).

### UPDx detects and differentiates several parasites commonly found in human blood

**Fig. 5:**
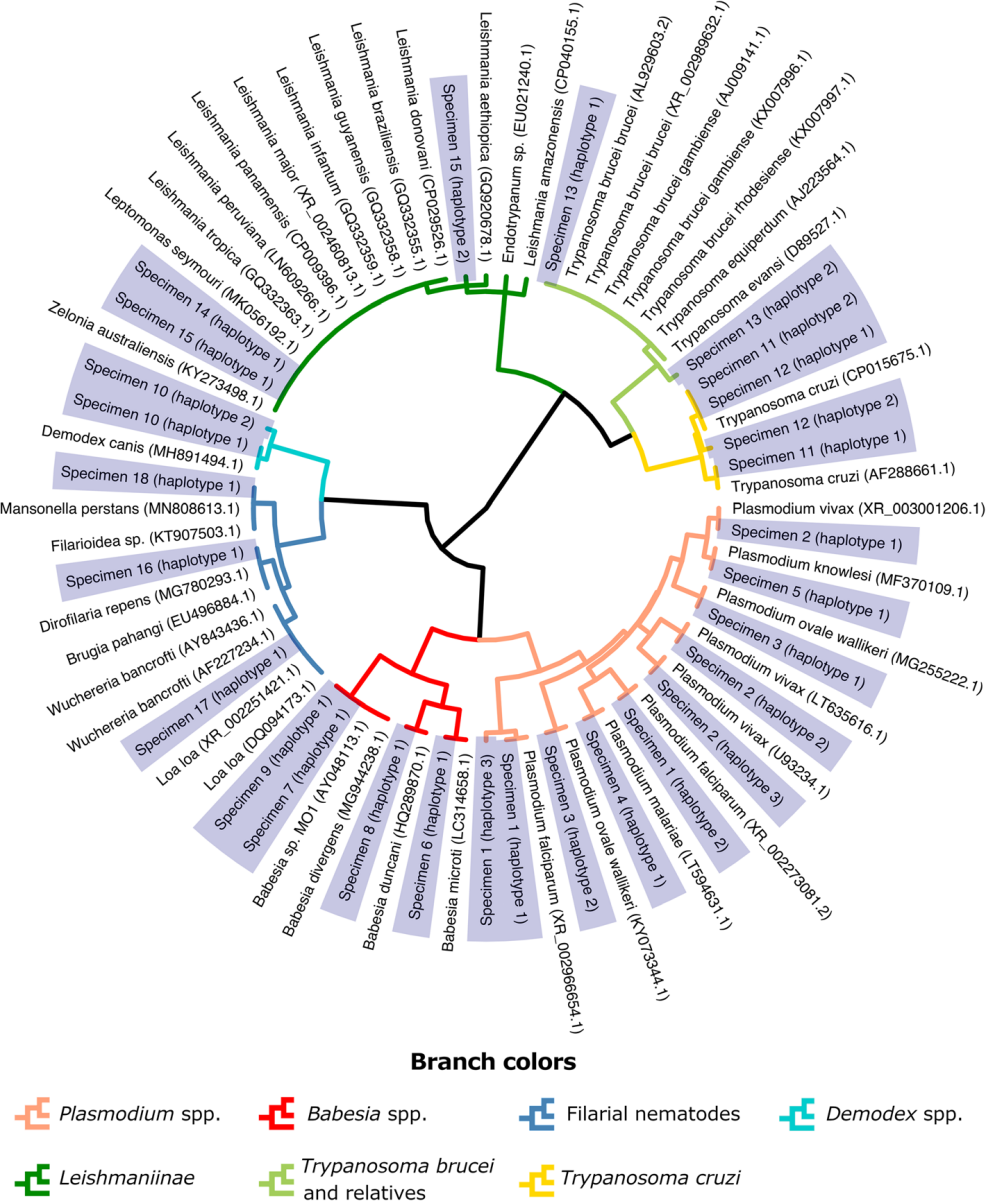
The UPDx amplicon differentiates several taxa of clinically important parasites. Clustering of sequences generated for a range of parasites (Table 2) demonstrates that the UPDx amplicon can differentiate some taxa to the species level, but not all. This segment of the 18S rDNA gene differentiates the most important *Plasmodium* species that infect humans but does not differentiate most *Leishmania* species that infect humans. It does not differentiate subspecies of *T. brucei* from some trypanosomes of veterinary importance, such as *T. evansi* and *T. equiperdum*, but it clearly differentiates *T. cruzi*. Filarial nematodes of the family *Onchocercidae* are differentiated beyond the family level, but not to the genus level. *Babesia* species commonly infecting humans are divided into three sequence types based on the haplotypes detected: one for *Babesia microti*, another for *Babesia duncani*, and a third type that includes *Babesia divergens* and the *B. divergens*-like MO1 type. Sequences generated in this study are shaded blue and include the haplotypes detected in clinical specimens 1 to 18 listed in Table 2

For the simulated infections comprised of various species of parasites, alone or in combination (Tables 1, 2, 3, and 4), all expected parasites were detected with the exception of some specimens expected to contain *T. cruzi* (Tables 2 and 4). This included all major human-infecting *Plasmodium* species (single-species and mixed infections), as well as simulated combinations of *Plasmodium* species, human-infecting kinetoplastids (*T. cruzi* and *Trypanosoma brucei*), and filarial nematodes (*Loa loa* and *Brugia malayi*) (Tables 2, 3, and 4). A clinical blood sample morphologically diagnosed by the CDC Parasite Reference Diagnostic Laboratory as a natural mixed-species malaria infection (*P. falciparum* and *Plasmodium malariae*) tested positive for *P. falciparum* and *P. malariae*, as expected (Table 3). In a separate sequencing experiment, five blood samples from acute *T. cruzi* patients, five from reactivation patients, and one artificial *T. cruzi* sample (whole blood spiked with 2 μL *T. cruzi* culture) were also tested. These specimens were included in this analysis as they had previously been confirmed as positive for *T. cruzi* by the CDC Parasite Reference Diagnostic Laboratory using a previously published qPCR assay (Qvarnstrom 2012). Specimens from patients experiencing *T. cruzi* reactivation each tested negative using the UPDx assay while four of five acute *T. cruzi* samples tested positive (Table 4). The sequences generated using our UPDx assay differentiated all *Plasmodium* species most commonly infecting humans (Fig. 5). Filarial nematodes could be separated into three groups, with finer granularity than the family level (*Onchocercidae*), but not to the genus or species level (Fig. 5). The majority of the human-infecting *Leishmania* species cannot be distinguished based on numerous *Leishmania* 18S sequences in GenBank, though *T. cruzi* and *T. brucei* are clearly distinguished. The three subspecies of *T. brucei* (*rhodesiense*, *gambiense*, and *brucei*) cannot be distinguished from each other nor from several related trypanosomes of veterinary importance included in our BLASTN database (Fig. 5). The three types of *Babesia* most commonly found in humans can be distinguished using our assay: *Babesia microti*, *B. duncani*, and *B. divergens*, with the caveat that sequences belonging to *B. divergens* and the *B. divergens*-like MO1 are identical (Fig. 5).

**Table 4:** Detection of acute clinical *T. cruzi* infections using UPDx with double digestion

Sample	*Tc* reads^a^	Total reads	% Reads	Result
Culture^b^	4496	12,999	34.6%	+
Acute1	64	8549	0.7%	+
Acute2	0	4900	0.0%	−
Acute3	9189	10,731	85.6%	+
Acute4	10,864	13,428	80.9%	+
Acute5	9152	11,161	82.0%	+
Reactivation1	0	7902	0.0%	−
Reactivation2	0	6372	0.0%	−
Reactivation3	0	9592	0.0%	−
Reactivation4	0	9822	0.0%	−
Reactivation5	0	8680	0.0%	−

^a^This is the sum of reads used to construct haplotypes that were identical to *T. cruzi* reference sequences AF288661.1 and/or CP015675.1

^b^Positive control

## Discussion

Arguably, the challenge that is most prohibitive to the widespread application of TADS and other NGS technologies to routine parasite diagnosis is biological in nature. The word “parasite” is a generic term with no taxonomic basis, and its use encompasses a broad range of eukaryotic taxa including certain helminths, protozoa, and some arthropods. The terms “helminth,” “protozoan,” and “arthropod” are also highly generic, with some taxa haphazardly forced together into these groups despite sometimes possessing tenuous evolutionary relationships to each other at best. Consequently, designing PCR assays that specifically amplify “parasite” housekeeping loci while avoiding amplification of vertebrate host DNA is extremely difficult. This is particularly challenging when one considers that for most biological specimens, the mass of host DNA in total DNA extracts will vastly outweigh the mass of parasite DNA. This problem is less so for bacterial pathogens that belong to a different domain of life than the eukaryotes they infect and are therefore sufficiently divergent to avoid off-target amplification of vertebrate DNA.

The present work expands on the observations of Flaherty et al. [18] regarding the benefits of host DNA restriction digestion prior to PCR by introducing several improvements. Firstly, to improve upon the assays’ LOD, we introduced an additional PCR amplification step by converting the assay into a nested PCR approach. The improvement in LOD resulting from this modification is a consequence of the increased number of PCR cycles arising from two rounds of amplification (PCR1 and PCR2) and the introduction of the second digestion step between PCR1 and PCR2, providing a second opportunity to deplete the relative mass of amplifiable host-derived 18S rDNA. This led to a 10-fold increase in LOD compared to the previous method [18], with a LOD comparable to a routinely used qPCR for *P. falciparum*. Furthermore, our UPDx assay performed more consistently than the qPCR assay used here, which failed to detect parasites in one of three replicate samples at all concentrations at or below 580 parasites/μL of blood and obtained negative results for all replicates at a concentration of 0.058 parasites/μL. In contrast, UPDx only failed to detect parasites in one of three replicates at a concentration of 0.58 parasites/μL; all three replicates were positive at concentrations above this. The UPDx assay also detected parasites in one replicate at a concentration of 0.058 parasites/µL and failed to detect parasites at a concentration of 0.0058 parasites/µL. The LOD of our UPDx assay is therefore comparable to other recently published qPCR assays, which possess LODs ranging from 0.03 to 0.3 parasites per microliter of blood [35–37]. In addition to its consistent performance and comparatively low LOD, this assay has the added benefit that all blood parasites present in a sample may be detected.

Instances where our UPDx assay did not result in significant host read reduction compared to control experiments were primarily restricted to infections caused by the microfilariae and *T. cruzi*, wherein initial parasitemia is often exceptionally low. With each PCR cycle, there is a theoretical doubling of template molecules assuming 100% PCR efficiency with no competing/spurious side reactions. For infections with very low parasitemias, the negative impacts of excessive host DNA are more pronounced. Restriction digestion reduces the availability of amplifiable host-derived template DNA, as we clearly demonstrate here, yet even the digested host DNA still competes with parasite DNA amplification because the PCR priming sites are still present and intact on digested host DNA. Consequently, digested host DNA will participate in a competing (albeit inefficient) side reaction, whereby primer is extended after binding to digested host DNA. However, this extension occurs without complete DNA molecule doubling, as it only occurs along a single strand in one direction until it terminates at the restriction digest cut sites. While this anticipated side reaction is incredibly inefficient, under circumstances of lower-level parasitemias, the number of parasite-derived amplicon molecules may never reach a relative frequency that sufficiently outcompetes these spurious side reactions. A solution might be to increase the cycle number for PCR1 and/or PCR2, understanding that the high-fidelity Q5 polymerase we utilize has an exceptionally low error rate; 99.7% of amplicon molecules will have the correct sequence for a 250 base pair amplicon, even after 60 cycles (https://pcrfidelityestimator.neb.com/#!/). While this may increase the assays’ LOD, an increase in PCR cycle number may also make the assay more susceptible to contamination. In addition to increasing the PCR cycle number, the LOD could be further improved by reducing the degree of multiplexing during library preparation (i.e., multiplexing fewer specimens per library). Here, we multiplexed 48 specimens in each library, resulting in approximately 10,000 to 20,000 pairs of reads per specimen, facilitating a LOD within the general range of qPCR assays available for *Plasmodium* sp. (discussed above). Reducing the number of specimens multiplexed per library could improve the LOD, which may be helpful for detecting infections of a low parasitemia. We do recognize however that this would increase the cost of the assay on a per specimen basis, which is approximately $80.00 US per specimen excluding the cost of labor, assuming roughly 40 specimens are multiplexed on the same sequencing run.

Reported error rates for Illumina sequencing are less than 4 erroneous bases for every 1000 bases sequenced [38, 39]. The workflow described here includes a read QC step that removes adapter sequence and quality trims the ends of reads. Any reads less than 50 bases long following trimming are then discarded. Our results support that the read QC process reduced the number of errors in the data that were retained for analysis relative to reported Illumina error rates. This is reflected in the data presented in Table 2, where we report several haplotypes that are approximately 99% identical to their nearest BLAST hit and likely represents sequencing errors. Importantly, these reads only comprise about 2% of the overall number of parasite-derived reads in these specimens following read QC. Furthermore, this single base difference will not confound the diagnosis because the expected amplicons for the parasites examined here typically differ by numerous SNPs and indels, even for the *Plasmodium* species. Therefore, a single SNP induced by a sequencing error will still allow investigators to accurately identify the sequence, leading them to an appropriate diagnosis.

Our method detected several major parasites found in human blood. Using simple conventional PCR and restriction enzyme technology, followed by in-house or outsourced Illumina MiSeq NGS, any laboratory can diagnose all human blood parasites using one test, requiring minimal sample volume, and with a LOD comparable to qPCR. Our UPDx assay and others like it, therefore, have the potential to address the increasing challenge of maintaining difficult-to-learn parasite morphology competency among laboratory staff in endemic regions [40, 41], a task also made difficult in non-endemic regions where blood parasites may only occasionally be encountered in returned travelers [42, 43]. Due to the relatively high cost of NGS, UPDx in the form described here is most appropriate for adoption by state and national parasitic disease reference laboratories, to which state and regional laboratories often refer diagnostically challenging cases. However, the possibility of adapting the assay to more compact Illumina platforms (i.e., MiniSeq and/or iSeq) could make the assay more accessible to other laboratories. While these platforms produce shorter reads than the MiSeq—a maximum of 150 base pair paired end reads—this is still compatible with the short amplicon utilized for UPDx. In addition, cost reduction by introducing adapter and index sequences to amplicons during the UPDx PCR steps is currently being explored, as this will remove the need for expensive, laborious, and time-consuming Illumina library preparations. This would also make the assay drastically cheaper and less complicated to prepare and reduce diagnostic turnaround times.

In this study, we utilize the same cutoff system described by Flaherty et al. [18], which utilized a “hard minimum” cutoff of 20 parasite-derived reads (below which a specimen is considered negative), and an adaptable “sliding maximum” that must be calculated each time a library is sequenced and requires that each sequencing run include at least three known negative blood specimens. This sliding maximum was introduced to control for index cross-talk resulting from specimen multiplexing, which leads to a variable number of parasite-derived reads being assigned to the negative control specimens when the data are de-multiplexed. The number of parasite-derived reads assigned to the negatives as a result of this “cross-talk” varies depending on the specimens included in the library preparation. For example, for a library where 35 of 40 specimens are positive for *P. falciparum*, the negative control specimens will almost certainly be assigned a small number of *P. falciparum* reads. Alternatively, if every specimen in a run is negative, index cross-talk is still occurring, although very few (or no) negative control specimens will contain parasite-derived reads, because the only source of these reads would be the positive control specimen included in each run. For further detail, please refer to the work of Flaherty and colleagues [18]. In our experience, the distinction between the commonly occurring low-level index cross-talk and a reagent contamination event is clear when the data are examined. Following a true reagent contamination event, the negative control specimens (and indeed many specimens) contain hundreds to thousands of “off-target” parasite-derived reads, requiring preparation of a new library, whereas index cross-talk will—on average—result in approximately 20 off-target parasite-derived reads in the negative control specimens [18].

Assays similar to the UPDx system have been described. Cannon et al. [44] developed a pan-parasite assay requiring multiplexing of thirteen primer pairs that distinguishes a broad range of parasites, including the microsporidia. The amplicons generated by this assay range from between 200 and 450 bases (depending on the taxon) which is comparable to UPDx (~ 200 bases for all taxa) [44]. An important difference between the UPDx assay and the one described by Cannon et al. is the use of a single primer set for UPDx that captures a smaller amplicon to improve amplification efficiency. To achieve an analytical sensitivity (LOD) similar to qPCR (as was deemed necessary for a diagnostic test), UPDx also incorporates a nested PCR step. Given CDC’s role as a public health agency, the analytical sensitivity was an important consideration for UPDx, as was obtaining a sufficient level of discriminatory power—at least enough to inform clinical decisions. The assay described by Cannon et al. [44] may possess additional discriminatory power compared to the UPDx assay as it generates larger amplicons, perhaps at the expense of its analytical sensitivity—though this requires experimental substantiation—and assay simplicity, given it required multiplexing of several targets. Regardless, the issue of multiplexing multiple targets may become less challenging with the introduction of novel multiplexing technologies, such as the CleanPlex protocol utilized by Tessema et al. [45] to amplify 100 targets from dried blood spots for characterization of *P. falciparum* genotypes. Schwabl and colleagues were able to multiplex 203 primers pairs within the same PCR reaction prior to Illumina sequencing [46], though this number of targets is likely unnecessary for a diagnostic assay and would almost certainly have a negative impact on the assays’ LOD. In any case, in line with these developments, the addition of different molecular targets to the assay described here is being explored for future iterations, to provide additional discriminatory power for certain taxa (discussed below) where this is limited by the short length of our UPDx amplicon.

One limitation of the UPDx assay is that it does not differentiate every parasite to the species level and cannot differentiate certain taxa to the genus level; the level of differentiation is taxon dependent. For example, the main malaria parasites of humans can each be differentiated to the species level, with the added benefit that these species each possess at least two distinct 18S rDNA types, providing additional granularity (Table 2 and Fig. 5). *Babesia* were divided among 3 groups: one that includes *B. microti*, another including *B. duncani*, and the third containing *B. divergens* and *B. divergens*-like MO1 parasites. While we cannot exclude the possibility that spurious infections caused by rare, zoonotic *Babesia* species cannot be distinguished from any of these three groups, the main *Babesia* species found in humans can be differentiated from each other. The filarial nematodes can be differentiated beyond the family level (*Onchocercidae*) but not to the genus level; *Wuchereria* and *Loa* produce amplicons that are indistinguishable. Furthermore, a second group of identical sequences included a sequence from *Brugia pahangi* that was identical to a sequence from a clinical specimen containing *B. malayi* (Table 2, specimen 16), and a sequence from *Dirofilaria repens*. Amplicons generated for *Mansonella perstans* (Table 2, sample 18) are identical to other *Filarioidea* sequences including a sequence from *Dipetalonema* sp. (GenBank accession DQ531723.1), which is, however, of veterinary significance but was included here for comparison. These parasites yielding indistinguishable amplicons may be differentiated either by their differing geographical distribution or using a specific PCR for any sympatric species.

The differentiation potential of UPDx is probably lowest for the kinetoplastids, the most clinically important of these being various *Leishmania* species, *T. cruzi*, and *T. brucei*, which are the causative agents of leishmaniasis, Chagas disease, and African Sleeping Sickness (Human African Trypanosomiasis), respectively. Using the amplicon sequences captured by UPDx (Fig. 5), most human-infecting *Leishmania* species cannot be distinguished. However, these parasites also cannot be differentiated morphologically, so downstream molecular testing is required for a species-level diagnosis when *Leishmania* is observed microscopically in culture or tissue biopsies. We note that other monoxenous trypanosomatids of the subfamily *Leishmaniinae* (e.g., *Leptomonas*) cannot be differentiated from *Leishmania* species by UPDx, though the *Leishmaniinae* that infect humans are almost exclusively members of the genus *Leishmania*, aside from rare circumstances where infections caused by monoxenous species have been reported [47]. The *Leishmaniinae* are unambiguously distinguishable from the other kinetoplastids infecting humans—*T. brucei* and *T. cruzi*. Furthermore, *Trypanosoma cruzi* is clearly separated from *T. brucei*, although *T. b. gambiense* and *T. b. rhodesiense* cannot be distinguished. Similarly, the sequence generated for *T. cruzi* distinguishes it from the closely related *Trypanosoma rangeli* based on *T. rangeli* sequences available in GenBank (accessions: XR_003828669.1, KJ742907.1, FJ900242.2, AY491767.1, AJ012416.1, AJ012414.1, AJ009160.1, AF065157.1), each of which possess a single SNP and an indel differentiating them from the homologous *T. cruzi* amplicon sequences. The UPDx haplotype for *T. brucei* is identical to that predicted for other trypanosomes of primarily veterinary importance, such as *T. b. brucei*, *T. equiperdum*, and *T. evansi* (Fig. 5). Although *T. b. brucei* and *T. evansi* may very rarely cause human disease [48, 49], these are considered to be aberrant and often self-limiting events [48]. In most cases, these trypanosomes can be differentiated based on allopatric geographical range and differing clinical features. Despite this limitation, the amplicon captured by UPDx can potentially discriminate between a broad range of parasite taxa including numerous protozoa, nematodes, cestodes, and trematodes based on preliminary analyses of sequences available in GenBank (Supplementary file S1, Appendix B).

The size of the external nested amplicon (approximately 2 kb) represents another limitation of this assay owing to the difficulty in identifying priming sites conserved across the many diverse parasite taxa. It is acknowledged that PCR amplification efficiency is lower for longer amplicons, and when the assay is applied to DNA extracts of a lesser quality, such as extracts from formalin-fixed specimens, or DNA extracts that were incorrectly stored, a slight loss of sensitivity may be observed. However, in instances where DNA is extracted from correctly stored, unpreserved (non-formalin-fixed) specimens, the assays’ performance should reflect the results described here.

Despite its limitations, the UPDx assay described here shows great potential for routine diagnostic use. It offers a single test for universal blood parasite detection that is comparable in LOD to qPCR, making it more amenable to routine use than the assay previously described by Flaherty et al. [18]. Aside from detecting multiple parasite species, the diagnostic result is objective; separation of some taxa is not always achieved, but the assay is not subject to false positive results that may result from spurious off-target amplifications, for example. A major benefit of UPDx is that no prescient knowledge of the potential causative agent is required, which is helpful in complex clinical scenarios where the appropriate path for a differential diagnosis might be elusive. The UPDx primer sets should also amplify parasitic agents from mammals, birds, and reptiles. Furthermore, the restriction sites utilized are seemingly conserved across all vertebrates. We confirmed via extensive sequence alignments that even across different vertebrate classes the expected internal amplicon is between 98 and 100% identical possessing one or both of the necessary BamHI and/or XmaI restriction sites, and the PstI restriction site was present in the majority of reptile, bird, amphibian, fish (cartilaginous and bony), and mammalian 18S sequences examined. Consequently, UPDx will likely be generalizable to a large number of animal species.

## Conclusions

This work expands on the observations of Flaherty et al. [18] regarding the benefits of host DNA restriction digestion prior to PCR by introducing several novel modifications intended to improve the utility of TADS for parasite detection and characterization of parasite communities in biological matrices derived from vertebrates. These modifications improved the detection limit for human blood parasites by approximately 10-fold, compared to the earlier assay, to a level that is comparable with real-time PCR. As a consequence of this improved detection limit, the potential adoption of UPDx to routine diagnostic settings is currently being explored. Due to the complexity of UPDx and the relatively high cost of NGS, this assay is currently most appropriate for adoption by state and national parasitic disease reference laboratories, to which state and regional laboratories often refer “difficult” cases that are diagnostically challenging; it is in these circumstances where UPDx offers a clear benefit. This assay is currently being validated for routine use at the Centers for Disease Control and Prevention, and New York State (Wadsworth) Parasitic Disease Reference Laboratories. With continued development including novel library preparation strategies to reduce setup time and running costs, our UPDx has the potential to become increasingly amenable to the routine diagnosis of parasitic pathogens commonly found in human blood.

## Supplementary Information

**Additional file 1.**

## Data Availability

All sequence data generated as part of this study is available in the SRA database accessible through NCBI. These have been deposited under BioProject accession number PRJNA437674. Specimens relevant to the present study submitted under this BioProject include the text “nUPDx method validation” in their title.

## Abbreviations

CDC: Centers for Disease Control and Prevention
EDTA: Ethylenediaminetetraacetic acid
NGS: Next-generation sequencing
TADS: Targeted amplicon deep sequencing
UPDx: Universal parasite diagnostic
LOD: Limit of detection
PCR: Polymerase chain reaction
qPCR: Quantitative PCR
