# Supplementary material for: Sensitive universal detection of blood parasites by selective pathogen-DNA enrichment and deep amplicon sequencing
Source: Microbiome. 2021 Jan 2;9:1. doi: 10.1186/s40168-020-00939-1 (PMC7778815; doi:10.1186/s40168-020-00939-1)
Supplement: Supplementary file 2 — Additional file 1. [file 40168_2020_939_MOESM1_ESM.pdf]

Supplementary File S1. Technical notes for manuscript:

Sensitive universal detection of blood parasites by selective pathogen-DNA enrichment and deep amplicon sequencing

Briana R. Flaherty[†1,2], Joel Barratt[†1,2]*, Meredith Lane[1,3], Eldin Talundzic[4], Richard S. Bradbury[1,5]

[1]Parasitic Diseases Branch, Division of Parasitic Diseases and Malaria, Center for Global Health, Centers for Disease Control and Prevention, Atlanta, GA, USA.

[2]Oak Ridge Institute for Science and Education, Oak ridge, TN, USA.

[3]Synergy America Inc.

[4]Malaria Branch, Division of Parasitic Diseases and Malaria, Center for Global Health, Centers for Disease Control and Prevention, Atlanta, GA, USA.

[5]School of Health and Life Sciences, Federation University, Australia

***Corresponding authors**

nsk9@cdc.gov, jbarratt@cdc.gov

r.bradbury@federation.edu.au

[†]Authors contributed equally

This document provides additional detail on the experiments performed to optimize our universal parasite diagnostic (UPDx) assay.

Additional notes on UPDx optimization

Digestion of neat DNA versus normalized DNA and exclusion of DNA cleanup following digestion

To reduce time and labor requirements, the present UPDx method was optimized for minimal host-derived read recovery and to maximize parasite read recovery. The method described by Flaherty and colleagues involved subjecting a normalized mass of DNA to enzyme digestion (150 ng), followed by a DNA cleanup step that removed restriction enzymes and buffers prior to PCR (Flaherty et al. 2018). While the 150 ng digest was performed for the sake of consistency across samples, it added an additional laborious step. Here, we subjected neat DNA extracts to restriction digestion and did not perform a DNA cleanup step after D1 and/or D2, removing multiple extraneous steps. Exclusion of these steps sometimes enhanced parasite DNA detection, as a loss of DNA during the additional cleanup was avoided; we tested the impact of these alterations on blood containing *P. knowlesi* and *L. infantum*, confirming that these extraneous cleanup procedures resulted in a loss of performance. The number of *P. knowlesi* reads recovered increased when neat DNA vs quantified and diluted DNA (to 150 ng) was tested, though results obtained for *L. infantum* showed little difference. Similarly, cleanup of DNA following restriction digestion and before PCR1 greatly reduced the subsequent number of *P. knowlesi* reads recovered following NGS, but this made little difference for *L. infantum* (Figure S1 - below).

Digestion of DNA extracts in CutSmart Buffer versus water

As part of our assay optimization procedures, the impact of digesting DNA in commercially available CutSmart Buffer (NEB) versus water was also assessed using blood specimens containing *P. knowlesi* and *L. infantum* extracted into Buffer EB. The addition of CutSmart buffer at 1x concentration to the DNA eluate approximately doubled the resultant

number of *P. knowlesi* reads and led to an almost 20 times increase in total *L. infantum* reads, confirming that utilization of CutSmart Buffer, or an equivalent buffer, during restriction enzyme digestion is essential (Figure S1 - below).

The impact of cycle number for PCR1 and length of restriction digest times (D1 and D2)

We observed the recovery of substantially more parasite-specific reads for samples that underwent nested PCR amplification with 15 cycles compared to 10 cycles for PCR1, and when both DNA Digest 1 and DNA Digest 2 (double digestions) were performed, compared to only one of these digestion steps (Figure S1 - below). Restriction digestions were optimized by comparing different digestion times (15 minutes, 1 hour, 2 hours, overnight, or not at all) for both digests (D1 and D2). Restriction digest duration generally had a minor impact beyond 15 minutes. Consequently, the optimal protocol requires Digest 1 for 15 minutes, running PCR1 for 15 cycles, and performing Digest 2 for two hours; though this digest time may be shortened slightly without a major loss of performance.

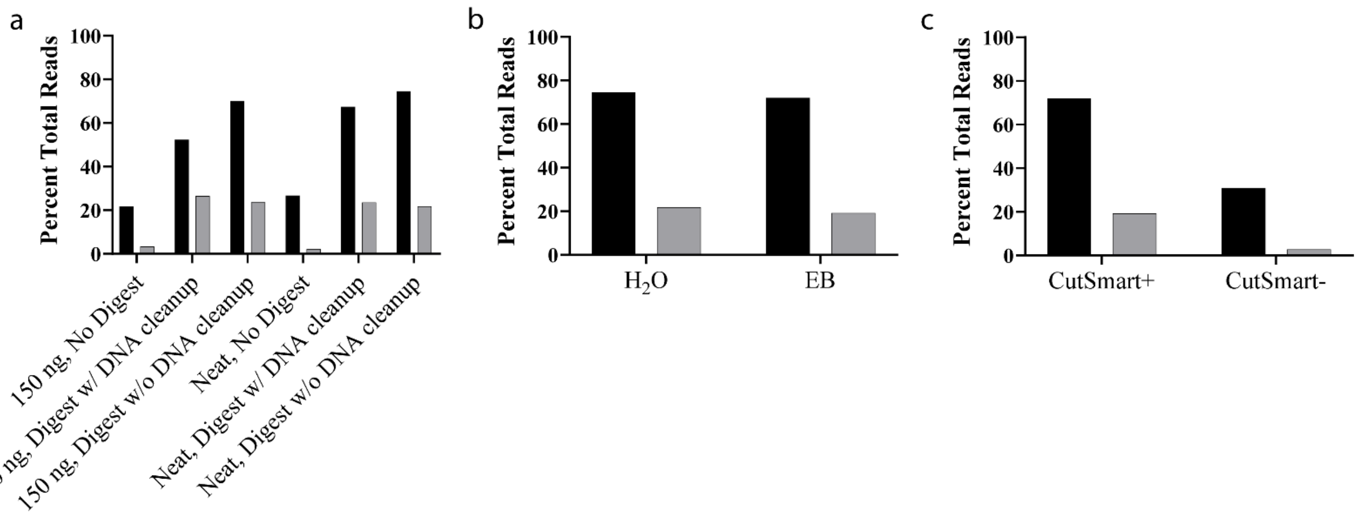

d

Digest 1 (minutes)	Digest 2 (minutes)	*P. falciparum* No PCR1 (original method)		*P. falciparum* 10-cycle PCR1		*P. falciparum* 15-cycle PCR1		*L. infantum* No PCR1 (original method)		*L. infantum* 10-cycle PCR1		*L. infantum* 15-cycle PCR1	
	120	89.6%	2.6%					41.3%	44.6%				
ND	ND			57.4%	32.4%	41.0%	47.5%			1.8%	89.8%	2.2%	91.8%
ND	15			85.1%	6.1%	83.7%	6.6%			10.3%	74.3%	32.7%	55.9%
ND	60			79.8%	10.8%	85.7%	5.3%			11.7%	71.2%	33.3%	55.6%
15	ND			78.6%	11.2%	83.0%	8.4%			5.3%	84.5%	37.9%	51.2%
15	15			88.1%	3.7%	92.3%	0.4%			14.8%	73.4%	67.3%	22.5%
15	60			87.4%	3.2%	92.3%	0.5%			16.0%	72.3%	65.8%	24.4%
60	ND			77.7%	12.9%	84.3%	7.4%			7.9%	82.0%	40.4%	49.3%
60	15			89.9%	3.2%	92.3%	0.6%			17.2%	70.5%	72.6%	19.2%
60	60			85.1%	3.8%	91.8%	0.4%			19.5%	67.8%	69.6%	21.1%
120	ND			79.2%	12.0%	85.3%	6.3%			7.7%	82.0%	40.2%	48.2%
120	15			90.9%	2.4%	92.9%	0.4%			18.3%	70.8%	78.6%	14.4%
120	60			87.0%	3.6%	92.1%	0.4%			17.7%	67.7%	75.0%	17.8%
ON	ND			76.6%	12.1%	87.2%	4.1%			6.9%	82.3%	45.5%	44.9%
ON	15			83.4%	8.1%	93.1%	0.3%			17.4%	71.0%	79.4%	14.2%
ON	60			88.4%	3.1%	92.3%	0.2%			18.3%	68.8%	73.5%	18.4%

Figure S1. Overview of optimization experiments performed for our UPDx assay

(**a**) 150 ng or 25 µL of neat *P. knowlesi* (black bars) and *L. infantum* (grey bars) DNA extracts in molecular grade water were digested with 10 units of BamHI-HF and BsoBI in 1X CutSmart Buffer for 2 hours. Samples were transferred directly to PCR2 or cleaned before PCR2 amplification and DNA sequencing. No notable difference between 150 ng and neat digestion was observed; DNA cleanup had negative or minimal effects on the final proportion of parasite-derived reads recovered in the sample. (**b**) 25 µL of neat *P. knowlesi* (black bars) and *L. infatum* (grey bars) DNA extracts eluted into molecular grade water (H_2O) or elution buffer (EB) were digested with 10 units of BamHI-HF and BsoBI in 1X CutSmart Buffer for 2 hours and transferred directly to PCR2 for amplification and sequencing. No notable difference was observed between samples eluted into water versus elution buffer. (**c**) 25 µL of neat *P. knowlesi* (black bars) or *L. infantum* (grey bars) DNA extracts in elution buffer were digested with 10 units of BamHI-HF and BsoBI in the presence or absence of 1X CutSmart Buffer for 2 hours and transferred to PCR2 for amplification and sequencing. Substantially higher parasite-specific reads, indicating more efficient digestion of host DNA, were observed for sample digested in the presence of CutSmart buffer. (**d**) DNA extracts of neat *P. falciparum* or *L. infantum* in elution buffer were processed by the original method or one of a variety of conditions including (a) digestion with 10 units PstI-HF for 0, 15, 60, 120 minutes, or overnight (b) PCR1 for 10 or 15 cycles, and (c) digestion with 10 units BamHI-HF and BsoBI for 0, 15, or 60 minutes. Samples were then amplified by PCR2 and sequenced. Optimal results were achieved with 15-cycle PCR1 and any combination of pre-PCR1 (Digest 1) and pre-PCR2 (Digest 2) digestion (n = 1, ND = no digest).

Table S1. Preparation of simulated mixed blood parasite infections

Sample Name	Spiked Analytes	DNA extract of specimen from Table 1*	Volumes of DNA extract mixed	Total volume of final simulated mixture
**Mix 1**	***P. falciparum***	Specimen 1	4 µL	8 µL
	P. vivax	Specimen 2	4 µL	
**Mix 2**	***P. ovale***	Specimen 3	4 µL	8 µL
	P. falciparum	Specimen 1	4 µL	
**Mix 3**	***P. falciparum***	Specimen 1	4 µL	8 µL
	P. malariae	Specimen 4	4 µL	
**Mix 4**	***P. falciparum***	Specimen 1	4 µL	8 µL
	P. knowlesi	Specimen 5	4 µL	
**Mix 5**	***P. falciparum***	Specimen 1	2.2 µL	8 µL
	P. vivax	Specimen 2	2.2 µL	
	P. ovale	Specimen 3	2.2 µL	
	P. malariae	Specimen 4	2.2 µL	
	P. knowlesi	Specimen 5	2.2 µL	
**Mix 6**	***P. falciparum***	Specimen 1	4 µL	8 µL
	T. cruzi	Specimen 10	4 µL	
**Mix 7**	***P. vivax***	Specimen 2	4 µL	8 µL
	T. cruzi	Specimen 10	4 µL	
**Mix 8**	***P. falciparum***	Specimen 1	4 µL	8 µL
	T. brucei	Specimen 13	4 µL	
**Mix 9**	***P. falciparum***	Specimen 1	4 µL	8 µL
	L. loa	Specimen 17	4 µL	
**Mix 10**	***P. falciparum***	Specimen 1	4 µL	8 µL
	B. malayi	Specimen 16	4 µL	

To produce these simulated mixed infections, DNA extracts from various specimens listed in Table 1 from the main manuscript text* were mixed as described in this table. Prior to testing with UPDx, 3 µL of Qiagen elution buffer was added to all 8 µL of these mixtures (mixes 1 to 10) to bring the final volume to 11 µL. This addition of elution buffer was performed to add extra volume to these mixes (while conserving the original extracts), allowing for sample loss due to evaporation during storage, and to bring the volume of the mix above 8.5 µL. When required for UPDx testing, 8.5 µL of these DNA solutions was processed according to the methods described in the main manuscript text.

Appendix A. Fasta sequences that were aligned to facilitate the design of our 'outer' nested primers

```
>HUMRGE - Human 18S rRNA gene, complete
CCGTCCGTCCGTCGTCCTCCTCGCTTGCGGGGCGCCGGGCCCGTCCTCGAGCCCCCNNNNNCCGTCCGGCCGCGTCGGGG
CCTCGCCGCGCTCTACCTACCTACCTGGTTGATCCTGCCAGTAGCATATGCTTGTCTCAAAGATTAAGCCATGCATGTCT
AAGTACGCACGGCCGGTACAGTGAAACTGCGAATGGCTCATTAAATCAGTTATGGTTCCTTTGGTCGCTCGCTCCTCTCC
TACTTGGATAACTGTGGTAATTCTAGAGCTAATACATGCCGACGGGCGCTGACCCCCTTCGCGGGGGGGATGCGTGCATT
TATCAGATCAAAACCAACCCGGTCAGCCCCTCTCCGGCCCCGGCCGGGGGGCGGGCCGCGGCGGCTTTGGTGACTCTAGA
TAACCTCGGGCCGATCGCACGCCCCCCGTGGCGGCGACGACCCATTCGAACGTCTGCCCTATCAACTTTCGATGGTAGTC
GCCGTGCCTACCATGGTGACCACGGGTGACGGGGAATCAGGGTTCGATTCCGGAGAGGGAGCCTGAGAAACGGCTACCAC
ATCCAAGGAAGGCAGCAGGCGCGCAAATTACCCACTCCCGACCCGGGGAGGTAGTGACGAAAAATAACAATACAGGACTC
TTTCGAGGCCCTGTAATTGGAATGAGTCCACTTTAAATCCTTTAACGAGGATCCATTGGAGGGCAAGTCTGGTGCCAGCA
GCCGCGGTAATTCCAGCTCCAATAGCGTATATTAAAGTTGCTGCAGTTAAAAAGCTCGTAGTTGGATCTTGGGAGCGGGC
GGGCGGTCCGCCGCGAGGCGAGCCACCGCCCGTCCCCGCCCCTTGCCTCTCGGCGCCCCCTCGATGCTCTTAGCTGAGTG
TCCCGCGGGGCCCGAAGCGTTTACTTTGAAAAAATTAGAGTGTTCAAAGCAGGCCCGAGCCGCCTGGATACCGCAGCTAG
GAATAATGGAATAGGACCGCGGTTCTATTTTGTTGGTTTTCGGAACTGAGGCCATGATTAAGAGGGACGGCCGGGGGCAT
TCGTATTGCGCCGCTAGAGGTGAAATTCTTGGACCGGCGCAAGACGGACCAGAGCGAAAGCATTTGCCAAGAATGTTTTC
ATTAATCAAGAACGAAAGTCGGAGGTTCGAAGACGATCAGATACCGTCGTAGTTCCGACCATAAACGATGCCGACCGGCG
ATGCGGCGGCGTTATTCCCATGACCCGCCGGGCAGCTTCCGGGAAACCAAAGTCTTTGGGTTCCGGGGGGAGTATGGTTG
CAAAGCTGAAACTTAAAGGAATTGACGGAAGGGCACCACCAGGAGTGGAGCCTGCGGCTTAATTTGACTCAACACGGGAA
ACCTCACCCGGCCCGGACACGGACAGGATTGACAGATTGATAGCTCTTTCTCGATTCCGTGGGTGGTGGTGCATGGCCGT
TCTTAGTTGGTGGAGCGATTTGTCTGGTTAATTCCGATAACGAACGAGACTCTGGCATGCTAACTAGTTACGCGACCCCC
GAGCGGTCGGCGTCCCCCAACTTCTTAGAGGGACAAGTGGCGTTCAGCCACCCGAGATTGAGCAATAACAGGTCTGTGAT
GCCCTTAGATGTCCGGGGCTGCACGCGCGCTACACTGACTGGCTCAGCGTGTGCCTACCCTACGCCGGCAGGCGCGGGTA
ACCCGTTGAACCCCATTCGTGATGGGGATCGGGGATTGCAATTATTCCCCATGAACGAGGAATTCCCAGTAAGTGCGGGT
CATAAGCTTGCGTTGATTAAGTCCCTGCCCTTTGTACACACCGCCCGTCGCTACTACCGATTGGATGGTTTAGTGAGGCC
CTCGGATCGGCCCCGCCGGGGTCGGCCCACGGCCTGGCGGAGCGCTGAGAAGACGGTCGAACTTGACTATCTAGAGGAAG
TAAAAGTCGTAACAAGGTTTCCGTAGGTGAACCTGCGGAAGGATCATTA
>AJ439713 - Babesia divergens 18S rRNA gene
AACCTGGTTGATCCTGCCAGTAGTCATATGCTTGTCTTAAAGATTAAGCCATGCATGTCTAAGTACAAACTTTTTACGGT
GAAACTGCGAATGGCTCATTACAACAGTTATAGTTTCTTTGGTATTCGTTTTCCATGGATAACCGTGCTAATTGTAGGGC
TAATACAAGTTCGAGGCCTTTTGGCGGCGTTTATTAGTTCTAAAACCATCCCTTTTGGTTTTCGGTGATTCATAATAAAC
TTGCGAATCGCAATTTTTTGCGATGGACCACTCAAGTTTCTGACCCATCAGCTTGACGGTAGGGTATTGGCCTACCGAGG
CAGCAACGGGTAACGGGGAATTAGGGTTCGATTCCGGAGAGGGAGCCTGAGAAACGGCTACCACATCCAAGGAAGGCAGC
AGGCGCGCAAATTACCCAATCCTGACACAGGGAGGTAGTGACAAGAAATAACAATACAGGGCAATTGTCTTGTAATTGGA
ATGATGGTGACCTAAACCCTCACCAGAGTAACAATTGGAGGGCAAGTCTGGTGCCAGCAGCCGCGGTAATTCCAGCTCCA
ATAGCGTATATTAAACTTGTTGCAGTTAAAAAGCTCGTAGTTGAATTTTTGCGTGGTGTTAATATTGACTGATGTCGAGA
TTGCACTTCTCTTTTGGGATTTATCCCTTTTTACTTTGATAAAATTAGAGTGTTTCAAGCAGACTTTTGTCTTGAATACT
TCAGCATGGAATAATAGAGTAGGACTTTGGTTCTATTTTGTTGGTTTGTGAACCTTAGTAATGGTTAATAGGAACGGTTG
GGGGCATTCGTATTTAACTGTCAGAGGTGAAATTCTTAGATTTGTTAAAGACGAACTACTGCGAAAGCATTTGCCAAGGA
CGTTTTCATTAATCAAGAACGAAAGTTAGGGGATCGAAGACGATCAGATTCCGTCGTAGTCCTAACCATAAACTATGCCG
ACTAGGGATTGGAGGTCGTCATTTTTTCGACTCCTTCAGCACCTCGAGAGAAATCAAAGTCTTTGGGTTCTGGGGGGAGT
ATGGTCGCAAGGCCGGAACTTAAAGGAATTGACGGAAGGGCACCACCAGGCGTGGAGCCTGCGGCTTAATTTGACTCAAC
GCGGGGAAACTCACCAGGTCCAGACAATGTTAGGATTGACAGATTGATAGCTCTTTCTTGATTCTTTGGGTGGTGGTGCA
TGGCCGTTCTTAGTTGGTGGAGTGATTTGTCTGGTTAATTCCGTTAACGAACGAGACCTTAACCTGCTAACTAGTGTCCG
TAAAAAGGTTCGTCCGTTACGGTTTGCTTCTTAGAGGGACTTTGCGGCTCTAAGCCGCAAGGAAGTTTAAGGCAATAACA
GGTCTGTGATGCCCTTAGATGTCCTGGGCTGCACGCGCGCTACACTGATGCATTCATCGAGTTTTATCCCTTCCCGAAAG
GGCTGGGTAATCTTTAGTATGCATCGTGACGGGGATTGATTTTTGCAATTCTAAATCATGAACGAGGAATGCCTAGTATG
CGCAAGTCATCAGCTTGTGCAGATTACGTCCCTGCCCTTTGTACACACCGCCCGTTGCTCCTACCGATCGAGTGATCCGG
TGAATTATTCGGACCGTGGCCTTTCCGATTCGTCGGTTTGGCCTAGGGAAGTCTTGTGAACCTTATCACTTAGAGGAAAG
AGAAGTCGTAACAAGGTTTCCGTAGGTGAACCTGCAGAAGGATCAAGC
>HQ289870 - Babesia duncani isolate BAB1615 18S ribosomal RNA gene, complete sequence
AACCTGGTTGATCCTGCCAGTAGTCATATGCTTGTCTTAAAGATTAAGCCATGCATGTCTAAGTATAAACTTTTATATGG
TGAAACTGCGAATGGCTCATTACAACAGTTATAGTTTATTTGAAAGTCGTTTTTACATGGATAACCGTGCTAATTGTAGG
GCTAATACATGCTCGAGGCCTTGGCTTCTGTCTTGGCTGCGTTTATTAGACTCGAAACCTTCCCGCTTGCGGTACTCGGT
GATTCATAATAAATTTGCGAATCGCATGGCTTTTGCCGGCGATGGTTCATTCAAGTTTCTGACCTATCAGCTTTGGACGG
TAGGGTATTGGCCTACCGGGGCAGCGACGGGTAACGGGGAATTAGGGTTCGATTCCGGAGAGGGAGCCTGAGAAACGGCT
ACCACATCTAAGGAAGGCAGCAGGCGCGCAAATTACCCAATACGGACACCGTGAGGTAGTGACAAGAAATAACAATACAG
GGCTTAAAGCTTTGTAATTGGAATGATGGGAATCCAAACCCCTTCCAGAGTATCAATTGGAGGGCAAGTCTGGTGCCAGC
AGCCGCGGTAATTCCAGCTCCAATAGCGTATATTAAACTTGTTGCAGTTAAAAAGCTCGTAGTTGAACTTCTGCCGCTTG
GCCTTCGTTCCCCTTGGGGTTTCGTTCGCCTGGTGGCTTACCTCTGGCGGTGGTTCTCCATTTGCCAGTTTTACTTTGAG
AAAATTAGAGTGTTTCAAGCAGGCTTTTGCCTTGAATACTTCAGCATGGAATAATAAAGTAGGACTTTGGTTCTATTTTG
TTGGTTTCAGGACCAAAGTAATGGTTAATAGGAACAGTTGGGGGCATTCGTATTTAACTGTCAGAGGTGAAATTCTTAGA
TTTGTTAAAGACGAACTACTGCGAAAGCATTTGCCAAGGATGTTTTCATTAATCAAGAACGAAAGTTAGGGGCTCGAAGA
CGATCAGATACCGTCGTAGTCCTAACTATAAACTATGCCGACTAGAGATTGGAGGTCGTCATTTTAAACGACTCCTTCAG
CACCTTGAGAGAAATCAAAGTCTTTGGGTTCTGGGGGGAGTATGGTCGCAAGGCTGAAACTTAAAGGAATTGACGGAAGG
GCACCACCAGGCGTGGAGCCTGCGGCTTAATTTGACTCAACACGGGGAACCTCACCAGGTCCAGACATAGTTAGGATTGA
```

CAGATTGATAGCTCTTTCTTGATTCTATGGGTAGTGGTGCATGGCCGTTCTTAGTTGGTGGAGTGATTTGTCTGGTTAAT
TCCGTTAACGAACGAGACCTTAACCTGCTAAATAGCAGCTGAGAATAATCTCTTGTTTCAGTTTTGCTTCTTAGAGGGAC
TTTGCGGTCATAAATCGCAAGGAAGTTTAAGGCAATAACAGGTCTGTGATGCCCTTAGATGTCCTGGGCTGCACGCGCGC
TACACTGATGCATTCATCGAGTTTTATCCTTGCCCGAAAGGGTTTGGTAATCTTTAGTATGCATCGTGATGGGGATTGAT
TATTGCAATTATTAATCATGAACGAGGAATGCCTAGTAGGCGCGAGTCATCAGCTCGTGCCGACTACGTCCCTGCCCTTT
GTACACACCGCCCGTCGCTCCTACCGATCGAGTGATCCGGTGAATTATTCGGACCGTGACGCTTCTAATTCGTTAGAAAT
GTCTAGGGAAGTTTTGTGAACCTTATCACTTAAAGGAAGGAGAAGTCGTAACAAGGTTTCCGTAGGTGAACCTGCGGAAG
GATCATTC
>XR_001160982 – Babesia microti strain RI 18S ribosomal RNA rRNA
AACCUGGUUGAUCCUGCCAGUAGUCAUAUGCUUGUCUUAAAGAUUAAGCCAUGCAUGUCUUAGUAUAAGCUUUUAUACAG
CGAAACUGCGAAUGGCUCAUUAAAACAGUUAUAGUUUAUUUGAUGUUCGUUUUACAUGGAUAACCGUGGUAAUUCUAGGG
CUAAUACAUGCUCGAGGCGCGUUUUCGCGUGGCGUUUAUUAGACUUUAACCAACCCUUCGGGUAAUCGGUGAUUCAUAAU
AAAUUAGCGAAUCGCAUGGCUUUGCCGGCGAUGUAUCAUUCAAGUUUCUGACCUAUCAGCUUUGGACGGUAGGGUAUUGG
CCUACCGGGGCGACGACGGGUGACGGGGAAUUGGGGUUCGAUUCCGGAGAGGGAGCCUGAGAAACGGCUACCACAUCUAA
GGAAGGCAGCAGGCGCGCAAAUUACCCAAUCCUGACACAGGGAGGUAGUGACAAGAAAUAACAAUACAGGGCUUAAAGUC
UUGUAAUUGGAAUGAUGGGAAUCUAAACCCUUCCCAGAGUAUCAAUUGGAGGGCAAGUCUGGUGCCAGCAGCCGCGGUAA
UUCCAGCUCCAAUAGCGUAUAUUAAAGUUGUUGCAGUUAAGAAGCUCGUAGUUGAAUUUCUGCCUUGUCAUUAAUCUCGC
UUCCGAGCGUUUUUUUAUUGACUUGGCAUCUUCUGGAUUUGGUGCCUUCGGGUACUAUUUUCCAGGAUUUACUUUGAGAA
AACUAGAGUGUUUCAAACAGGCAUUCGCCUUGAAUACUACAGCAUGGAAUAAUGAAGUAGGACUUUGGUUCUAUUUUGUU
GGUUAUUGAGCCAGAGUAAUGGUUAAUAGGAGCAGUUGGGGGCAUUCGUAUUUAACUGUCAGAGGUGAAAUUCUUAGAUU
UGUUAAAGACGAACUACUGCGAAAGCAUUUGCCAAGGAUGUUUUCAUUAAUCAAGAACGAAAGUUAGGGGAUCGAAGACG
AUCAGAUACCGUCGUAGUCCUAACCAUAAACUAUGCCGACUAGAGAUUGGAGGUCGUCAGUUUAAACGACUCCUUCAGCA
CCUUGAGAGAAAUCAAAGUCUUUGGGUUCUGGGGGGAGUAUGGUCGCAAGUCUGAAACUUAAAGGAAUUGACGGAAGGGC
ACCACCAGGCGUGGAGCCUGCGGCUUAAUUUGACUCAACACGGGAAACCUCACCAGGUCCAGACAUAGAGAGGAUUGACA
GAUUGAUAGCUCUUUCUUGAUUCUAUGGGUGGUGGUGCAUGGCCGUUCUUAGUUGGUGGAGUGAUUUGUCUGGUUAAUUC
CGUUAACGAACGAGACCUUAACCUGCUAAAUUAGGAUCUGGGACAAGCUUUGCUGUUCCAGUAUCGCUUCUUAGAGGGAC
UUUGCGUUCAUAAAACGCAAGGAAGUGUAAGGCAAUAACAGGUCUGUGAUGCCCUUAGAUGUCCUGGGCUGCACGCGCGC
UACACUGAUGCAUUCAACGAGUUUUUCCUUGGCCGUCGGGUCCGGGUAAUCUUACAGUAUGCAUCGUGAUGGGGAUAGAU
UAUUGCAAUUAUUAAUCUUGAACGAGGAAUGCCUAGUAGGCGCGAGUCAUCAGCUCGUGCCGACUACGUCCCUGCCCUUU
GUACACACCGCCCGUCGCUCCUACCGAUCGAGUGAUCCGGUGAAUUAUUCGGACCAAGAAACGUGGAUUCGUCCUUCGUU
UUUUGGAAAGUUUUGUGAACCUUAUCACUUAAAGGAAGGAGAAGUCGUAACAAGGUUUCCGUAGGUGAACCUGCGGAAGG
A
>AY048113 – Babesia sp. MO1 18S ribosomal RNA gene, complete sequence
AACCTGGTTGATCCTGCCAGTAGTCATATGCTTGTCTTAAAGATTAAGCCATGCATGTCTAAGTACAAACTTTTTACGGT
GAAACTGCGAATGGCTCATTACAACAGTTATAGTTTCTTTGGTATTCGTTTTCCATGGATAACCGTGCTAATTGTAGGGC
TAATACAAGTTCGAGGCCTTTTGGCGGCGTTTATTAGTTCTAAAACCATCCCTTTTGGTTTTCGGTGATTCATAATAAAC
TTGCGAATCGCAATTTTTTGCGATGGACCATTCAAGTTTCTGACCCATCAGCTTGACGGTAGGGTATTGGCCTACCGAGG
CAGCAACGGGTAACGGGGAATTAGGGTTCGATTCCGGAGAGGGAGCCTGAGAAACGGCTACCACATCCAAGGAAGGCAGC
AGGCGCGCAAATTACCCAATCCTGACACAGGGAGGTAGTGACAAGAAATAACAATACAGGGCAATTGTCTTGTAATTGGA
ATGATGGTGACCTAAACCCTCACCAGAGTAACAATTGGAGGGCAAGTCTGGTGCCAGCAGCCGCGGTAATTCCAGCTCCA
ATAGCGTATATTAAACTTGTTGCAGTTAAAAAGCTCGTAGTTGAATTTTTGCGTGGTGTTAATATTGACTGATGTCGAGA
TTGCACTTCGCTTTTGGGATTTATCCCTTTTTACTTTGAGAAAATTAGAGTGTTTCAAGCAGACTTTTGTCTTGAATACT
TCAGCATGGAATAATAGAGTAGGACTTTGGTTCTATTTTGTTGGTTTGTGAACCTTAGTAATGGTTAATAGGAACGGTTG
GGGGCATTCGTATTTAACAGTCAGAGGTGAAATTCTTAGATTTGTTAAAGACGAACTACTGCGAAAGCATTTGCCAAGGA
CGTTTTCATTAATCAAGAACGAAAGTTAGGGGATCGAAGACGATCAGATACCGTCGTAGTCCTAACCATAAACTATGCCG
ACTAGGGATTGGAGGTCGTCATTTTTCCGACTCCTTCAGCACCTTGAGAGAAATCAAAGTCTTTGGGTTCTGGGGGGAGT
ATGGTCGCAAGGCTGAAACTTAAAGGAATTGACGGAAGGGCACCACCAGGCGTGGAGCCTGCGGCTTAATTTGACTCAAC
ACGGGGAAACTCACCAGGTCCAGACAATGTTAGGATTGACAGATTGATAGCTCTTTCTTGATTCTTTGGGTGGTGGTGCA
TGGCCGTTCTTAGTTGGTGGAGTGATTTGTCTGGTTAATTCCGTTAACGAACGAGACCTTAACCTGCTAACTAGTGTCCG
TAAAAAGGTTCGTCCGTTACGGTTTGCTTCTTAGAGGGACTTTGCGGCTCTAAGCCGCAAGGAAGTTTAAGGCAATAACA
GGTCTGTGATGCCCTTAGATGTCCTGGGCTGCACGCGCGCTACACTGATGCATTCATCGAGTTTTATCCCTTCCCGAAAG
GGCTGGGTAATCTTTAGTATGCATCGTGACGGGGATTGATTTTTGCAATTCTAAATCATGAACGAGGAATGCCTAGTATG
CGCAAGTCATCAGCTTGTGCAGATTACGTCCCTGCCCTTTGTACACACCGCCCGTCGCTCCTACCGATCGAGTGATCCGG
TGAATTATTCGGACCGTGGCCTTTCCGATTCGTCGGTTTGGCCTAGGGAAGTCTTGTGAACCTTATCACTTAAAGGAAGG
AGAAGTCGTAACAAGGTTTCCGTAGGTGAACCTGCAGAAGGATCAAGC
>AF036588 – Brugia malayi small subunit ribosomal RNA gene, partial sequence
AAAGATTAAGCCATGCATGTCTAAGTTCAAATAAACCTATAATGGTGAAACCGCGAACGGCTCATTATAACAGCTATAAT
GTACTTGATGTTGATTATCTAACGTGGATAACTGTGGCAATTCTAGAGCTAATACATGCACCAAAGCTCCGAATTTTAAA
CGAGCGCATCTATTAGATTAAAACCAATCGGATTATTAATTTTAATCCGTTAATTGGTGACTCTGAATAGCTATGGCTGA
TCGCATGGTCTTGTACCGGCGACGTATCTATCAAGTGTCTGCCTTATCAACTTTCGATGGTAGTTTATGTGCCTACCATG
GTTGTAACGGGTAACGGAGAATAAGGGTTCGACTCCGGAGAGGGAGCCTGAGAAACGGCTACCACATCCAAGGAAGGCAG
CAGGCGCGCAAATTACCCACTCTCAGAATGAGGAGGTAGTGACGAAAAATAACGAGACCGTTCTCTTTGRGGCCGGTTAT
CGGAATGGGTACAATTTAAACCTGTTAACGAGGATCTATGAGAGGGCAAGTCTGGTGCCAGCAGCCGCGGTAATTCCAGC
TCTCAAAGTGTATATCGTCATTGCTGCGGTTAAAAAGCTCGTAGTTGGATCTGCGTCTCAGGACCTGGTCCATCCATTGG
ATGAGAACTAGGCTCCTAGGCTAATATTGCCAGTTTTTCCATACGTTACCTTAATTGGTTGCATATGGTGGCTAGCAAGT
TTACCTTGAAAAAATTAGAGTGCTCAATGCGGGCTAATGCCTGAATACTCGTGCATGGAATAATGAAATAGGATCTCGGT
TCTATTTTGTTGGTTTTCTGATCTGAGATAATGGTTAAGAGGGACGGACGGGGGCATTCGTATCGCTGCGTGAGAGGTGA
AATTCTTGGACCGTAGCGAGACGTACGACTGCGAAAGCATTTGCCAAGAATGTCTTCATTAATCAAGAACGAAAGTCAGA
GGTTCGAAGGCGATCAGATACCGCCCTAGTTCTGACCGTAAACGATACCAACTAGCGTTCCGTCGGCGGTAAATACGCCT
TGACGGGCAGCTTCCCGGAAACGAAAGTTTTTCGGTTCCGGGGGAAGTATGGTTGCAAAGCTGAAACTTAAAGAAATTGA
CGGAAGGGCACCACCAGGAGTGGAGCCTGCGGCTTAATTTGACTCAACACGGGAAAACTCACCTGGCCCGGACACCGTGA
GGATTGACAGATTGAGAGCTCTTTCATGATTCGGTGGTTGGTGGTGCATGGCCGTTCTTAGTTGGTGGAGTGATTTGTCT
GGTTTATTCCGATAACGAGCGAGACTCTAGCCTACTAAATAGTTACTGGATAATTTTATCGTCCAGACAACTTCTTAGAG

```
GGACAAGCGGTGTTTAGCCGCATGAAGTTGAGCAATAACAGGTCTGTGATGCCCTTAGATGTCCAGGGCTGCACGCGCGC
TACACTGGAGGAATCAGCGTGCTGTAACCATTACCGAAAGGTATTGGTAACCCCTTGAAAATCCTCCGTGATCGGGATCG
GGAATTGCAATTATTTCCCTTGAACGAGGAATTCCTAGTAAGTGTGAGTCATCAGCTCACGTTGATTACGTCCCTGCCCT
TTGTACACACCGCCCGTCGCTGCCCGGGACTGAGCCGTTTCGAGAAAAGCGGAGACTGCTGTTTCAATACCTTTTCAAGG
GTAGAGATTCTTTGGTGGAAACTGCTTTAATCGCAGTGGCTTGAACCGGGCAAAAGTCGTAACAAGGTTTCCGTAGGTGA
ACCTGCGGAAGGATCA
>CXZRR18S - Cytauxzoon felis 18S ribosomal RNA
AACCUGGUUGAUCCUGCCAGUAGUCAUAUGCUUGUCUUAAAGAUUAAGCCAUGCAUGUCUAAGUAUAAGCUUUUAUAUGG
UGAAACUGCGAAUGGCUCAUUAAAACAGNUAUAAUUUAUUUGAUAUUCGUUUCUACAUGGAUAACCGUGCUAAUUGUAGG
GCUAAUACAUGUUCGAGACCUAUUUUUAAUAGGUGGCGUUUAUUAGACCUUAAACCAUCCCGCUUCGGCGGUAUAUCGGU
GAUUCAUAAUAAAUAUGCGAAUCGCAUUGCUUUAUGCUGGCGAUGUAUCAUUCAAGUUUCUGACCUAUCAGCUUUGGACG
GUAGGGUAUUGGCCUACCGGGGCAGCGACGGGUAACGGGGAAUUAGGGUUCGAUUCCGGAGAGGGAGCCUGAGAAAUGGC
UACCACAUCUAAGGAAGGCAGCAGGCGCGUAAAUUACCCAAUCCUAACACAGGGAGGUAGUGACAAGAAAUAACAAUACG
AGGCUUAAAGUCUUGUAAUUGGAAUGACGGAAAUUUAAGCUCUUUCCGGAGUAUCAAUUGGAGGGCAAGUCUGGUGCCAG
CAGCCGCGGUAAUUCCAGCUCCAAUAGCGUAUAUUAAACUUGUUGCAGUUAAAAAGCUCGUAGUUGAAUUUCUGCUGCAU
CAUUUAUAUUCCUUAAUCGGUUUAUUUAUGUUGUGGCUUUUUCUGGUGAUUAUAUUUCGGUAUGAUUAUCCAGAUUGUUA
CUUUGAGAAAAUUAGAGUGCUUAAAGCAGGCUUUUGCCUUGAAUACUUUAGCAUGGAAUAACUAAGUAGGACUUUGGUUC
UAUUUUGUUGGUUUAAGAGCCAAAGUAAUGAUUAAUAGGAACAGUUGGGGGCAUUCGUAUUUAACUGUCAGAGGUGAAAU
UCUUAGAUUUGUUAAAGACGAACUACUGCGAAAGCAUUUGCCAAGGAUGUUUUCAUUAAUCAAGAACGAAAGUUAGGGGA
UCGAAGACGAUCAGAUACCGUCGUAGUCCUAACCAUAAACUAUGCCGACUAGAGAUUGGAGGUCGUCAGUUUGAACGACU
CCUUCAGCACCUUGAGAGAAAUCAAAGUCUUUGGGUUCUGGGGGGAGUAUGGUCGCAAGGCUGAAACUUAAAGGAAUUGA
CGGAAGGGCACCACCAGGCGUGGAGCCUGCGGCUUAAUUUGACUCAACACGGGAAAACUUACCAGGUCCAGACAGAGGAA
GGAUUGACAGAUUGAUAGCUCUUUCUUGAUUCUUUGGGUGGUGGUGCAUGGCCGUUCUUAGUUGGUGGAGUGAUUUGUCU
GGUUAAUUCCGUUAACGAACGAGACCUUAACCUGCUAAAUAGGAUCUGAGAAUAAACUUUAUGUUGUCUCAGCAUCGCUU
CUUAGAGGGACUUUGCGGUUAUAAAUCGCAAGGAAGUUUAAGGCAAUAACAGGUCUGUGAUGCCCUUAGAUGUCCUGGGC
UGCACGCGCGCUACACUGAUGCAUUCAUCGAGUAUAUCCUUGGCCGAGAGGCUUGGGUAAUCUUUAGUAUGCAUCGUGAU
GGGGAUUGAUUAUUGCAAUUAUUAAUCAUGAACGAGGAAUGCCUAGUAGACGCGAGUCAUCAGCUCGUGUCGAUUACGUC
CCUGCCCUUUGUACACACCGCCCGUCGCUCCUACCGANCGAGUGAUCCGGUGAAUUAUUCGGACUGUGGUGAAUCUAAUU
CGUUAGAUACGCCAUGGAAAGUUUUGUGAACCUUAUCACUUAAAGGAAGGAGAAGUCGUAACAAGGUUUCCGUAGGUGAA
CCUGCAGAAGGAUC
>GQ332359 - Leishmania infantum 18S ribosomal RNA gene, complete sequence; and internal transcribed
spacer 1, partial sequence
TAGTCATATGCTTGTTTCAAGGACTTAGCCATGCATGCCTCAGAATCACTGCATTTGCAGGAATCTGCGCATGGCTCATT
ACATCAGACGTAATCTGCCGCAAAAATCTTGCGGTTTCCGCAAAATTGGATAACTTGGCGAAACGCCAAGCTAATACATG
AACCAACCGGGTGTTCTCCACTCCAGACGGTGGGCAACCATCGTCGTGAGACGCCCAGCGAATGAATGACAGTAAAACCA
ATGCCTTCACTGGCAGTAACACCCAGCAGTGTTGACTCAATTCATTCCGTGCGAAAGCCGGCTTGTTCCGGCGTCTTTTG
ACGAACAACTGCCCTATCAGCTGGTGATGGCCGTGTAGTGGACTGCCATGGCGTTGACGGGAGCGGGGGATTAGGGTTCG
ATTCCGGAGAGGGAGCCTGAGAAATAGCTACCACTTCTACGGAGGGCAGCAGGCGCGCAAATTGCCCAATGTCAAAACAA
AACGATGAGGCAGCGAAAAGAAATAGAGTTGTCAGTCCATTTGGATTGTCATTTCAATGGGGGATATTTAAACCCATCCA
ATATCGAGTAACAATTGGAGGACAAGTCTGGTGCCAGCACCCGCGGTAATTCCAGCTCCAAAAGCGTATATTAATGCTGT
TGCTGTTAAAGGGTTCGTAGTTGAACTGTGGGCTGTGCAGGTTTGTTCCTGGTCGTCCCGTCCATGTCGGATTTGGTGAC
CCAGGCCCTTGCAGCCCGTGAACATTCAAAGAAACAAGAAACACGGGAGTGGTTCCTTTCCTGATTTACGCATGTCATGC
ATGCCAGGGGGCGTCCGTGATTTTTTACTGTGACTAAAGAAGCGTGACTAAAGCAGTCATTTGACTTGAATTAGAAAGCA
TGGGATAACAAAGGAGCAGCCTCTAGGCTACCGTTTCGGCTTTTGTTGGTTTTAAAGGTCTATTGGAGATTATGGAGCTG
TGCGACAAGTGCTTTCCCATCGCAACCTCGGTTCGGTGTGTGGCGCCTTTGAGGGGTTTAGTGCGTCCGGTACGAGCTCC
GGTTCGTCCGGCCGTAACGCCTTTTCAACTCACGGCCTCTAGGAATGAAGGAGGGTAGTTCGGGGGAGAACGTACTGGGG
CGTCAGAGGTGAAATTCTTAGACCGCACCAAGACGAACTACAGCGAAGGCATTCTTCAAGGATACCTTCCTCAATCAAGA
ACCAAAGTGTGGAGATCGAAGATGATTAGAGACCATTGTAGTCCACACTGCAAACGATGACACCCATGAATTGGGGATCT
TATGGGCCGGCCTGCGGCAGGGTTTACCCTGTGTCAGCACCGCGCCCGCTTTTACCACCTTACGTATCTTTTCTATTCGG
CCTTTACCGGCCACCCACGGGAATATCCTCAGCACGTTTTCTGTTTTTTCACGCGAAAGCTTTGAGGTTACAGTCTCAGG
GGGGAGTACGTTCGCAAGAGTGAAACTTAAAGAAATTGACGGAATGGCACCACAAGACGTGGAGCGTGCGGTTTAATTTG
ACTCAACACGGGGAACTTTACCAGATCCGGACAGGATGAGGATTGACAGATTGAGTGTTCTTTCTCGATTCCCTGAATGG
TGGTGCATGGCCGCTTTTGGTCGGTGGAGTGATTTGTTTGGTTGATTCCGTCAACGGACGAGATCCAAGCTGCCCAGTAG
AATTCAGAATTGCCCATAGGATAGCAAACTCATCGGCGGGTTTTACCCAACGGTGGGCCGCATTCGGTCGAATTCTTCTC
TGCGGGATTCCTTTGTAATTGCACAAGGTGAAATTTTGGGCAACAGCAGGTCTGTGATGCTCCTCAATGTTCTGGGCGAC
ACGCGCACTACAATGTCAGTGAGAACAAGAAAAACGACTTTTGTCGAACCTACTTGATCAAAAGAGTGGGGAAACCCCGG
AATCACATAGACTCACTTGGGACCGAGGATTGCAATTATTGGTCGCGCAACGAGGAATGTCTCGTAGGCGCAGCTCATCA
AACTGTGCCGATTACGTCCCTGCCATTTGTACACACCGCCCGTCGTTGTTTCCGATGATGGTGCAATACAGGTGATCGGA
CAGGCGGTGTTTTATCCGCCCGAAAGTTCACCGATATTTCTTCAATAGAGGAAGCAAAAGTCGTAACAAGGTAGCTGTAG
GTGAACCTGCAGCTGGATCATTTTCCGATG
>AB369994 - Cryptosporidium hominis gene for 18S ribosomal RNA, isolate: Ar108
TGAATTTACCCAAGCCTCGGAATTAATAGTTTTTTTGGGGTTTTAACAGATTGCCCACTTTTTGGCCTATTCTGGGGGGC
GGCATTTATATCATAAAATTTCTGTGGGGAAGAGAGACCACTCTGAGAAATTTCTCGGAAAACTCTTTCAAGGGGGTTGT
TTTTTTTTTGGAAAACCAATATAATTGGTGACTCATAATAACTCTACGGATCACAATTAATGTGACATATCATTCAAGTT
TCTGACCTATCAGCTTTAGACGGTAGGGTATTGGCCTACCGTGGCAATGACGGGTAACGGGGAATTAGGGTTCGATTCCG
GAGAGGGAGCCTGAAAAACGGCTACCACATCTAAGGAAGGCAGCAGGCGCGCAAATTACCCAATCCTAATACAGGGAGGT
AGTGACAAGAAATAACAATACAGGACTTTTTGGTTTTGTAATTGGAATGAGTTAAGTATAAACCCCTTTACAAGTATCAA
TTGGAGGGCAAGTCTGGTGCCAGCAGCCGCGGTAATTCCAGCTCCAATAGCGTATATTAAAGTTGTTGCAGTTAAAAAGC
TCGTAGTTGGATTTCTGTTAATAATTTATATAAAATATTTTGATGAATATTTATATAATATTAACATAATTCATATTACT
ATTTTTTTTTTTAGTATATGAAATTCTACTTTGAGAAAATTAGAGTGCTTAAAGCAGGCATATGCCTTGAATACTCCAGC
ATGGAATAATATTAAAGATTTTTATCTTTTTTATTGGTTCTAAGATAAGAATAATGATTAATAGGGACTGTTGGGGGCAT
TTGTATTTAACAGTCAGAGGTGAAATTCTTAGATTTGTTAAAGACAAACTAATGCGAAAGCATTTGCCAAGGATGTTTTC
ATTAATCAAGAACGAAAGTTAGGGGATCGAAGACGATCAGATACCGTCGAAGTCTTAACCATAAACTATGCCAACTAGAG
```

ATGAGTTGTCTCTATACTCTATA
>X07773 - Leishmania donovani gene for ribosomal RNA small subunit
GATCTGGTTGATTCTGCCAGTAGTCATATGCTTGTTTCAAGGACTTAGCCATGCATGCCTCAGAATCACTGCATTTGCAG
GAATCTGCGCATGGCTCATTACATCAGACGTAATCTGCCGCAAAAATCTTGCGGTTTCCGCAAAATTGGATAACTTGGCG
AAACGCCAAGCTAATACATGAACCAACCGGGTGTTCTCCACTCCAGACGGTGGGCAACCATCGTCGTGAGACGCCCAGCG
AATGAATGACAGTAAAACCAATGCCTTCACTGGCAGTAACACCCAGCAGTGTTGACTCAATTCATTCCGTGCGAAAGCCG
GCTTGTTCCGGCGTCTTTTGACGAACAACTGCCCTATCAGCTGGTGATGGCCGTGTAGTGGACTGCCATGGCGTTGACGG
GAGCGGGGGATTAGGGTTCGATTCCGGAGAGGGAGCCTGAGAAATAGCTACCACTTCTACGGAGGGCAGCAGGCGCGCAA
ATTGCCCAATGTCAAAACAAAACGATGAGGCAGCGAAAAGAAATAGAGTTGTCAGTCCATTTGGATTGTCATTTCAATGG
GGGATATTTAAACCCATCCAATATCGAGTAACAATTGGAGGACAAGTCTGGTGCCAGCACCCGCGGTAATTCCAGCTCCA
AAAGCGTATATTAATGCTGTTGCTGTTAAAGGGTTCGTAGTTGAACTGTGGGCTGTGCAGGTTTGTTCCTGGTCGTCCCG
TCCATGTCGGATTTGGTGACCCAGGCCCTTGCAGCCCGTGAACATTCAAAGAAACAAGAAACACGGGAGTGGTTCCTTTC
CTGATTTACGCATGTCATGCATGCCAGGGGGCGTCCGTGATTTTTTACTGTGACTAAAGAAGCGTGACTAAAGCAGTCAT
TTGACTTGAATTAGAAAGCATGGGATAACAAAGGAGCAGCCTCTAGGCTACCGTTTCGGCTTTTGTTGGTTTTAAAGGTC
TATTGGAGATTATGGAGCTGTGCGACAAGTGCTTTCCCATCGCAACCTCGGTTCGGTGTGTGGCGCCTTTGAGGGGTTTA
GTGCGTCCGGTACGAGCTCCGGTTCGTCCGGCCGTAACGCCTTTTCAACTCACGGCCTCTAGGAATGAAGGAGGGTAGTT
CGGGGGAGAACGTACTGGGGCGTCAGAGGTGAAATTCTTAGACCGCACCAAGACGAACTACAGCGAAGGCATTCTTCAAG
GATACCTTCCTCAATCAAGAACCAAAGTGTGGAGATCGAAGATGATTAGAGACCATTGTAGTCCACACTGCCAAACGATG
ACACCCATGAATTGGGGATCTTATGGGCCGGCCTGCGGCAGGGTTTACCCTGTGTCCAGCACCGCGCCCGCTTTTACCAA
CTTACGTATCTTTTCTATTCGGCCTTTACCGGCCACCCACGGGAATATCCTCAGCACGTTTTCTGTTTTTTCACGCGAAA
GCTTTGAGGTTACAGTCTCAGGGGGGAGTACGTTCGCAAGAGTGAAACTTAAAGAAATTGACGGAATGGCACCACAAGAC
GTGGAGCGTGCGGTTTAATTTGACTCAACACGGGGAACTTTACCAGATCCGGACAGGATGAGGATTGACAGATTGAGTGT
TCTTTCTCGATTCCCTGAATGGTGGTGCATGGCCGCTTTTGGTCGGTGGAGTGATTTGTTTGGTTGATTCCGTCAACGGA
CGAGATCCAAGCTGCCCAGTAGAATTCAGAATTGCCCATAGGATAGCAAACTCATCGGCGGGTTTTACCCAACGGTGGGC
CGCATTCGGTCGAATTCTTCTCTGCGGGATTCCTTTGTAATTGCACAAGGTGAAATTTTGGGCAACAGCAGGTCTGTGAT
GCTCCTCAATGTTCTGGGCGACACGCGCACTACAATGTCAGTGAGAACAAGAAAAACGACTTTTGTCGAACCTACTTGAT
CAAAAGAGTGGGGAAACCCCGGAATCACATAGACTCACTTGGGACCGAGGATTGCAATTATTGGTCGCGCAACGAGGAAT
GTCTCGTAGGCGCAGCTCATCAAACTGTGCCGATTACGTCCCTGCCATTTGTACACACCGCCCGTCGTTGTTTCCGATGA
TGGTGCAATACAGGTGATCGGACAGGCGGTGTTTTATCCGCCCGAAAGTTCACCGATATTTCTTCAATAGAGGAAGCAAA
AGTCGTAACAAGGTAGCTGTAGGTGAACCTGCAGCTGGATCATTT
>PFARGEA - P.falciparum 18S ribosomal RNA in asexual parasites
AACCTGGTTGATCTTGCCAGTAGTCATATGCTTGTCTCAAAGATTAAGCCATGCAAGTGAAAGTATATATATATTTTATA
TGTAGAAACTGCGAACGGCTCATTAAAACAGTTATAGTCTACTTGACATTTTTATTATAAGGATAACTACGGAAAAGCTG
TAGCTAATACTTGCTTTATTATCCTTGATTTTTATCTTTGGATAAGTATTTGTTAGGCCTTATAAGAAAAAAGTTATTAA
CTTAAGGAATTATAACAAAGAAGTAACACGTAATAAATTTATTTTATTTAGTGTGTATCAATCGAGTTTCTGACCTATCA
GCTTTTGATGTTAGGGTATTGGCCTAACATGGCTATGACGGGTAACGGGGAATTAGAGTTCGATTCCGGAGAGGGAGCCT
GAGAAATAGCTACCACATCTAAGGAAGGCAGCAGGCGCGTAAATTACCCAATTCTAAAGAAGAGAGGTAGTGACAAGAAA
TAACAATGCAAGGCCAATTTTTGGTTTTGTAATTGGAATGGTGGGAATTTAAAACCTTCCCAGAGTAACAATTGGAGGGC
AAGTCTGGTGCCAGCAGCCGCGGTAATTCCAGCTCCAATAGCGTATATTAAAATTGTTGCAGTTAAAACGCTCGTAGTTG
AATTTCAAAGAATCGATATTTTATTGTAACTATTCTAGGGGAACTATTTTAGCTTTTGGCTTTAATACGCTTCCTCTATT
ATTATGTTCTTTAAATAACAAAGATTCTTTTTAAAATCCCCACTTTTGCTTTTGCTTTTTTGGGGATTTTGTTACTTTGA
GTAAATTAGAGTGTTCAAAGCAAACAGTTAAAGCATTTACTGTGTTTGAATACTATAGCATGGAATAACAAAATTGAACA
AGCTAAAATTTTTTGTTCTTTTTTCTTATTTTGGCTTAGTTACGATTAATAGGAGTAGCTTGGGGACATTCGTATTCAGA
TGTCAGAGGTGAAATTCTTAGATTTTCTGGAGACGAACAACTGCGAAAGCATTTGTCTAAAATACTTCCATTAATCAAGA
ACGAAAGTTAAGGGAGTGAAGACGATCAGATACCGTCGTAATCTTAACCATAAACTATGCCGACTAGGTGTTGGATGAAA
GTGTTAAAAATAAAAGTCATCTTTCGAGGTGACTTTTAGATTGCTTCCTTCAGTACCTTATGAGAAATCAAAGTCTTTGG
GTTCTGGGGCGAGTATTCGCGCAAGCGAGAAAGTTAAAAGAATTGACGGAAGGGCACCACCAGGCGTGGAGCTTGCGGCT
TAATTTGACTCAACACGGGGAAACTCACTAGTTTAAGACAAGAGTAGGATTGACAGATTAATAGCTCTTTCTTGATTTCT
TGGATGGTGATGCATGGCCGTTTTTAGTTCGTGAATATGATTTGTCTGGTTAATTCCGATAACGAACGAGATCTTAACCT
GCTAATTAGCGGCGAGTACACTATATTCTTATTTGAAATTGAACATAGGTAACTATACATTTATTCAGTAATCAAATTAG
GATATTTTTATTAAAATATCCTTTTCCCTGTTCTACTAATAAATTGTTTTTTACTCTATTTCTCTCTTCTTTTAAGAATG
TACTTGCTTGATTGAAAAGCTTCTTAGAGGAACATTGTGTGTCTAACACAAGGAAGTTTAAGGCAACAACAGGTCTGTGA
TGTCCTTAGATGAACTAGGCTGCACGCGTGCTACACTGATATATATAACGAGTTTTTAAAAATATGCTTATATTTGTATC
TTTGATGCTTATATTTTGCATACTTTTCCTCCGCCGAAAGGCGTAGGTAATCTTTATCAATATATATCGTGATGGGGATA
GATTATTGCAATTATTAATCTTGAACGAGGAATGCCTAGTAAGCATGATTCATCAGATTGTGCTGACTACGTCCCTGCCC
TTTGTACACACCGCCCGTCGCTCCTACCGATTGAAAGATATGATGAATTGTTTGGACAAGAAAAATTGAATTATATTCTT
TTTTTTCTGGAAAAACCGTAAATCCTATCTTTTAAAGGAAGGAGAAGTCGTAACAAGGTTTCCGTAGGTGAACCTGCGGA
AGGATCATTA
>PFARRSSU - Plasmodium knowlesi small subunit ribosomal RNA sequence
AACCUGGUUGAUCUUGCCAGUAGUCAUAUGCUUGUCUCAAAGAUUAAGCCAUGCAAGUGAAAGUAUAUGCAUAUUUUAUA
UGUAGAAACUGCGAACGGCUCAUUAAAACAGUUAUAAUCUACUUGACAUUUUCUAUAUAAGGAUAACUACGGAAAAGCUG
UAGCUAAUACUUGCUUUAGCACUCUUGAUUUCUUUCUUGAGUGUGUACUUGUUAAGCCUUAUAAGAAAAGAGUUAUUAAC
UUAAGGAAUUAUAACAAAGAAGUAACACGUAAUGGAUUCUUCCAUUUUUAGUGUGUAUCAAUCGAGUUUCUGACCUAUCA
GCUUUUGAUGUUAGGGUAUUGGCCUAACAUGGCUAUGACGGGUAACGGGGAAUUAGAGUUCGAUUCCGGAGAGGGAGCCU
GAGAAAUAGCUACCACAUCUAAGGAAGGCAGCAGGCGCGUAAAUUACCCAAUUCUAAAGAAGAGAGGUAGUGACAAGAAA
UAACAAUACAAGGCCAAUCUGGCUUUGUAAUUGGAAUGAUGGGAAUUUAAAACCUUCCCAAAAUUCAAUUGGAGGGCAAG
UCUGGUGCCAGCAGCCGCGGUAAUUCCAGCUCCAAUAGCGUAUAUUAAAAUUGUUGCAGUUAAAACGCUCGUAGUUGAAU
UUCAAAGAAUCGAUAUUUUAAGCAACGCUUCUAGCUAUAAAUCCACAUAACUGAUGCCUCCGCGUAUCGACUUUGUGCGC
AUUUUAGCUAUUAUGUGUUCUUUUAAUUAAAAUGAUUCUUUUUAAGAUUCAUCUAUUAAAAAUUCGCUUCGGCAUAAUUU
UUUUGAUGAUCUUGUUACUUUGAGUAAAUUAGAGUGUUCAAAGCAAACAGAUAUAGCAUUUGUGCGUUUGAAUACUACAG
CAUGGAAUAACAAAAUUGAACAAGUCAGAAUUUUUUUGUUCUUUUUUCUUAUUUUGGCUUAGUUACGAUUAAUAGGAGUA
GCUUGGGGGCAUUUGUAUUCAGAUGUCAGAGGUGAAAUUCUUAGAUUUUCUGGAGACAAACAACUGCGAAAGCAUUUGCC
UAAAAUACUUCCAUUAAUCAAGAACGAAAGUUAAGGGAGUGAAGACGAUCAGAUACCGUCGUAAUCUUAACCAUAAACUA

UGCCGACUAGGCUUUGGAUGAAAGAUUUUAAAAUAAGAGUUUUUCUUUUCUCUCCGGAGAUUAGAACUCUUAGAUUGCUU
CCUUCAGUGCCUUAUGAGAAAUCAAAGUCUUUGGGUUCUGGGGCGAGUAUUCGCGCAAGCGAGAAAGUUAAAAGAAUUGA
CGGAAGGGCACCACCAGGCGUGGAGCUUGCGGCUUAAUUUGACUCAACACGGGAAAACUCACUAGUUUAAGACAAGAGUA
GGAUUGACAGAUUAAUAGCUCUUUCUUGAUUUCUUGGAUGGUGAUGCAUGGCCGUUUUUAGUUCGUGAAUAUGAUUUGUC
UGGUUAAUUCCGAUAACGAACGAGAUCUUAACCUGCUAAUUAGCGGCAAAUACGAUAUAUUCUUAUGUAGAAUUGAAUAU
AGUGGAUUUGUUAGAUUUUGAAGAAAAUAUUGGAAUUACGUUAAAUGUGAUUCCUUUCCCUUUUCUACUUAAUUUACAUU
UCCAUCUAUUUCUUUUUUGCGUAUGAAUGUAUUUGCUUGAUUGUAAAGCUUCUUAGAGGAACGAUGUGUGUCUAACACAA
GGAAGUUUAAGGCAACAACAGGUCUGUGAUGUCCUUAGAUGAACUAGGCUGCACGCGUGCUACACUGAUAUGUAUAACGA
GUUACUAAAAUUACGAUUUUAGCUGCUUGCAGUUUAUUUUCGUACUUUUCCUCCACUGAAAAGUGUAGGUAAUCUUUAUC
AAUACAUAUCGUGAUGGGGAUAGAUUAUUGCAAUUAUUAAUCUUGAACGAGGAAUGCCUAGUAAGCAUGAUUCAUCAGAU
UGUGCUGACUACGUCCCUGCCCUUUGUACACACCGCCCGUCGCUCCUACCGAUUGAAAGAUAUGAUGAAUUGUUUGGACA
AGAAAAAAUUGGAUUAUAUCUUUUUUUUUGGAAAAACCGUAAAUCCUAUCUUUUAAAGGAAGGAGAAGUCGUAACAAGGU
UUCCGUAGGUGAAUUC
>PFARGBAB - P.malariae small subunit ribosomal RNA gene
ATATATATATATACTGTTTGTGTTTAATTGTAGTAGAAATACTACGATTATTTTGCAATATGTGCGTTATATATTTATTG
TATTTAAGCCATTTTTTATAACATTTAAGTTATGATATTATTATTGATTTTAGTGAAATTATGTTCTGTTTTTATAATAT
TATTATATAAGTAACCTGGTTGATCTTGCCAGTAGTCATATGCTTGTCTCAAAGATTAAGCCATGCAAGTGAAAGTATAT
GCATATTTTATATGTAGAAACTGCGAACGGCTCATTAAAACAGTTATAGTCTACTTGACATTTTTTTTATAAGGATAACT
ACGGAAAAGCTGTAGCTAATACTTGCTTTAATACTCTTAATTCTTATGTTTTTTGAGTATGTATTTGTTAAGCCTTATA
AGAGAAAAGTATATTAACTTAAGGATTATAACAAAGAAGTAACACATAATAAATTTCGAATATTTAGTGTGTATCAATCG
AGTTTCTGACCTATCAGCTTTTGATGTTAGGGTATTGGCCTAACATGGCTATGACGGGTAACGGGGAATTAGAGTTCGAT
TCCGGAGAGGGAGCCTGAGAAATAGCTACCACATCTAAGGAAGGCAGCAGGCGCGTAAATTACCCAATTCTAAAGAAGAG
AGGTAGTGACAAGAAATAACAATGCAAGGCCAAATTTTGGTTTTGCAATTGGAATGATGGGAATTTAAAACCTTCCCAGA
AGGCAATTGGAGGGCAAGTCTGGTGCCAGCAGCCGCGGTAATTCCAGCTCCAATAGCGTATATTAAAATTGTTGCAGTTA
AAACGCTCGTAGTTGAATTTCAAGGAATCAATATTTTAAGTAATGCTTTGTATATTTATAACATAGTTGTACGTTAAGAA
TAACCGCCAAGGCTTATATTTTTTCTGTTACATTTTGTTTTATTAATATATATATGCGTTCTTATTATAAAAATGATTCT
TTTTAAAATTCTTTTGTATAATTTTTTATGCATGGGAATTTTGTTACTTTGAGTAAATTAGAGTGTTCAAAGCAAACAGT
TAAAACAGTTTCTGTGTTTGAATACTACAGCATGGAATAACAAAATTGAACAAGTCAGAAGTTTGTTCTTTTTTTCTTATT
TTGGCTTAGTTACGATTAATAGGAGTAGCTTGGGGGCATTTGTATTCAGATGTCAGAGGTGAAATTCTTAGATTTTCTGG
AGACAAGCAACTGCGAAAGCATATGCCTAAAATACTTCCATTAATCAAGAACGAAAGTTAAGGGAGTGAAGACGATCAGA
TACCGTCGTAATCTTAACCATAAACTATGCCGACTAGGTGTTGGATGATAGTGTAAAAAATAAAAGAGACATTCTTATAT
ATGAGTGTTTCTTTTAGATAGCTTCCTTCAGTACCTTATGAGAAATCAAAGTCTTTGGGTTCTGGGGCGAGTATTCGCGC
AAGCGAGAAAGTTAAAAGAACCGACGGAAGGGGACACAGGCGTGGAGCTTGCGGCTTAATTTGACTCAACACGGGGAAAC
TCACTAGTTTAAGACAAGAGTAGGATTGACAGATTAATAGCTCTTTCTTGATTTCTTGGATGGTGATGCATGGCCGTTTT
TAGTTCGTGAATATGATTTGTCTGGTTAATTCCGATAACGAACGAGATCTTAACCTGCTAATTAGCGGTAATACACTATA
TTCTTAAGTGAAATTAGAATATAGATAAATTGTGCTAATTTTGATTAAAATATTAGAATGTTTTTTTAATAAAAACGTT
CTTTTCCCTTTTTTTCTTAATTATGCATATTTATTCTTTTTCTTTTTTCGCATAAGAATGTATTTGCTTAATGTAAGCTT
CTTAGAGGAACGATGTGTGTCTAACACAAGGAAGTTTAAGGCAACAACAGGTCTGTGATGTCCTTAGATGAACTAGGCTG
CACGCGTGCTACACTGATATGTATAACGAGTATTTAAAAATATATATCTTGTTATGTTATACGTATTTCTATATGTATGC
ATACAAAGAATATATAGTTTGCTCACTGAAAGTGTAGGTAATCTTTATCAATATATATCGTGATGGGGATAGATTATTGC
AATTATTAATCTTGAACGAGGAATGCCTAGTAAGCATGATTCATCAGATTGTGCTGACTACGTCCCTGCCCTTTGTACAC
ACCGCCCGTCGCTCCTACCGATTGAAAGATATGATGAATTGTTTGGACAAGAAAAAAGGTTTTTATTCTTTTTTCTGGAA
AAATCGTAAATCCTATCTTTTAAAGGAAGGAGAAAGTCGTAACAAGGTTTCCGTCGGTGAACCTGCGGAAGGATCATTA
>KF696369 - Plasmodium ovale curtisi clone DC-1 18S ribosomal RNA gene, complete sequence
AACCTGGTTGATCTTGCCAGTAGTCATATGCTTGTCTCAAAGATTAAGCCATGCAAGTGAAAGTATATGCATATTTTATA
TGTAGAAACTGCGAACGGCTCATTAAAACAGTTATAATCTACTTGAAATTTCTACCTTACAAGGATAACTACGGAAAAGC
TGTAGCTAATACTTGCTTTAATGCGTTTGAATCCTTTTGGATTCCCGCGTATGTACTTGTTAAGCCTTTAAGAAAGAAGT
TTATAACTTAAGGAATTATAACAAAGAAGTAACACATAATAAGGTTGCCTTATTTAGTGTGTATCAATCGAGTTTCTGAC
CTATCAGCTTTTGATGTTAGGGTATTGGCCTAACATGGCTATGACGGGTAACGGGGAATTAGAGTTCGATTCCGGAGAGG
GAGCCTGAGAAATAGCTACCACATCTAAGGAAGGCAGCAGGCGCGTAAATTACCCAATTCTAAAGAAGAGAGGTAGTGAC
AAGAAATAACAATACAAGGCCATTTCATGGTTTTGTAATTGGAATGATGGGAATTTAAAACCTTCCCAAAATTCAATTGG
AGGGCAAGTCTGGTGCCAGCAGCCGCGGTAATTCCAGCTCCAATAGCGTATATTAAAATTGTTGCAGTTAAAACGCTCGT
AGTTGAATTTCAAAGAATCAGTATTTTAAGTAATACTTTTGCTATAAGATGCTTAGACAATACAACGTATCTGTTCTTTG
CATTCCTTATGCAAAATGTGTTCTTATTATAAAAAGGATTCTTTTTAAAATCTCTTTTGCTATTTTTTAGTATTGGAGAT
TTTGTTACTTTGAGTAAATTAGAGTGTTCAAAGCAAACAGTTAAAGCATTTTTACTGCGTTTGAATACTACAGCATGGAA
TAACAAAATTGAACAAGTCAGAATTCTGTTCTTTTTTCTTATTTTGGCTTAGTTACGATTAATAGGAGTAGCTTGGAGGC
ATTTGTATTCAGATGTCAGAGGTGAAATTCTTAGATTTTCTGGAGACAAACAACTGCGAAAGCATTTGCCTAAAATACTT
CCATTAATCAAGAACGAAAGTTAAGGGAGTGAAGACGATCAGATACCGTCGTAATCTTAACCATAAACTATGCCGACTAG
GTTTTGGATGAAAGATTTTTAAATAAGAAAATTCCTTTCGGGGAAATTTCTTAGATTGCTTCCTTCAGTACCTTATGAGA
AATCAAAGTCTTTGGGTTCTGGGGCGAGTATTCGCGCAAGCGAGAAAGTTAAAAGAATTGACGGAAGGGCACCACCAGGC
GTGGAGCTTGCGGCTTAATTTGACTCAACACGGGGAAACTCACTAGTTTAAGACAAGAGTAGGATTGACAGATTAATAGC
TCTTTCTTGATTTCTTGGATGGTGATGCATGGCCGTTTTTAGTTCGTGAATATGATTTGTCTGGTTAATTCCGATAACGA
ACGAGATCTTAACCTGCTAATTAGCGGCGAATACGTGATATTCTTATGCGAAATTGAATATAGCTGAATTTGCTTATTTT
GAAGAATACATTAGGATACAATTAATGTGTCCTTTTCCCTATTCTTCTTAATTCGCAATTCATGCTGTTTCTCTTTTGCA
TTAGAATGTATTCGTTTGATTGTAAAGCTTCTTAGAGGAACGATGTGTGTCTAACACAAGGAAGTTTAAGGCAACAACAG
GTCTGTGATGTCCTTAGATGAACTAGGCTGCACGCGTGCTACACTGATATGTAAAACGAGTTACTAAAAATATACTTTTG
TTAGGGTATTTTATATACTCTTTCAAATTGTATAGTTTCCTCCACTGAAAAGTGTAGGTAATCTTTATCAGTACATATC
GTGATGGGGATAGATTATTGCAATTATTAATCTTGAACGAGGAATGCCTAGTAAGCATGATTCATCAGATTGTGCTGACT
ACGTCCCTGCCCTTTGTACACACCGCCCGTCGCTCCTACCGATTGAAAGATATGATGAATTGTTTGGACAAGAAAAGAAA
GAATTTATATTCTTTTTTTTCTGGAAAAACCGTAAATCCTATCTTTTAAAGGAAGGAGAAGTCGTAACAAGGTTTCCGTA
GGTGAACCTGCGGAAGGATCATTA
>X13926 - Plasmodium vivax small subunit (SSU) rRNA gene
ATCTGTTCAATTTTATGCTATATTGATTGAGTGTTGTTTATGAAAGGCATCATGGTGGGGTTAAGAAGGACCTTGCAATG

AAAGACTAGCATTTGTAGGTATGTTACTCTTAACTTTTAGTAGGTGCAATAATTCCTATCATAAAGTTATATGGAAAGAT
AACGAAATGGTAAGGAAAATGACAAATAAAACGTCAGCTGTAGTTATTATACGTTACTATGCGAAAGGTAGTAACATGTT
AGCGAACAACGAAGTGTGTTTATACAGCAAATGGAGTGTAGTGTTTAAAGTGTCTACAAATGTGCACTTCTCACTTCCCC
ACTGCCGTATATAGTAACCTGGTTGATCTTGCCAGTAGTCATATGCTTGTCTCAAAGATTAAGCCATGCAAGTGAAAGTA
TATGCATATTTTATATGTAGGAACTCGCAACGGCTCATTAAAACAGTTATAATCTACTTGACATTTTTTCTATAAGGATA
ACTACGGAAAAGCTGTAGCTAATACTTGCTTTAGCACTCTTGATTCATTTCTTGAGTGTGTACTTGTTAAGCCTTTTAAG
AAAAAAGTTATTAACTTAAGGAATTATAACAAAGAAGCGACACGTAATGGATCCGTCCATTTTTAGTGTGTATCAATCGA
GTTCTGACCTATCAGCTTTTGATGTTAGGGTATTGGCCTAACATGGCTATGACGGGTAACGGGGAATTAGAGTTCGATTC
CGGAGAGGGAGCCTGAGAAATAGCTACCACATCTAAGGAAGGCAGCAGGCGCGTAAATTACCCAATTCTAAAGAAGAGAG
GTAGTGACAAGAAATAACAATACAAGGCCAATCTGGCTTTGTAATTGGAATGATGGGAATTTAAAACCTTCCCAAAACTC
AATTGGAGGGCAAGTCTGGTGCCAGCAGCCGCGGTAATTCCAGCTCCAATAGCGTATATTAAAATTGTTGCAGTTAAAAC
GCTCGTAGTTGAATTTCAAAGAATCGATATTTTAAGCAACGCTTCTAGCTTAATCCACATAACTGATACTTCGTATCGAC
TTTGTGCGCATTTTGCTATTATGTGTTCTTTTAATTAAAATGATTCTTTTTAAGGACTTTCTTTGCTTCGGCTTGGAAGT
CCTTGTTACTTTGAGTAAATTAGAGTGTTCAAAGCAAACAGATATAGCATTGCGCCGTTTGAATACTACAGCATGGAATA
ACAAATTGAACAAGTCAGAATTTTTCTTCTTTTTTCTTATTTTGGCTTAGTTACGATTAATAGGAGTAGCTTGGGGGCAT
TTGTATTCAGATGTCAGAGGTGAAATTCTTAGATTTTCTGGAGACAAACAACTGCGAAAGCATTTGCCTAAAATACTTCC
ATTAATCAAGAACGAAAGTTAAGGGAGTGAAGACGATCAGATACCGTCGTAATCTTAACCATAAACTATGCCGACTAGGC
TTTGGATGAAAGATTTTAAAATAAGAATTTTCTCTTCGGAGTTTATTCTTAGATTGCTTCCTTCAGTGCCTTATGAGAAA
TCAAAGTCTTTGGGTTCTGGGGCGAGTATTCGCGCAAGCGAGAAAGTTAAAAGAATTCGGAAGGGCACCACCAGGCGTGG
AGCTTGCGGCTTAATTTGACTCAACACGGGAAAACTCACTAGTTTAAGACAAGAGTAGGATTGACAGATTAATAGCTCTT
TCTTGATTTCTTGGATGGTGATGCATGGCCGTTTTTAGTTCGTGAATATGATTTGTCTGGTTAATTCCGATAACGAACGA
GATCTTAACCTGCTAATTAGCGGCAAATACGATATATTCTTACGTGGGACTGAATTCGGTTGATTTGCTTACTTTGAAGA
AAATATTGGGAAACGTAACAGTTTCCCTTTCCCTTTTCTACTTAGTTCGCTTTTCATACTGTTTCTTTTCGCGTAAGAAT
GTATTTGCTTGATTGTAAAGCTT
>AJ009141 – Trypanosoma brucei gambiense 18S rRNA gene, isolate Tsuaa (clone G)
GTCATATGCTTGTTTCAAGGACTTAGCCATGCATGCCTCAGAATCACTGCATTGCAGGAATCTGCGCATGGCTCATTACA
TCAGACGTAATCTGCCGCCAAAATTTTGCGGTCTCCGCATTACTGGATAACTTGGCGAAACGCCAAGCTAATACATGAAC
CAATCGGACGCTCTCTTTTTCTATGTCGCGGCTTGTGTTTACGCACTTGTCGTGGCGATGGGACGTCCAGCGAATGAATG
AAATTAGAACCAACGCCTCCACCCGGGGGCAGTAACACTCAGACGTGTTGACTCAATTCATTCCGTGCGAAAGCCGAGCC
TTGTTGCTCGGCGTCTACTGACGAACAACTGCCCTATCAGCCAGTGATGGCCGTGTAGTGGACTGCCATGGCGTTGACGG
GAGCGGGGGATTAGGGTTCGATTCCGGAGAGGGAGCCTGAGAAATAGCTACCACTTCTACGGAGGGCAGCAGGCGCGCAA
ATTGCCCAATGTCGAAAAAATACGATGAGGCAGCGAAAAGAAATAGAGCCGACCGTGCCCTAGTGCATGGTTGTTTTCAA
TGGGGGATACTCAAACCCATCCAATATCGAGTAACAATTGGAGGACAAGTCTGGTGCCAGCACCCGCGGTAATTCCAGCT
CCAAAAGCGTATATTAATGCTGTTGCTGTTAAAGGGTTCGTAGTTGAACTGTGGGCCACGTAGTTTTGTGCCGTGCCAGT
CCCGTCCACCTCGGACGTGTTTTGACCCACGCCCTCGTGGCCCGTGAACACACTCAGATACAAGAAACACGGGAGCGGTT
CCTCCTCACTTTCACGCATGTCATGCATGCGAGGGGGCGTCCGTGAATTTTACTGTGACCAAAAAAGTGCGACCAAAGCA
GTCTGCCGACTTGAATTACAAAGCATGGGATAACGAAGCATCAGCCCTGGGGCCACCGTTTCGGCTTTTGTTGGTTTTAG
AAGTCCTTGGGAGATTATGGGGCCGCGTGCCTTGGGTCGGTGTTTCGTGTCTCATTTTTGTGGCGCGCACATTCGGCTCT
TCGTGATGTTTTTTTACATTCATTGCGACGCGCGGCTTCCAGGAATGAAGGAGGGTAGTTCGGGGGAGAACGTACTGGTG
CGTCAGAGGTGAAATTCTTAGACCGCACCAAGACGAACTACAGCGAAGGCATTCTTCAAGGATACCTTCCTCAATCAAGA
ACCAAAGTGTGGGGATCAAAGATGATTAGAGACCATTGTAGTCCACACTGCAAACCATGACACCCATGAATTGGGGAACA
TCATTGGGTGCCCGTGTGGCGGCCTTTTGTGCCGACCCTCGGCCCCAATTTATTTATCAATTTACGTGCCTATTCTATCA
CCCCCGGTTCCCTCTTTTGAGGTTCTTCCGGGGTTTTTTACGGGAATATCCTCAGCACGTTTCTTACTTCTTCACGCGAA
AGCTTGGAGGTTACAGTCTCAGGGGGGAGTACGTTCGCAAGAGTGAAACTTAAAGAAATTGACGGAATGGCACCACAAGA
CGTGGAGCGTGCGGTTTAATTTGACTCAACACGGGGAACTTTACCAGATCCGGACAGGGTGAGGATTGACAGATGGAGTG
TTCTTTCTCGATCCCCTGAATGGTGGTGCATGGCCGCTTTTGGTCGGTGGAGTGATTTGTTTGGTTGATTCCGTCAACGG
ACGAGATCCAAGCTGCCCAGTAGGTGCCGGGATTGTCCACACAGGACAGCAGTCCCTCCGGCGGGGATTTTTTCCCCAAC
GGTGGTCGTCATCCTTCTTTTTACAGGCCCCTTCTCTGCGGGATTCCTTGCTTTTCGCGCAAGGTGAGATTTTGGGCAAC
AGCAGGTCTGTGATGCTCCTCAATGTTCTGGGCGACACGCGCACTACAATGTCAGTGAGAACAAGAGTCCGAGCGGCACT
TCACAATGTCGCTCCCGCTTGATCAAAAGAGCGGGGAAACCACGGAATCACGTAGACCCACTTGGGACCGAGTATTGCAA
TTATTGGTCGCGCAACGAGGAATGTCTCGTAGGCGCAGCTCATCAAACTGTGCCGATTACGTCCCTGCCATTTGTACACA
CCGCCCGTCGTTGTTTCCGATGATGGTGCAATACAGGTGATCGGACCGTCGCTCGTCTCGGGCGACCGAAAGTTCACCGA
TATTGCTTCAATAGAGGAAGCAAAAGTC
>AF239980 – Trypanosoma cruzi strain Colombiana 18S ribosomal RNA gene, complete sequence
CATATGCTTGTTTCAAGGACTTAGCCATGCATGCCTCAGAATCACTGCATTGCAGGAATCTGCGCATGGCTCATTACATC
AGACGTAATCTGCCGCAAAAATCTTGCGGTCTCCGCAAAATTGGATAACTTGGCGAAACGCCAAGCTAATACATGAACCA
ACCGGATGTTCTCTGTTCCGGCGGCAGGGCAACCTGCTGCCATGGGACGTCCAGCGAATGAATGAAAGTAAAACCAATGC
CTTCACCGGGCAGTAACACTCAGAAGTGTTGATTCAATTCATTCCGTGCGAAAGCCGGGTTTTTTATCCGGCGTCTTTTG
ACGAACAACTGCCCTATCAGCCAGCGATGGCCGTGTAGTGGACTGCCATGGCGTTGACGGGAGCGGGGGATTAGGGTTCG
ATTCCGGAGAGGGAGCCTGAGAAATAGCTACCACTTCTACGGAGGGCAGCAGGCGCGCAAATTGCCCAATGTCAAAAAAA
AAAGATGAGGCAGCGAAAAGAAATAGAGCCGACAGTGCTTTTGCATTGTCGTTTTCAATGGGGGATATTTAAACCCATCC
AAAATCGAGTAACAATTGGAGGACAAGTCTGGTGCCAGCACCCGCGGTAATTCCAGCTCCAAAAGCGTATATTAATGCTG
TTGCTGTTAAAGGGTTCGTAGTTGAATTGAGGGCCTCTAAGGCGCAATGGTTTAGTCCCATCCACTTCGGATTGGTGACC
CATGCCCTTGTGGTCCGTGAACAGACATATTCAGAAACAAAAAACACGGGAGTGGTACCTTTTCTGATTATCGCATGTCA
TGCATGCCAGAGGGCGCCCGTGATTTTTTACTGTGACTAAAAAAGTGTGACCAAAGCAGTCATTCGACTTGAATTAGAAA
GCATGGGATAACAAAGGAGCAGCCTCTGGGCCACCGTTTCGGCTTTTGTTGGTTTTAAAAGTCCATTGGAGATTATGGGG
CAGTGTGACAAGCGGCTGGGTGGTTATTCCACACACACACGCACACTCCTTTTTTGGATGTTGTGTCTTGTGTGTGGCAC
TCGTCGCCTTTGTGGGAAATCCGTGTGGCACTTGTTTGGTGTTGTTGGCAGACTTCGGTCTTGCCTTCACACACGTTTCA
CATGTGTCATGCCTTCCCTCAACTCACGGCATCCAGGAATGAAGGAGGGTAGTTCGGGGGAGAACGTACTGGTGCGTCAG
AGGTGAAATTCTTAGACCGCACCAAGACGAACTACAGCGAAGGCATTCTTCAAGGATACCTTCCTCAATCAAGAACCAAA
GTGTGGGGATCGAAGATGATTAGAGACCATTGTAGTCCACACTGCAAACGATGACACCCATGAATTGGGGAGTTTTTGGT
CGTAGGCGTGGTCGGGCTTGATTATATTATTTTTCATCCCGTTCCTCGTCTCGCCAATGAATATATTAAATTTACGTGCA
TATTCTTTTTGGTCTTCGTTTTTTTTACGGCGAGGACCTTTAACGGGAATATCCTCAGCACGTTATCTGACTTCTTCACG

```
CGAAAGCTTTGAGGTTACAGTCTCAGGGGGGAGTACGTTCGCAAGAGTGAAACTTAAAGAAATTGACGGAATGGCACCAC
AAGACGTGGAGCGTGCGGTTTAATTTGACTCAACACGGGGAACTTTACCAGATCCGGACAGGGTGAGGATTGACAGATTG
AGTGTTCTTTCTCGATCCCCTGAATGGTGGTGCATGGCCGCTTTTGGTCGGTGGAGTGATTTGTTTGGTTGATTCCGTCA
ACGGACGAGATCCAAGCTGCCCAGTAGGATTCAGAATTGCCCATAGGATAGCAATCCCTTCCGCGGGTTTTACCCAAGGG
GGGGCGGTATTCGCTTGTATCCTTCTCTGCGGGATTCCTTGTTTTGCGCAAGGTGAGATTTTGGGCAACAGCAGGTCTGT
GATGCTCCTCAATGTTCTGGGCGACACGCGCACTACAATGTCAGTGAGAACAAGAAAAACGACTCTTGTCGGACCTACTT
GATCAAAAGAGTGGGAAAACCCCGGAATCACGTAGACCCACTTGGGACCGAGTATTGCAATTATTGGTCGCGCAACGAGG
AATGTCTCGTAGGCGCAGCTCATCAAACTGTGCCGATTACGTCCCTGCCATTTGTACACACCGCCCGTCGTTGTTTCCGA
TGATGGTGCAATACAGGTGATCGGACAGTCGAGTGCTTCACTTGACCGAAAGTTCACCGATATTTCTTCAATAGAGGAAG
CAAAAG
>AF227234 - Wuchereria bancrofti 18S small subunit ribosomal RNA gene, partial sequence
AGCCATGCATGTCTAAGTTCAAATAAACCTATAATGGTGAAACCGCGAACGGCTCATTATAACAGCTATAATGTACTTGA
TGTTGATTATCCAACGTGGATAACTGTGGCAATTCTAGAGCTAATACATGCACCAAAGCTCCGATTTTTAGACGAGCGCA
TCTATTAGATTAAAACCAATCGGATTATTAATTAACCCGTTAATTGGTGACTCTGAATAGCTATGGCTGATCGCATGGTC
TTGTACCGGCGACGTATCTATCAAGTGTCTGCCTTATCAACTTTCGATGGTAGTTTATGTGCCTACCATGGTTGTAACGG
GTAACGGAGAATAAGGGTTCGACTCCGGAGAGGGAGCCTGAGAAACGGCTACCACATCCAAGGAAGGCAGCAGGCGCGCA
AATTACCCACTCTCAGAATGAGGAGGTAGTGACGAAAAATAACGAGACCGTTCTCTTTGAGGCCGGTTATCGGAATGGGT
ACAATTTAAACCCGTTAACGAGGATCTATGAGAGGGCAAGTCTGGTGCCAGCAGCCGCGGTAATTCCAGCTCTCAAAGTG
TATATCGTCATTGCGCGGTTAAAAAGCTCGTAGTTGGATCTGCGTCTCAGGACCTGGTCCATCCATTGGATGAGAACTAG
GCTCCTAGCTAATATTGCCAGTTTTCCCATACGTTACCTTAATTGGTTGCATATGGTGGCTAGCAAGTTTACCTTGAAAA
AATTAGAGTGCTCAATGCGGGCTAATGCCTGAATACTCGTGCATGGAATAATGAAATAGGATCTCGGTTCTATTTGTTGG
TTTTCTGATCTGAGATAATGGTTAAGAGGGACGGACGGGGGCATTCGTATCGCTGCGTGAGAGGTGAAATTCTTGGACCG
TAGCGAGACGTACGACTGCGAAAGCATTTGCCAAGAATGTCTTCATTAATCAAGAACGAAAGTCAGAGGTTCGAAGGCGA
TCAGATACCGCCCTAGTTCTGACCGTAAACGATACCAACTAGCGTTCCGTCGGCGGTAAATACGCCTTGACGGGCAGCTT
CCCGGAAACGAAAGTTTTTCGGTTCCGGGGGAAGTATGGTTGCAAAGCTGAAACTTAAAGAAATTGACGGAAGGGCACCA
CCAGGAGTGGAGCCTGCGGCTTAATTTGACTCAACACGGGAAAACTCACCTGGCCCGGACACCGTGAGGATTGACAGATT
GAGAGCTCTTTCATGATTCGGTGGTTGGTGGTGCATGGCCGTTCTTAGTTGGTGGAGTGATTTGTCTGGTTTATTCCGAT
AACGAGCGAGACTCTAGCCTACTAAATAGTTACTGGATAATTTTATCGTCCAGACAACTTCTTAGAGGGACAAGCGGTGT
TTAGCCGCATGAAGTTGAGCAATAACAGGTCTGTGATGCCCTTAGATGTCCAGGGCTGCACGCGCGCTACACTGGAGGAA
TCAGCGTGCTGTAACCATTACCGAAAGGTATTGGTAACCCCTTGAAAATCCTCCGTGATCGGGATCGGGAATTGCAATTA
TTTCCCTTGAACGAGGAATTCCTAGTAAGTGTGAGTCATCAGCTCACGTTGATTACGTCCCTGCCCTTTGTACACACCGC
CCGTCGCTGCCCGGGACTGAGCCGTTTCGAGAAAAGCGGAGACTGCTGTTTTGATACCTTTCAAGGGTAGAGATTCTTTG
GTGGAAACTGCTTTAATCGCAGTGGCTTGAACCGGGCAAAAGTCGTAACAAGGTTTCCGTAGGTGAACCTGCAGCAGG
>SMU65657 - Schistosoma mansoni 18S ribosomal RNA gene, complete sequence
AACCTGGTTGATCCTGCCAGTAGTCATATGCTTGTCTCAGAGATTAAGCCATGCATGTCTAAGTACATACCTTAAAACGG
TGAAACCGCGAATGGCTCATTAAATCAGCTATGGTTCCTTAGATCGTAAACGCTACATGGATAACTGTAGTAATTCTAGA
GCTAATACATGCCTTGAATCCCTGACCCGCAAGGGGACGGGTGCATTTATTAGAACAGAACCAACCGGGTGCGGCTTCGG
CTGTGCCTGTTACATTCTGTGATGACTCTGGATAACTTTACTGATCGCAGTCGGCCTTGTGTCGGCGACGGATCTTTCAA
ATGTCTGCCCTATCAATTTGTTGGTAGGTGATTTGCCTACCATGATGATAACGGGTAACGGGGAATCAGGGTTCGATTCC
GGAGAGGGAGCCTGAGAAATGGCTACCACATCCAAGGACGGCAGCAGGCGCGAAAATTACCCACTCCCGGCACGGGGAGG
TAGTGACGAAAAATACGTATACGGGACTCAATTGAGGCTCCGTAATTCGAATGATTACAATTTAAATCCTTTAACGAGGA
CCAATTGGAGGGCAAGTCTGGTGCCAGCAGCCGCGGTAACTCCAGCTCCAAAAGCGTATATTAAAGTTGCTGCAGTTAAA
AAGCTCGTAGTTGAATCTGGGTCGTGCGGTCGCATGCCGTTGCTTGTTCACGGTTTTGGTTACGATCAGGACCAGTGTTC
AGCTCGGTGTAGTGGCTGTGCAGCCTTTCAGCCGTGTCTGTGTTAAACGGGTGCTGGTGGGTTGACRAGTTCGTCTTGTT
GACCTGTCGGCATGCTTCCGGATGCCTTTAAACGGGTGTCGGGAGCGGACGGCATCTTTACTTTGAACAAATTTGAGTGC
TCAAAGCAGGCCTATGTGCCTGAAAATTCTTGCATGGAATAATGAAATAGGACTTCGGTTCTATTTTGTTGGTTTTCGGA
TCCGAAGTAATGGTTAAGAGGGACAGACGGGGGCATTTGTATGGCGGTGTTAGAGGTGAAATTCTGGGATCGCCGCCAGA
CAAACTACAGCGAAAGCATTTGCCAAGAATGTTTTCATTGATCAGGAGCGAAAGTCAGAGTTTCGAAGACGATCAGATAC
CGTCGTAGTTCTGACCATAAACGATGCCAACTGACGATCCGCGTTGGTTCTATAATTGACATCGCGGGCAGTCCCCGGGA
AACCTTTAAGTCTTTGGGCTCCGGGGGGAGTATGGTTGCAAAGCTGAAACTTAAAGGAATTGACGGAAGGGCACCACCAG
GAGTGGAGCCTGCGGTTTAATTSGACTCAACACGGGAAAACTCACCCGGCCCGGACACTGTGAGGATTGACAGATTGATA
GCTCTTTCTTGATTCGGTGGGTGGTGGTGCATGGCCGTTCTTAGTTGGTGGAGCGATTTGTCTGGTTAATTCCGATAACG
AACGAGACTTTAACCTGCTAAATAGTAGACTGGTCCTCTGTGCTCGTTTAGGGCGCGGCTTCTATTGCTTCTTTATGGAG
TAGTGTGGTCGTGATCCGGCGGGTGCGGTGCCAGTTTTTACTTCTTAGAGGGACAAGCGGCACACTTAAGTCGCACGAAA
TTGAGCAATAACAGGTCTGTGATGCCCTTAGATGTCCGGGGCCACACGTGCGCTACAATGACGGTGCCAGCGAGTCTGGA
AACCTGGCCCGAAAGGGTTGGGCAAACTGTTTCATCACCGTCGTGACTGGGATCGGGGCTTGCAATTATTCCCCGTGAAC
GAGGAATTCCTGGTAAGTGCAAGTCATAAGCTTGCGCTGATTACGTCCCTGCCCTTTGTACACACCGCCCGTCGCTACTA
CCGATTGAATGGTTTAGTGAGGTCGTTGGATTGGCGTCGTTGTAGTGGCTTTGCCGCTCGACTGATGCTGAGAAGATGAC
CTAATTTGACTATTTAGAGGAAGTAAAAGTCGTAACAAGGTTTCCGTAGGTGAACCTGCAGAAGGATCA
>AY851265 - Trichuris suis 18S ribosomal RNA gene, complete sequence
TACCTGGTTGATCCTGCCAGTAGTCATATGCTTGTCTCAAAGACTAAGCCATGCAAGTGCAAGCGCACACCGCTGAACGG
TGAAGCCGCGAATGGCTCATTACAGCAGTCATTGTTCGCAAGAACTGATGTCACTTGGATAACTGTGGAAATTCTAGAGC
TAATACTGCCTCGAAGCTCGGTCGCGCGCAACGCTCGGAGCGCATTTATTAGAACAAAACCAATCGGACGCAGGCTATCT
ATTGGTCTGGGTCCGCGAAAGGTTGGTGAATCGGAATAACTATGCTGATCGCACGGTCCAGCACCGGCGACGAACTTTGA
AATGACTTGCTCATCAACTTTCGATGGTACGCTACGTGCTTACCATGGTGACAACGGTTAACGGAGAATCAGGGTTCGAC
TCCGGAGAGGGAGCCTGAGAAACGGCTACCACATCCAAGGAAGGCAGCAGGCACGCAAATTACCCACTCCCAGATCGGGG
AGGTAGTGACGAAAAATAACGGAACGTATCTCCATGAGACGCGTTACCGGAACGACTGAGCCGTACACAAGCTCGGCTAA
ATCTATTGGAGGGCAAGTCTGGTGCCAGCAGCCGCGGTAATTCCAGCTCCAATAGCGTATATTAAAGTTGCTGCGGTTAA
ACCGCTCGTAGTTGGATTGCGGATGTCGACGACGGTCGTCCTAAGCAGGAGTCGTTCCGTCGCTCGTCACCTGTTCGATC
AAGATTGCCCTGGATGCTCTTCAGTGAGTGTCCTTGGCGACTTGAAAGTTTACTTTGAGAAAATGGAAGCGCTCAAGGCA
AGCCGTGATGCTTGAACCGTGGTGCATGGAATAATAGAAAGATGGCCTCAGTGCTATTTTGTTGGTTTGCGGCTATGAGG
CAATGATTAAAAGAGACAGACGGGGACATTCGTATTGCTGCGTTAGAGGTGAAATTCTTGGATCGCAGCAAGACGACCAA
TTGCGAAAGCATTTGTCAAGAATGTTTTCATTAATCAAGAACGAAAGTTAGAGGTTCGAAGGCGATCAGATACCGCCCTA
```

GTTGTGACCGTAAACGATGCCAACCAGCGATTCGGCGACGTTCTTTTTATGACTCGCTGAGCAGCTTCCGGGAAACCAAA
GTGTTTCGGTTCCGGGGGAAGTATGGTTGCAAAGCTGAAACTTAAAGGAATTGACGGAAGGGCACCACCAGGAGTGGAGC
ATGCGGCTTAATTTGACTCAACACGGGAAAACTCACCCGTTCCGAACACTGTGAGGATTGACAGATCAAGAGCTCTTTCT
TGATTCAGTGGGTAGTGGTGCATGGCCGTTCTTAGTTGGTGGAGCGATTTGTCTGGCTAATTCCGATAACGAACGAGACT
CTGGCCTACTAACTAGCGGCGGTATTCACGTCTCTCGCGACGCCGTGCCTACGTGTCGGCTGAGCACAGTCGCCGTTCGC
GCGAGCTCCGCGGACACCTGCAGTCCGGCGACCGCTTCTTAGAGGGACCAGCGACACTTTCGCAAGCCGCACGAGAAAGA
GCAATAACAGGTCTGTGATGCCCTTAGATGTACGGGGCTGCACGCGTGCTACACTGACGGCGTCAGCGTGCGTTCAAGCC
CGGCCTGGCAAGGCCGGGAAATCGGCTGAAACGTTCTCGTGACTGGGACAGGGAATTGCAATTATTTCCCTCGAACGAGG
AATTCCCAGTAAGCGCGAGTCATCAGCTTGCGTTGACTACGTCCCTGCCCTTTGTACACACCGCCCGTCGCTACTACCGA
TTGGATGATTTAGTGAGGCCTTTGGACCGATGCCGCCGGTGTTCGGCCCGGTGGTTGAACCACGGCGTCGGGAAGCTGGC
CGAACCAAACCATCTAGAGGAAGTAAAAGTCGTAACAAGGTTTCCGTAGGTGAACCTGCGGAAGGATCATTA
>JX943583 – Trichomonas vaginalis isolate 003 18S ribosomal RNA gene, partial sequence
TGTCAAGATTAAGCCATGCAAGTGTTAGTTCAGGTAACGAAACTGCGAATAGCTCATTAATACGCTCAGAATCTATTTGG
CGGCGACCAACAGGTCTTAAATGGATAGCAGCAGCAACTCTGGTGCTAATACATGCGATTGTTTCTCCAGATGTGAATTA
TGGAGGAAAAGTTGACCTCATCAGAGGCACGCCATTCGACTGAGTGACCTATCAGCTTGTACTTAGGGTCTTTACCTAGG
TAGGCTATCACGGGTAACGGGCGGTTACCGTCGGACTGCCGGAGAAGGCGCCTGAGAGATAGCGACTATATCCACGGGTA
GCAGCAGGCGCGAAACTTTCCCACTCGAGACTTTCGGAGGAGGTAATGACCAGTTCCATTGGTGCCTTTCGGTACTGTGG
ATAGGGGTACGGTTTTCCACCGTACCGAAACCTAGCAGAGGGCCAGTCTGGTGCCAGCAGCTGCGGTAATTCCAGCTCTG
CGAGTTTGCTCCCATATTGTTGCAGTTAAAACGCCCGTAGTCTGAATTGGCCAGCAATGGTCGTACGTATTTTTACGTTC
ACTGTGAACAAATCAGGACGCTTAGAGTATGGCTACATGAATGACTCAGCGCAGTATGAAGTCTTTGTTTTCTTCCGAAA
ACAAGCTCAATGAGAGCCATCGGGGGTAGATCTATCTCATGACGAGTGGTGGAATACTTTGACTCATGAGAGAGAAGCTG
AGGCGAAGGCGTCTACCTAGAGGGTTTCTGTCGATCAAGGGCGAGAGTAGGAGTATCCAACAGGATTAGAGACCCTGGTA
GTTCCTACCTTAAACGATGCCGACAGGAGTTTGTCATTTGTTAATGGCAGAATCTTTGGAGAAATCATAGTTCTTGGGCT
CTGGGGGAACTACGACCGCAAGGCTGAAACTTGAAGGAATTGACGGAAGGGCACACCAGGGGTGGAGCCTGTGGCTTAAT
TTGAATCAACACGGGGAAACTTACCAGGACCAGATGTTTTTTATGACTGACAGGCTTCGGGTCTTTCAGGATATTGTTTT
TGGTGGTGCATGGCCGTTGGTGGTGCGTGGGTTGACCTGTCTAGCGTTGATTCAGCTAACGAGCGAGATTATCGCCAATT
ATTTACTTTGCCGAAGTCTTTCGGTTAAAGTTCTAATTGGGACTCCCTGCGATTTTAGCAGGTGGAAGAGGGTAGCAATA
ACAGGTCCGTGATGCCCTTTAGATGCTCTGGGCTGCACGCGTGCTACAATGTTAGGATCAATAGGACTGCGAGCCTGAGA
GGGTGCGCTACTCTTATAATCCCTAACGTAGTTGGGATTGACGTTTGTAATCAGCGTCATGAACCAGGAATCCCTTGTAA
ATGTGTGTCAACAACGCACGTTGAATACGTCCCTGCCCTTTGTACACACCGCCCGTCGCTCCTACCGATTGGATGACTCG
GTGAAATCACCGGATGCTTACGAGCAGAAAGTGATTAAATCACGTTATCTAGAGGAAGGAGAAGTCGTACAAAGGGTAAC
GGAAGGGGCCACCGCCCACCGCA
>JQ677148 – Dientamoeba fragilis isolate Df1085 18S ribosomal RNA gene, partial sequence
CTCGTGCTAATACATGAAATTTTAATAATCTTAAATTAAATCAGATTATTTTTAATACCTTTTAATAGGTAATCCAATCG
AATGAGTGACCTATCAGGCCAGTACTTAGGGTCTTTACCTAAGTAAGCTATCACGGGTAACGGGCGGTTACCGTCGTACT
GCCGGAGAAGGCGCCTGAGAGATAGCGACTATATCCACGGGTAGCAGCAGGCGCGAAACTTACCCACTCGAGACTATCGG
AGGTGGTAATGACCAGTTATAATTAAGGAATTTTCCTTATTATATAGGAATATACTTTTCCAGTATATTGTAACCTAGCA
GAGGGCCAGTCTGGTGCCAGCAGCTGCGGTAATTCCAGCTCTGCAAGTTTGCTCCCATATTGTTGTAGTTAAAACGCTCG
TAGTCTGAATTATTTTAATTTAAATTTTTTAAATTAAAATTTAGTTTTTATTTTATAAAAACGTTCACTGTGAACAAATC
AGAACGCTTAAAGTAATTTTCTTTATTGAATGATTTAGCGCAGTATGAAATTTTTACTTTTTAAATTTTAATTAATTTAA
CAAGTAATATCAAAGAGAATAATCGGGGATAGATCTATTTCATGGCGAACAGCGAAATGTTTTGACCCATGAGAGAGAAA
CGAAGGCGAAAGCATCTATCAAGTGTATTTCTATCGATCAAGGGCGAGAGTAGGAGTATCCAACCGGATCAGAGACCCGG
GTAGTTCCTACCTTAAACTATGCCGACAAGGTTTTGTTTTTTTTAATAAAAGCAGTACCATAGGAGAAATCATAGTTCAT
GGGCTCTGGGGGAACTACGACCGCAAGGCTGAAACTTGAAGGAATTGACGGAAGGGCACACCAGGGGTGGAGCTTGTGGC
TTAATTTGAATCAACACGGGAAAACTTACCAGGACCAGATATTTTTAATGACTGATCAGGCTATAGGTCTTTCAGGATAT
GATTTTTGGTGGTGCATGGCCGTTGGTGGTGCGTGGGTTGACCTGTCTAGCGTTGATTCAGATAACGAGCGAGATTATCA
CCAATTAAATATATAAATATTTTTATTAAAATAATTTATTTTCTAATTGGGACTCCCTGCGTCTAAGCAGGAGGAAGATG
GTAGCAATAACAGGTCCGTGATGTCCTTTAGATGCTCTGGGCTGCACGCGCGCTACAATGTTATAATCAAAGAGGTTTGC
TAAATCGATAGATTATCTTTTTTTTTAAAAGATTTCATAGCTACTCTGTTAATATATAACGTAGTTGGGATTGATAATTG
TAATCATTATCATGAACCAGGAATCCCTTGTAAATGCGTGTCAACAACGCGCGTTGAATACGTCCCTGCCCTTTGTACAC
ACCGCCCGTCGCTCCTACCGATTGAATGACTCGGTGAAATCATTAGATCATTTTTTAAATGAAAAGGTGATTAAATCACG
TTATTTAGAGGAAGGAGAAGTCGTAACAAGGTAACGGTAGGTGAACCTGCCGTTGGATCAG
>AB444953 – Entamoeba coli gene for 18S ribosomal RNA, complete sequence
TATCTGGTTGATCCTGCCAGTATTATATGCTTCTGTCAAAGATTAAGCCATGCATGTCTAAGCAAAAAGTCCTAGTAAGA
TGAAGCTGCGAACGGCTCATTACAACAGTTATAATCTTTTTGATGAAATACGTACAAGGATATCTTTGAGAATGTCAAAG
CTAATACTTGACGGGAAACGCCCTCAGTCGAGTGTGTTCCTCCGGGAGCATAATCTACTGAGGAGGGGAGGATCCTTATG
GTCCTTTCAATATATTACCACTTCTTTGTGAATAAGGGTGGATTATATGCCAAGAGAATTGTAGAAATCGAAAGATTTTC
ACAAGTCATCATTAAGAATATCTGACCTATCAACTGGTTGTTGGTAAGGTAGTGGCTTACCAAGGTGATAACGGGTAACG
AGGAATAAGGGTTCGACATCGGAGAGGGAGCTTTAGAAATGGCTACCACTTCTAAGGAAGGCAGCAGGCGCGAAAATTAC
CCAATCTTAACAACTGAAGGAGGTAGTGACGACAATTAACGCTTTCCCTCGCATTTTGTGAGGATTGCCGGAATGATCAC
GACATAAACCATCGTGAGAAAGCGATTGGAGGGCAAGTCTGGTGCCAGCAGCCGCGGTAATTCCAGCTCCAATAGTGTAT
TGTAAAGTTGTTGTGATTAAAACGCTCGTAGTTGAATGTAAACAATCGAGGCATGATTTACGTTCTCTGGCGTGTGAGCT
TGCTCATTGTCAGTAGGACCGCAATCAATGAATGTCTCACTATAAGCGGGAGTTTGATTCAGTTCCAAACTCTTTTACTT
TGATTAAATCAAGGTGCTTAAAGCAAAATCTTCTGTTAATGAATGATGAAGCATGGTATAAAACGAAAAACAGAACAGAA
ATCAATATTGAATTATGAGGATGGCTTGCCATTCGAATTTTTCGGTGTTTTGTTTTGTTTAAAACAAGATTAAGAGAAAT
GATTAGGGTGATTTAGATAGGAACGGGAGAGGTGGAATTCCATGATCGTTTCGAGATAAACGAGAGCGAAAGCATTTCAC
TGAATTCATTTCATTAATCAAGAACGAAAGTCAGGGAATCAAAGACGATTAGATACCGTCGTAGTCCTGACCATAAACGA
TGTCAACTAAGGATTGGATAAGAAATTTTCGAAGTACGGGTTTCTAATATCACCAAGCGACAGCTTGGCGATTCTGAAAC
TGTCTTCAATTCTTCCCTTATTCAGGACTTGATGAGAAATCATTAAGTGAATGGACTTCAGGGGGAGTATGGTCACAAGG
CTGAAACTTAAAGGAATTGACGGAAGGGCACACCAGGAGTGGAGCCTGCGGCTTAATTTGACTCAACACGGGAAAACTTA
CCAAGACCGAACGGTGTAAGGAATGACAGGTTACTGGGTCTTTCATGATTCATCGGGGAGTGGTGCATGGCCGTTCTTAG
TTCGTGGACTGATTTGTCTGGTTAATTCCGATAACGAACGAGACTTGAACTTATTAATTAGTTGGGTGGAAATCATCTTC
GGGTGGTTCTGCTTACTATCCGCAAGGATGGCGTGAAGAGTTTATCTTCGTGACTTCTTAAAGGGACACATCTTCAACGT

TCTCGTATGTTTGACTGGGTTCTCAGAGCAATCTGTTGATCCTAGACAAATGTCGGGAAAAAGAAGAAGGAAGCGTCAAG
CAATAACAGGTCTGTGATGCCCTTAGACATCTTGGGCTGCACGCGTGCTACAATGAAAGTACTAACGAGTTTTCGCTGTT
TGCGGGTGGTAATGAGTTTCGCAACTAATTGCTATTTGCAGGCAGCAATTTCCAAGTCCGAAAGGACAAGGCAAGCTCTA
ATGTCTTTTATTTAATTCTTGACTGGGATGAATGATTGGAATTATTTGTTCTGAACAAGGAATTCCTTGTAAGCGCAAGT
CATTACCTTGCGCTGAATAAGTCCCTGCCCTTTGTACACACCGCCCGTCGCTCCTACCGATTGAATAAAGAGGTGAAATC
TCAGGATTTTGGTGGCTTGCCATTGGGAGAATGAGAGTAAATCTCTTTGTTTAGAGGAAGGAGAAGTCGTAACAAGGTTT
CCGTAGGTGAACCTGCGGAAGGATCA
>KF250434 - Entamoeba gingivalis strain E 18S ribosomal RNA gene, partial sequence
TGTAGAAATGAAATACATTTTGACAAGGAATCAATGAAAATATCTGATCTATCAACTAGTTGGTAGTATAGAGGACTACC
AAGGTTATAACGGATAACGAGAAATTAGGGTTTGACATCGGAGAAGGAGCTTTCAAAATGGCTACTACTTCTAAGGAAGG
CAGCAGGCGCGTAAATTACCCACTTTTAACAGAAAGAGGTAGTGACGACAAATAACTCTATTCTTTAACAAAAAGAATTG
AAGGAATGAACGGAACGTACATAGTTTTGTGAAAGCAATTGGAGGGCAAGTCTGGTGCCAGCAGCCGCGGTAATTCCAGC
TCCAATAGTATATATTAAAGTTGTTGTGATTAAAAGGCTCGTAGTTGAATGAAGATACTATTGAAAAGGCTTTTCTTTTT
ATACAAAAAGAGAAGTTTAGTTAAACAATAGAAGAAGGAAATGGATTACTTTGAATAAAATAGAGTGTTTAAAGCAAAAC
AATGTTAATGAATAATGAAGCATGGGACAATAAGAAGGAGATTTGAAAGGATTTCGAGAAGAAGATTAAAAGGAATAATT
GGGGTAATTTAGAAAAAGATGGGAGAGGTGAAAATCCATGATCATCTTTAGATAAACGAGAGCGAAAGCATTTTACT
>AB426549 - Entamoeba histolytica gene for 18S ribosomal RNA, complete sequence
TATCTGGTTGATCCTGCCAGTATTATATGCTGATGTTAAAGATTAAGCCATGCATGTGTAAGTATAAAGACCAAGTAGGA
TGAAACTGCGGACGGCTCATTATAACAGTAATAGTTTCTTTGGTTAGTAAAATACAAGGATAGCTTTGTGAATGATAAAG
ATAATACTTGAGACGATCCAGTTTGTATTAGTACAAAATGGCCAATTTATTTAAATGAATTGAGAAATGACATTCTAAGT
GAGTTAGGATGCCACGACAATTGTAGAACACACAGTGTTTAACAAGTAACCAATGAGAATTTCTGATCTATCAATCAGTT
GGTAGTATCGAGGACTACCAAGATTATAACGGATAACGAGGAATTGGGGTTCGACATCGGAGAGGGAGCTTTACAGATGG
CTACCACTTCTAAGGAAGGCAGCAGGCGCGTAAATTACCCACTTTCGAATTGAAGAGGTAGTGACGACACATAACTCTAG
AGTTGAGTAAAATCAATTCTTGAAGGAATGAGTAGGAGGTAAATTCTCCTACGAAATCAATTGGAGGGCAAGTCTGGTGC
CAGCAGCCGCGGTAATTCCAGCTCCAATAGTGTATATTAAAGTTGCTGTGATTAAAACGCTCGTAGTTGAATTAAAATGT
GATTTTATACATTTTGAAGACTTTGCATAAGTAAAGTTTCTAGAAATGTTAAATTAAAATCAAAGAAGGAAACAATTCAA
GTAATTGAGTTGTTATTACTTTGAATAAAATAAGGTGTTTAAAGCAAAACATTATGTTAATGAATATTCAAGCATGGGAC
AATGCTGAGGGGATGTCAATAAGACATTTCGAGAGAAGGATTAAAAGGAACAATTGGGGTGATTCAGAAAATAACGGGAG
AGGTGAAAATCCATGATCGCTATAAGATGCACGAGAGCGAAAGCATTTCACTCAACTGTGTCCATTAATCAAGAACGAAA
GTTAGGGGATCGAAGACGATCAGATACCGTCGTAGTCCTAACTATAAACGATGTCAACCAAGGATTGGATGAAATTCAGA
TGTACAAAGATGAAGAAGCATTGTTTCTTAATCTGAGTATATCAATATTACCTTGTTCAGAACTTAAAGAGAAATCTTGA
GTTTATGGACTTCAGGGGGAGTATGGTCACAAGGCTGAAACTTAAAGGAATTGACGGAAGGGCACACCAGGAGTGGAGCC
TGCGGCTTAATTTGACTCAACACGGGAAAACTTACCAAGACCGAACAGTAGAAGGAATGACAGATTAAGAGTTCTTTCAT
GATTTATTGGGTAGTGGTGCATGGCCGTTCTTAGTTGGTGGAGTGATTTGTCAGGTTAATTCCGGTAACGAACGAGACTG
AAACCTATTAATTAGTTTTCTGCCTATAAGACAGAAATGTTCGCAAGAACAGGTGCGTAAGTACCACTTCTTAAAGGGAC
ACATTTCAATTGTCCTATTTTAATTGTAGTTATCTAATTTCGGTTAGAGCTCTTTTAACGTGGGAAAAAGAAAAAGGAAG
CATTCAGCAATAACAGGTCTGTGATGCCCTTAGACATCTTGGGCCGCACGCGCGCTACAATGGAGTTACTAGAGAGCATT
TTATCATTTACACCTTATTTATTAGGCTTTGTCTAATAATTAAGGATAGTGAGTGGTGTACCGAGATTGAAATAGTTAAG
GAAAACTCAAAAGAACGTACATGACAGGGATAAATGATTGGAATTATTTGTTTTGAACGAGGAATTCCTTGTAATATCGA
GTCATTAACTCGAGATGAATACGTCCCTGCCCTTTGTACACACCGCCCGTCGCTCCTACCGATTGAATAAAGAGGTGAAA
TTCTAGGATTCTGTCTTATAGATAGAAAAATGGATTTAAATCTCCTTATTTAGAGGAAGGAGAAGTCGTAACAAGGTTTC
CGTAGGTGAACCTGCGGAAGGATCAT
>KP722600 - Entamoeba dispar isolate ED_IQ5 18S ribosomal RNA gene, partial sequence
ATCTGGTTGATCCTGCCAGTATTATATGCTGATGTTAGAGATTAAGCCATGCATGTGTAAGTATAAAGACCAAGTAGGAT
GAAACTGCGGACGGCTCATTATAACAGTAATAGTTTCTTTGGTTAGTAAAGTACAAGGATAGCTTTGTGAATGATAAAGA
TAATACTTGAGACGATCCAATTTGTATTAGTACAAAGTGGCCAATTTATGTAAGTAAATTGAGAAATGACATTCTAAGTG
AGTTAGGATGCCACGACAATTGTAGAACACACAGTGTTTAACAAGTAACCAATGAGAATTTCTGATCTATCAATCAGTTG
GTAGTATCGAGGACTACCAAGATTATAACGGATAACGAGGAATTGGGGTTCGACATCGGAGAGGGAGCTTTACAGATGGC
TACCACTTCTAAGGAAGGCAGCAGGCGCGTAAATTACCCACTTTCGAATTGAAGAGGTAGTGACGACACATAACTCTAGA
GTTGAGTAAAATCAATTCTTGAAGGAATGAGTAGGAGGTAAATTCTCCTACGAAATCAATTGGAGGGCAAGTCTGGTGCC
AGCAGCCGCGGTAATTCCAGCTCCAATAGTGTATATTAAAGTTGCTGTGATTAAAACGCTCGTAGTTGAATTAAAATGTG
ATTTTATACATTTTGAAGACTTTANNTAAGTGAAGTTTCTAGAAATGTTAAATTAAAATCAAAGAAGGAGACNNTTCAAG
TAATTGAGTTGTTATTACTTTGAATAAAATAAGGTGTTTAAAGCAAAACATTATGTTAATGAATATTCGAGCATGGGACA
ATGCTGAGGGGATGTCAATTAGACATTTCGAGAGAAGGATTAAAAGGAACAATTGGGGTGATTCAGAAAATAACGGGAGA
GGTGAAAATCCATGATCGGTATAAGATGCACGAGAGCGAAAGCATTTCACTCAACTGGGTCCATTAATCAAGAACGAAAG
TTAGGGGATCGAAGACGATCAGATACCGTCGTAGTCCTAACTATAAACGATGTCAACCAAGGATTGGATGAAATTCAGAT
GTACAAAGATGAAGAAACATTGTTTCTAAATCCAAGTATATCAATACTACCTTGTTCAGAACTTAAAGAGAAATCTTGAG
TTTATGGACTTCAGGGGGAGTATGGTCACAAGGCTGAAACTTAAAGGAATTGACGGAAGGGCACACCAGGAGTGGAGCCT
GCGGCTTAATTTGACTCAACACGGGAAAACTTACCAAGACCGAACAGTAGAAGGAATGACAGATTAAGAGTTCTTTCATG
ATTTATTGGGTAGTGGTGCATGGCCGTTCTTAGTTGGTGGAGTGATTTGTCAGGTTAATTCCGGTAACGAACGAGACTGA
AACCTATTAATTAGTTTTCTGCCTATAAGACAGAAATGTTCGCAAGAACAGGTGCGTAAGTACCACTTCTTAAAGGGACA
CATTTCAATTGTCCTATTTTAATTGTTAGTTATCTAATTTCGATTAGAACTCTTTTAACGTGGGAAAAAGAAAAAGGAAG
CATTCAGCAATAACAGGTCTGTGATGCCCTTAGACATCTTGGGCCGCACGCGCGCTACAATGGAGTTACTAGAGAGCATT
TTATCATTTACACCTTATTTATTAGGCTATGTCTAATAGGTAGGGATAGTAAGTGGTGTACCGAGATTGAAATAGTTAAG
GAAAACTCAAAAGAACGTACATGACAGGGATAAATGATTGGAATTATTTGTTTTGAACGAGGAATTCCTTGTAATATCGA
GTCATTAACTCGAGATGAATACGTCCCTGCCC
>Z11976 - S.haematobium gene encoding 18S ribosomal RNA
AAACTGGTTGATCCTGCCAGTAGTCATATGCTTGTCTCAGAGATTAAGCCATGCATGTCTAAGTACATACCTTAAAACGG
TGAAACCGCGAATGGCTCATTAAATCAGCTATGGTTCCTTAGATCGTAAATGCTACATGGATAACTGTAGTAATTCTAGA
GCTAATACATGCCTTGAATCCCTGACCCGCAAGGGAACGGGTGCATTTATTAGAACAGAACCAATCGGGCGCGGCTTCGG
CTGTGCCTGCTACATTCTGTGATGACTCTGGATAACTTTACTGATCGCAGTCGGCCTTGTGTCGGCGACGGATCTTTCAA
ATGTCTGCCCTATCAATTTGTTGGTAGGTGATTTGCCTACCATGATGATAACGGGTAACGGGGAATCAGGGTTCGATTCC
GGAGAGGGAGCCTGAGAAATGGCTACCACATCCAAGGACGGCAGCAGGCGCGAAAATTACCCACTCCCGGCACGGGGAGG

TAGTGACGAAAAATACGGATACGGGACTCAATTGAGGCTCCGTAATTCGAATGAGTACAATTTAAATCCTTTAACGAGGA
CCAATTGGAGGGCAAGTCTGGTGCCAGCAGCCGCGGTAATTCCAGCTCCAAAAGCGTATATTAAAGTTGCTGCAGTTAAA
AAGCTCGTAGTTGGATCTGGGTCGTGCGGTCGCATGCCGCTGCTTGTTCACGGTTTTGGTTACGATCAGGACGTGTTCAG
CTCGGTGTAGTGGCTGTGCAGCCTTTCAGCCGTGTCTGTGTTAAACGGGTGCTGGTGGGTTGACGAGTTCGTCTTGTTGA
CCTGTCGGCATGCTTCCGGATGCCTTTAAACGGGTGTCGGGAGCGGACGGCATCTTTACTTTGAACAAATTTGAGTGCTC
AAAGCAGGCCTGTGCGCCTGAAAATTCTTGCATGGAATAATGAAATAGGACTTCGGTTCTATTTTGTTGGTTTTCGGATC
CGAAGTAATGGTTAAGAGGGACAGACGGGGGCATTTGTATGGCGGTGTTAGAGGTGAAATTCTGGGATCGCCGCCAGACA
AACTACAGCGAAAGCATTTGCCAAGAATGTTTTCATTGATCAGGAGCGAAAGTCAGAGTTTCGAAGACGATCAGATACCG
TCGTAGTTCTGACCATAAACAATGCCGACTGATGATCCGCGTTGGTTCTATAATTGACATCGCAGGCAGTCCCCGGGAAA
CCTTTAAGTCTTTGGGCTCCGGGGGGAGTATGGTTGCAAAGCTGAAACTTAAAGGAATTGACGGAAGGGCACCACCAGGA
GTGGAGCCTGCGGTTTAATTCGACTCAACACGGGAAAACTCACCCGGCCCGGACACTGTGAGGATTGACAGATTGATAGC
TCTTTCTTGATTCGGTGGGTGGTGGTGCATGGCCGTTCTTAGTTGGTGGAGCGATTTGTCTGGTTAATTCCGATAACGAA
CGAGACTTTAACCTGCTAAATAGTAGACTGGTCCTCTGTGCTCGTTTAGGGCGCGGCTTCTATTGCTTCTTTATGGAGTA
GTGTGGTCGTGATCCGGCGGGTGCGGTGCCAGTTTTTACTTCTTAGAGGGACAAGCGGCACACTTAAGTCGCACGAAATT
GAGCAATAACAGGTCTGTGATGCCCTTAGATGTCCGGGGCCACACGTGCGCTACAATGACGGTGCCAGCGAGTCTGGGAA
CCTGGCCCGAAAGGGTTGGGCAAACTGTTTCATCACCGTCGTGACTGGGATCGGGGCTTGCAATTATTCCCCGTGAACGA
GGAATTCCTGGTAAGTGCAAGTCATAAGCTTGCGCTGATTACGTCCCTGCCCTTTGTACACACCGCCCGTCGCTACTACC
GATTGAATGGTTTAGTGAGGTCGTTGGATTGGTGTCGTTGTAGTGGCGTTGCCGCTCGACTGATGCTGAGAAGATGACCT
AATTTGACTATTTAGAGGAAGTAAAAGTCGTAACAAGGTTTCCGTAGGTGAA
>AY618266 – Blastocystis hominis isolate 989 18S ribosomal RNA gene, partial sequence
GGAAGCTTATCTGGTTGATCCTGCCAGTAGTCATACGCTCGTCTCAAAGATTAAGCCATGCATGTGTAAGTGTAAATATC
AAAGTTTGGAACTGCGAATGGCTCATTATATCAGTTATAGTTTATTTGGTGAAGTGTACTACTTGGATAACCGTAGTAAT
TCTAGGGCTAATACATGAGAAAGTCCTCTGGTGAGGTGTGTTTATTAGAATGAAAACCATATGCTTCGGCATGATAGTGA
GTAATAGTAACCTATCGTATCGCATGCTTAATGTAGCGATGAGTCTTTCAAGTTTCTGCCCTATCAGCTTTCGATGGTAG
TATATGGGCCTACCATGGCAGTAACGGGTAACGAAGAATTTGGGTTGATTTCGGAGAGGGAGCCTGAGAGATGGCTACCA
CATCCAAGGAAGGCAGCAGGCGCGTAAATTACCCAATCCTGACACAGGGAGGTAGTGACAATAAATCACAATGCGGGACT
ATACGTCTTGCAATTGGATTGAGAACAATGTACAACTCTTATCGATAAGCCATTGGAGGGCAAGTCTGGTGCCAGCAGCC
GCGGTAATTCCAGCTCCAATAGCGTATATTAACGTTGTTGCAGTTAAAAAGCTCGTAGTTGAAGTGTGGGTGATCGCTGT
TGTGAGACTTCGGTCTCTCGACAGTAAGTCACCCCCTTCCAGTATCCAGTAGTGGGTATTCACTTACTTACTATTGTGTG
TTGGTCCTTTACTGTGAGAAAATTAGAGTGTTCAAAGCAGGCGTTTGCTTGAATAGATTAGCATGGAATAATAATTGAAG
GCTTTCGTGTTCGATTTGATTGGTTTGTTCATGGAAGCAAGGTTAAAAGGAACAGTTGGGGGTATTCATATTCACTAGTT
AGAGGTGAAATTCTCGGATTTATGGAAGATGAACAAGTGCGAAAGCATTTACCAAGGATGTTTTCATTAATCAAGAACGA
AAGCTAGGGGATCGAAGAGGATTAGATACCCTCGTAGTCTTAGCTATAAACGATACCGACTAGGGGTTAGTAGAGGTCAA
AGTGTCTTTATTAGTACCTTATGAGAAATCAAAGTCTTTGGGTTCCGGGGGGAGTATGGTCGCAAGGCTGAAACTTAAAG
GAATTGACGGAAGGGCACCACCAGGAGTGGAGCCTGCGGCTTAATTTGACTCAACACGGGGAAACTTACCAGGTCCAGAC
ATAGGAAGGATTGACAGATTGATAGCTCTTTCTTGATTCTATGGGTGGTGGTGCATGGCCGTTCTTAGTTGGTGGAGTGA
TTTGTCTGGCTAATTCCGATAACGAACGAGACCTCCGCCTTTAACTAGTGACGTGTATTGTGATATGCGTTGCTTCTTAT
AGGGACACTATATGTAAAATGTAGGGAAGCTGGAGGCAATAACAGGTCTGTGATGCCCTTAGATGTTCTGGGCTGCACGC
GCGCGACACTGATCTATTCAACGAGTGGCTGGGTCGAGAGACTTGGCAAATCTTGTGAAAGTAGATCGTGATGGGGATTG
ATGCTTGTAATTGTTCATCATGAACGAGGAATTCCTAGTAAACGCAAGTCATCAACTTGCATTGATTACGTCCCTGCCCT
TTGTACACACCGCCCGTCGCACCTACCGATTGAATGGTCCGGTGAACACTTTGGATTTGAGATTGGTTGTCTGTAAAAGG
ATGGTTAATCTGGGAGAAGTCGTGTAAATCTTATCATTTAGAGGAAGGTGAAGTCGTAACAAGGTTTCCGTAGGTGAACC
TGCGGAAGGATCAGGATCCC
>STDRG18S – Strongyloides stercoralis 18S ribosomal RNA gene, complete cds
ACCTGGTTGATCCTGCCAGTAGTCATATGCTTGTCTCAAAGATTAAGCCATGCATGTCTAAGTATAAACAAATTCATACT
GTGAAACTGCGAATGGCTCATTAAATCAGTTATAGTTTATTTGATGGTTTCTTGCTACATGGATAACTGTGGTAATTCTA
GAGCTAATACATGCTKAAAAGCCCCGACTTCTGGAAGGGGTGTATTTATTAGATAAAAAACCAATGACTTCGGGCTCCTT
GGTGATTCATAATAACTTCTCGAATCGCATGGCCTTGCGCCGGCGATGCTTCATTCAAATTTCTGCCCTATCAACTTTCG
ATGGTAGGATAGTGGCCTACCATGGTATCAACGGGTAACGGGGAATTAGGGTTCGATTCCGGAGAGGGAGCCTGAGAAAC
GGCTACCACATCCAAGGAAGGCAGCAGGCGAAAATTACCCAATTTTAGTTAAAAGAGGTAGTGACGAAAAATGACAAC
CAAATATTATTATTAATATTTGGATTGAAAATCTTCAAGTTTAAATMACCTTGTTGGTAAAGGAAAGGGCAAGTCTGGTG
CCAGCAGCCGCGGTAATACCAGCTTTCCAAGTGCATAAAATGATTGTTGTGGTTAAAAAGCTCGTAGTTGGATTATAAAG
ATTGTATAATGAGCATCTTGGATGTTATTTAATCATTATCATCTTATATTTTTATTATATTAGAAATAATATAATAACTG
TCACTTTGAATAAATCAGAGGGTTTAAACCAGACATTATATGTTTGTATGGTCTAGCATGGAATAACACTATAGAAAAAT
TTAGTGTGGTTTCACTTAATTTTTCATGATTAATAGGAACAAACGGGGGCATTCGTATCGCTACGTTAGAGGTGAAATTC
TTGGACCGTAGCGAGACGTCCTACTGCGAAAGCATTTGCCAAGAATGTTTTCATTAATCAAGAACGAAAGTTAGAGGTTC
GAAGGCGATCAGATACCGCCCTAGTTCTAACCGTAAACTATGCCTACTAGATGTATGAATTATTAGTTATAATTATTTAT
GCATCTTCTCGGAAACGAAAGTCTTTCGGTTCCGGGGGAAGTATGGTTGAAAAGCTGAAACTTAAAGGAATTGACGGAAG
GCACCACCAGGAGTGGAGCCTGCGGCTTAATTTGACTCAACACGGGAAAACTCACCCGGGCCGGACACTATAAGGATTGA
CAGATTGATAGCTCTTTCATGATTTAGTGGTTGGTGGTGCATGGCCGTTCTTAGTTCGTGGATATGATTTGTCTGGTTGA
TTCCGATAACGAGCGAGACTTTTATGTTATATTAAATATAATTATTTTGTTTATTTTAATATAAATAATTAATATTTTAA
TAACAGATTAATAGTGTTTAACTATTTGAGAGAGAGCGATAACAGGTCTGTGATGCCCTTAGATGTCCGGGGCTGCACGC
GCGCTACAATGTAGTGATCATTATGTTCCTGTTTAGAGATAAATGGGTAAACATTGAAAACATTACGTAACTGGGAGTGA
AAATTGCAATTATTTTTCATGAACGAGGAATTCCAAGTAAACGTAAGTCATTAGCTTACATTGATTACGTCCCTGCCCTT
TGTACACACCGCCCGTCGCTGCCCGGAACTGAGCAATATCCAGAGGCAGGAAGAGATGTAATAAATTTTTAATTTTTTTT
ATATTAAATCCTTCCAATCGCTGTTGTTTGAACCGGGCAAAAGTCGTAACAAGGTTTTCGTAGGTGAACCTGCAGAAGGA
TCATCA
>AF222998 – Cryptosporidium parvum 18S ribosomal RNA gene and internal transcribed spacer 1, complete sequence; and 5.8S ribosomal RNA gene, partial sequence
AACCTGGTTGATCCTGCCAGTAGTCATATGCTTGTCTCAAAGATTAAGCCATGCATGTCTAAGTATAAACTTTTATACGG
TTAAACTGCGAATGGCTCATTATAACAGTTATAGTTTACTTGATAATCTTTTACTTACATGGATAACCGTGGTAATTCTA
GAGCTAATACATGCGAAAAAACTCGACTTTATGGAAGGGTTGTATTTATTAGATAAAGAACCAATATAATTGGTGACTCA
TAATAACTTTACGGATCACAATTAATGTGACATATCATTCAAGTTTCTGACCTATCAGCTTTAGACGGTAGGGTATTGGC

CGACCGTGGCAATGACGGGTAACGGGGAATTAGGGTTCGATTCCGGAGAGGGAGCCTGAGAAACGGCTACCACATCTAAG
GAAGGCAGCAGGCGCGCAAATTACCCAATCCTAATACAGGGAGGTAGTGACAAGAAATAACAATACAGGACTTTTTGGTT
TTGTAATTGGAATGAGTTAAGTATAAACCCCTTTACAAGTATCAATTGGAGGGCAAGTCTGGTGCCAGCAGCCGCGGTAA
TTCCAGCTTCAATAGCGTATATTAAAGTTGTTGCAGTTAAAAAGCTCGTAGTTGGATTTCTGTTAATAATTTATATAAAA
TATTTTGATGAATATTTATATAATATTAACATAATTCATATTACTATTTTTTTTTTTAGTATATGAAATTTTACTTTGAGA
AAATTAGAGTGCTTAAAGCAGGCATATGCCTTGAATACTCCAGCATGGAATAATATTAAAGATTTTTATCTTTTTTATTG
GTTCTAAGATAAGAATAATGATTAATAGGGACAGTTGGGGGCATTTGTATTTAACAGTCAGAGGTGAAATTCTTAGATTT
GTTAAAGACAAACTAATGCGAAAGCATTTGCCAAGGATGTTTTCATTAATCAAGAACGAAAGTTAGGGGATCGAAGACGA
TCAGATACCGTCGTAGTCTTAACCATAAACTATGCCAACTAGAGATTGGAGGTTGTTCCTTACTCCTTCAGCACCTTATG
AGAAATCAAAGTCTTTGGGTTCTGGGGGGAGTATGGTCGCAAGGCTGAAACTTAAAGGAATTGACGGAAGGGCACCACCA
GGAGTGGAGCCTGCGGCTTAATTTGACTCAACACGGGAAAACTCACCAGGTCCAGACATAGGAAGGGTTGACAGATTGAT
AGCTCTTTCTTGATTCTATGGGTGGTGGTGCATGGCCGTTCTTAGTTGGTGGAGTGATTTGTCTGGTTAATTCCGTTAAC
GAACGAGACCTTAACCTGCTAAATAGACATAAGAAATATTATATTTTTTATCTGTCTTCTTAGAGGGACTTTGTATGTTT
AATACAGGGAAGTTTTAGGCAATAACAGGTCTGTGATGCCCTTAGATGTCCTGGGCCGCGCGCGCGCTACACTGATGCAT
CCATCAAGTATATATTCCTGTTTCGAAGGAAATGGGTAATCTTTTGAATATGCATCGTGATGGGGATAGATCATTGCAAT
TATTGATCTTGAACGAGGAATTCCTAGTAAGCGCAAGTCATCAGCTTGCGCTGATTACGTCCCTGCCCTTTGTACACACC
GCCCGTCGCTCCTACCGATTGAATGATCCGGTGAATTATTCGGACCATACTTTGTAGCAATACATGTAAGGAAAGTTTCG
TAAACCTTATCATTTAGAGGAAGGAGAAGTCGTAACAAGGTTTCCGTAGGTGAACCTGCGGAAGGATCATTCTTATTTGA
GAAATGAGTTTTTGATATATTATATTATATTATATTATATTATATTATATTATATTATATATATATGTCATTTAAAAATT
AAATGAACATGAACAAAAAGAAAATTTAATTTTGTTATTTATAATATAAGATTTATTTTATTTTATTTTATTTTATTCTT
TTTAATATATTTAAAAGAAAAGAAAATATAATAAAATAATAAAAGTAGAAATAACATTTTGTTTTCTTTTTATTTTTATT
TTTATTATATTATAAAATATTATTTTTATGTGTATATATAAAGAAAAATATATATATGTATATATAGATATAATAATAAA
TTTGTAATATAATAAAATAATTTTTTTAAAAAACATATAAAATAATTTGTCATTTTTCTTTTCTCACTTTTTCTTTTTTT
TTTTTTCTTTTGCTTTTTTCTTTTTTTTTCTACTTTTTGAATTAAAAGAGAAAAAATAAAAAAGTAAAAGTTTAAAAAAA
AAAAAAAGTTTATGGAAAATAAAAATAAAATTAAATAACCTAACACTTTAAGTAATGGATGTCTTGGTTCTCATAACCGA
TGAAGAACGCAGCAAAACGC
>DQ286403 - Cryptosporidium hominis 18S ribosomal RNA gene, partial sequence
GTTGATCCTGCCAGTAGTCATATGCTTGTCTCAAAGATTAAGCCATGCATGTCTAAGTATAAACTTTTATACGGTTAAAC
TGCGAATGGCTCATTATAACAGTTATAGTTTACTTGATAATCTTTTACTTACATGGATAACCGTGGTAATTCTAGAGCTA
ATACATGCGAAAAAACTCGACTTTATGGAAGGGTTGTATTTATTAGATAAAGAACCAATATAATTGGTGACTCATAATAA
CTTTACGGATCACAATTAATGTGACATATCATTCAAGTTTCTGACCTATCAGCTTTAGACGGTAGGGTATTGGCCTACCG
TGGCAATGACGGGTAACGGGGAATTAGGGTTCGATTCCGGAGAGGGAGCCTGAGAAACGGCTACCACATCTAAGGAAGGC
AGCAGGCGCGCAAATTACCCAATCCTAATACAGGGAGGTAGTGACAAGAAATAACAATACAGGACTTTTTGGTTTTGTAA
TTGGAATGAGTTAAGTATAAACCCCTTTACAAGTATCAATTGGAGGGCAAGTCTGGTGCCAGCAGCCGCGGTAATTCCAG
CTCCAATAGCGTATATTAAAGTTGTTGCAGTTAAAAAGCTCGTAGTTGGATTTCTGTTAATAATTTATATAAAATATTTT
GATGAATATTTATATAATATTAACATAATTCATATTACTATTTTTTTTTTTAGTATATGAAATTTTACTTTGAGAAAATT
AGAGTGCTTAAAGCAGGCATATGCCTTGAATACTCCAGCATGGAATAATATTAAAGATTTTTATCTTTTTTATTGGTTCT
AAGATAAGAATAATGATTAATAGGGACAGTTGGGGGCATTTGTATTTAACAGTCAGAGGTGAAATTCTTAGATTTGTTAA
AGACAAACTAATGCGAAAGCATTTGCCAAGGATGTTTTCATTAATCAAGAACGAAAGTTAGGGGATCGAAGACGATCAGA
TACCGTCGTAGTCTTAACCATAAACTATGCCAACTAGAGATTGGAGGTTGTTCCTTACTCCTTCAGCACCTTA
>TOXRRE - Toxoplasma gondii 18S ribosomal RNA gene, complete sequence
TCATATGCTTGTCTTAAAGATTAAGCCATGCATGTCTAAGTATAAGCTTTTATACGGCTAAACTGCGAATGGCTCATTAA
AACAGTTATAGTTTATTTGATGGTCTTTACTACATGGATAACCGTGGTAATTCTATGGCTAATACATGCGCACATGCCTC
TTCCCCTGGAAGGGCAGTGTTTATTAGATACAGAACCAACCCACCTTCCGGTGGTCCTCAGGTGATTCATAGTAACCGAA
CGGATCGCGTTGACTTCGGTCTGCGACGGATCATTCAAGTTTCTGACCTATCAGCTTTCGACGGTACTGTATTGGACTAC
CGTGGCAGTGACGGGTAACGGGGAATTAGGGTTCGATTCCGGAGAGGGAGCCTGAGAAACGGCTACCACATCTAAGGAAG
GCAGCAGGCGCGCAAATTACCCAATCCTGATTCAGGGAGGTAGTGACAAGAAATAACAACACTGGAAATTTCATTTCTAG
TGATTGGAATGATAGGAATCCAAACCCCTTTCAGAGTAACAATTGGAGGGCAAGTCTGGTGCCAGCAGCCGCGGTAATTC
CAGCTCCAATAGCGTATATTAAAGTTGTTGCAGTTAAAAAGCTCGTAGTTGGATTTCTGCTGGAAGCAGCCAGTCCGCCC
TCAGGGGTGTGCACTTGGTGAATTCTAGCATCCTTCTGGATTTCTCCACACTTCATTGTGTGGAGTTTTTTCCAGGACTT
TTACTTTGAGAAAATTAGAGTGTTTCAAGCAGCTTGTCGCCTTGAATACTGCAGCATGGAATAATAAGATAGGATTTCGG
CCCTATTTTGTTGGTTTCTAGGACTGAAGTAATGATTAATAGGGACGGTTGGGGGCATTCGTATTTAACTGTCAGAGGTG
AAATTCTTAGATTTGTTAAAGACGAACTACTGCGAAAGCATTTGCCAAAGATGTTTTCATTAATCAAGAACGAAAGTTAG
GGGCTCGAAGACGATCAGATACCGTCGTAGTCTTAACCATAAACTATGCCGACTAGAGATAGGAAAACGTCATGCTTGAC
TTCTCCTGCACCTTATGAGAAATCAAAGTCTTTGGGTTCTGGGGGGAGTATGGTCGCAAGGCTGAAACTTAAAGGAATTG
ACGGAAGGGCACCACCAGGCGTGGAGCCTGCGGCTTAATTTGACTCAACACGGGGAAACTCACCAGGTCCAGACATAGGA
AGGATTGACAGATTGATAGCTCTTTCTTGATTCTATGGGTGGTGGTGCATGGCCGTTCTTAGTTGGTGGAGTGATTTGTC
TGGTTAATTCCGTTAACGAACGAGACCTTAACCTGCTAAATAGGATCAGGAACTTCGTGTTCTTGTATCACTTCTTAGAG
GGACTTTGCGTGTCTAACGCAAGGAAGTTTGAGGCAATAACAGGTCTGTGATGCCCTTAGATGTTCTGGGCTGCACGCGC
GCTACACTGATGCATCCAACGAGTTTATAACCTTGGCCGATAGGTCTAGGTAATCTTGTGAGTATGCATCGTGATGGGGA
TAGATTATTGCAATTATTAATCTTCAACGAGGAATGCCTAGTAGCGCAAGTCAGCACGTTGCGCCGATTACGTCCCTGCC
CTTTGTACACACCGCCCGTCGCTCCTACCGATTGAGTGTTCCGGTGAATTATTCGGACCGTTTTGTGGCGCGTTCGTGCC
CGAAATGGGAAGTTTTGTGAACCTTAACACTTAGAGGAAGGAGAAGTCGTAACAAGGT
>FJ236336 - Eimeria maxima clone 158-43 18S ribosomal RNA gene, partial sequence
TAGTCATATGCTTGTCTCAAAGATTAAGCCATGCATGTCTAAGTATAAACTTTTATACGGTGAAACTGCGAATGGCTCAT
TAAAACAGTTATAGTTTATTTGATGGTCTTTTTTACATGGATAACCATGGTAATTCTATGGCTAATACATGCGCAAAAGC
TACCTTCGTTGGAGGAGCTGTGTTTATTAGATACAAAACCAACCCACTTGTTGTGGAGTCTTGGTGATTCATAGTAACCG
AACGGATCGCAGTTTGCCTTCGGGCTCGCGATGGATCATTCAAGTTTCTGACCTATCAGCTTTCGGCGGTAGGGTATTGG
CCTACCGTGGCAGTGACGGGTAACGGGGAATTAGGGTTCGATTCCGGAGAGGGAGCCTGAGAAACGGCTACCACATCTAA
GGAAGGCAGCAGGCGCGCAAATTACCCAATGAAAACAGTTTCGAGGTAGTGACGAGAAATAACAATACAGGGCATTTTAT
GCTTTGTAATTGGAATGATGGGAATGTAAAACCCTTCCAGAGTAACAATTGGAGGGCAAGTCTGGTGCCAGCAGCCGCGG
TAATTCCAGCTCCAATAGTGTATATTAGAGTTGTTGCAGTTAAAAAGCTCGTAGTTGGATTTCTGTCGTGGTCAGCCTGT
GCTGCCCGTATGGGTGTGCGCGTGGTTTGCCCTCGGCATTCTTCCGGTAGCTTGTGGCGCTTAAATGCGTCTGCAAGTGT

TCTGGAACTTTTACTTTGAGAAAAATAGAGTGTTTCAAGCAGGCTTGTCGCCCTGAATACTGCAGCATGGAATAATAGGA
TAGGACCTCGGTTCTATTTTGTTGGTTTCTAGGACCAAGGTAATGATTAATAGGGACAGTTGGGGGCATTCGTATTTAAC
TGTCAGAGGTGAAATTCTTAGATTTGTTAAAGACGAACTACTGCGAAAGCATTTGCCAAGGATGTTTTCATTAATCAAGA
ACGACAGTAGGGGGTTTGAAGACGATTAGATACCGTCGTAATCTCTACCATAAACTATGCCGACTAGAGACAGGAAAAGG
CCTACCTTGGCTTCTCCTGGACCTCATGAGAAATCAAAGTCTCTGGGTTCTGGGGGGAGTATGGTCGCAAGGCTGAAACT
TAAAGGAATTGACGGAGGGGCACCACCAGGCGTGGAGCCTGCGGCTTAATTTGACTCAACACGGGAAACTCACCAGGTC
CAGACATGGGAAGGATTGACAGATTGATAGCTCTTTCTTGATTCTATGGGTGGTGGTGCATGGCCGTTCTTAGTTGGTGG
AGTGATTTGTCTGGTTAATTTCGATAACGAACGAGACCTTGGCCTGCTAAATAGGGTCGGTGACCTCGGTTACCGTATCA
CTTCTTAGAGGGACATTGCGTGTCTAACGCAAGGAAGTTTGAGGCAATAACAGGTCTGTGATGCCCTTAGATGTTCTGGG
CTGCACGCGCGCTACACTGATGCATGCAACGAGTTTTCACCTTGTCCGATGGGGCTGGGTAATCTTGTGAGGGTGCATCG
TGATGGGGATAGATTATTGCAATTATTAGTCTTCAACGAGGAATGCCTAGTAGGCGCAAGTCAGCAGCTTGTGCCGATTA
CGTCCCTGCCTCTTGTACGCACCGCCCGTCGCTGCAACCGATCGGAGGGTCCTGTAAATTCATCGGACTGACCAGCCCCA
ACTTGGGGCTGGTCGGAAAGTTGCGTAAATAGAGCCCTCTAAAGGATGCAAAAGTCGTAACACGGTTT
>GQ260091 - Taenia solium 18S ribosomal RNA gene, complete sequence
TCCTGCCAGTAGTCATATGCTTGTCTCAAAGATTAAGCCATGCATGTCTCAGTTCAGGCCCTAGTACGGTGAAACCGCGA
ATGGCTCATTAAATCAGCTATGGTTTATTGGATCGTACCCGTTAAATGGATAACTGTAATAACTCTAGAGCTAATACATG
CCTCGATGCCCTGACTCTGTCGGCTTGCTGCTTGCCTGTGGGTAGGTGGTGGCAGGCAGGGTGTGGGTGCACTTATTAGA
TCAGAAGCCAACCAACTGCTCGTGCGTTGCGTGTGCAGCCGACTGCTGTAGGCGTGCGTGCGTGCGTGCGTGCGAGTGTT
GAGAGGAGACCGCTTCTGGTGACTCTGGATAATTGTTACAGATCGCAGTCGGCCTTGAGTCGGCGACGGGTCCTTCAAAT
GTCTGCCCTATCAACTGTCGATGGTAGGTGACCTGCCTACCATGGTGATAACGGGTAACGGGGAATCAGGGTTCGATTCC
GGAGAGGGAGCCTGAGAAACGGCTACCACTTCCAAGGGAGGCAGCAGGCGCGCAAATTACCCACTCCCGGCACGGGGAGG
TGGTGACGAAAAATACCGATGCGGGACTCCCTCAAGAGGCTCCGTAATCGGAATGAGTGGACTCTAAATCCTTTCACGAG
GATCAATTGGAGGGCAAGTCTGGTGCCAGCAGCCGCGGTAACTCCAGCTCCAATAGCGTATATTAAAGTTGCTGCAGTTA
AAAAGCTCGTAGTTGGATCTCGGTAGCATTGTTGCCCGCTGGTAATTGAGCGGCCGGTGTGTGGTCAACGGTGCGCGGTC
GCGCTGGCACACAGTCTGCGTGTGAGCGTGTGGTGAGCGCGTGTCGGTTGGTAGGTTGGCAGGCGAGCTGGTTGGCCGTT
GTCGCCGCTGCTGCCGCCACTGTCGGGTAGTTGGCTGCCTGGCTAAGCGGTTGGTTGGTTGGTTGGTTGGCGGGACGGCT
GAGTGGTTTGCTTACCAACTGGGCTGGCTGGTGCGATGCCCCACTGCAACTGCCTGCGTGCAGTGTGTGTTGGCAGGGAT
TGTGTTGCCGTGGCCATGAAGAAGCACTGTCGTGTCAAGCTGGCAGGGCGATGGTGTCACCTTTAAGCCATGTCTGTGGT
GTAGCAGCTGCAGGTGTGGACGGGTGTTGGGCGGTGCTCTGCACACGTTGCGGAGTCTGTCGGCTCGTCTGCATGCCTTT
GGATGCCCTTGAAAAGGTGTCCGCGGGCGGATGGCACGTTTACTTTGAACAAATTTGAGTGCTCAAATCAGGCCGATGTT
GCCTGAAAAGTTTTGCATGGAATAATGGAATAGGACTTCGGTTCTATTTCGTTGGTTTTCGGATCCGAAGTAATGATCAA
AAGAGACAGGCGGGGACGTTTGTATGGCTGCGCTAGAGGTGAAATTCATGGACCGTAGCCAGACAAACTAAAGCGAAAGC
ATTCGTCAAGCATGTTTTCATTGGCCATGAGCGAAAGTCAGAGGCTCGAAGACGATCAGATACCGTCCTAGTTCTGACCA
TAAACGATGCCAACTGACGATCCGTGGTGGTAGTAATGTTACCTTCCCCACGGGCAGTCCCCGGGAAACCTTTAAGTGTT
TGGGTTCCGGGGGAAGTATGGTTGCAAAGCTGAAACTTAAAGGAATTGACGGAAGGGCACCACCAGGAGTGGAGCCTGCG
GCTTAATTTGACTCAACACGGAAAACTCACCCGGGCCGGACACTATGAGGATTGACAGATTGAGAGCTCTTTCTTGATT
TGGTGGTTGGTGGTGCATGGCCGTTCTTAGTTGGTGGAGCGATTTGTCTGGTTAATTCCGATAACGAACGAGACTCCAGC
CTGCTAATTAGTGCGTTTGTCCACTGCACCTGCGTAGGTGACGTCGGATGACACCACCTGTCACGAGGGCGTGGCTTCCA
CTCTTGCCTCTGCTTTTCCGCTCAAATGCCCCCCTAGCGCCTCGTCCTCGTCCTCCTCGTCCTCCTCCTCGTCCTCCTCC
TCCTCCTCCTTCTTCAGCGCCTCTCCCGGCTGGCTGGCTGGGCGAGGTGGTGGTGGTGGTGGTGGCGGTGAGGATGATGG
TGAGGTTGGAGGCGGTGCAGGTGGGCGAGTGGATTGTGGCGGAGGTGAGGGATGGAGTCCGGCGTTTGTGGCCGGTTAGT
CTGGCGTTGGCCTGCGGGTGTGGCGCGAATGCCTACTTCTTAGAGGGACAAGCGGGAGAAGCCGCACGAAATAGAGCAAT
AACAGGTCTGTGATGCCCTTAGATGTCCGGGGCCGCACGCGCGCTACAATGACGGTGTCAGCGAGTCAGACCTTCTGGCC
CGAAAGGGTTGGGCAAACTGGTCAATCACCGTCGTGACAGGGATCGGGGCTTGGAATTGTTCCCCGTGAACGAGGAATTC
CTAGTAAGTGCAAGTCATAAGCTTGCGCTGATTACGTCCCTGCCCTTTGTACACACCGCCCGTCGCTACTACCGATTGAA
TGGTTTAGTAAGGTCCTTGGATTGGCGTCATTGCGGCGGTGGCCGAGAGGTCACAGCCCGACGGGCGCTGAGAAGACGAC
CGAACTTGATCATTTAGAGGAAGTAAAAGTCGTAACAAG
>JQ609338 - Taenia saginata isolate Dali 18S ribosomal RNA gene, complete sequence
TCCTGCCAGTAGTCATATGCTTGTCTCAAAGATTAAGCCATGCATGTCTCAGTTCAGGCCCTAGTACGGTGAAACCGCGA
ATGGCTCATTAAATCAGCTATGGTTTATTGGATCGTACCCGTTAAATGGATAACTGTAATAACTCTAGAGCTAATACATG
CCCCGATGCCCTGACTCTGTTAGCCTGCTGCTGCTTGCCTGTGTGTGGGTGGTGGCGGGTAGGCAGGGTGTGGGTGCACT
TATTAGATCAGAAGCCAACCAACTGCTCGAACGTTGCGTGTGCAGTCCACTGCTGTAGGCGTGCGTGCGGGTGTTGAGAG
GAGACCGCTTCTGGTGACTCTGGATAATTGTTACAGATCGCAGTCGGCCTTGAGTCGGCGACGGGTCCTTCAAATGTCTG
CCCTATCAACTGTCGATGGTAGGTGACCTGCCTACCATGGTGATAACGGGTAACGGGGAATCAGGGTTCGATTCCGGAGA
GGGAGCCTGAGAAACGGCTACCACTTCCAAGGGAGGCAGCAGGCGCGCAAATTACCCATTCCCGGCACGGGGAGGTGGTG
ACGAAAAATACCGATGCGGGACTCCCTCAAGAGGCTCCGTAATCGGAATGAGTGGACTCTAAATCCTTTCACGGGGATCA
ATTGGAGGGCAAGTCTGGTGCCAGCAGCCGCGGTAACTCCAGCTCCAATAGCGTATATTAAAGTTGCTGCAGTTAAAAAG
CTCGTAGTTGGATCTCGGTAGCATTGTTGCCTGCTGGTAACTGAGCGGTCGGTGTGTGGTCAACGGTGCGCGGTTGCGTC
AGCGCACGGTCTGCGTGCGGGCGTGTGGTGAGCGCGGGTCGGCTGGTGGGTTGGCAGGGGAGCTGGTTGGTCGTTGTCGC
CACTGCTGCTGGCATTGTCAGGTAGTTGGCCGGCTGGCTGGCTGGCTGGCTGGCTGGCTGGCTAGGCGGTTGGTTGGCGG
GACGGCTAGGTGGTTTACTTGCTGGCTGGGCTGGCTGGTGCGATGCTGCACTACTACTGCTTGCGTGCAGTGAGTGTGCG
TTGGCAGGGATTGTGTTGCCGTGGCCATGAAGAAGCACTGTCGTGTCAAGCCGGCAAGGCGACGGTGTCACCTTTGAGCC
ATGTCTGTGGTGTAACAGCTGCAGGTGTGGATGGGTGTTGGGCAGCGCTCTGCACACGCTGTGGAGTCTGTCGGCTCGTC
TGCATGTCTTTGGATGCCCTTAAAAAGGTGTCTGCGGGCGGATGGCACGTTTACTTTGAACAAATTTGAGTGCTCAAATC
AGGCCGATGTTGCCTGAAAAGTTTCGCATGGAATAATGGAATAGGACTTCGGCTCTATTTCGTTGGTTTTCGGATCCGAG
GTAATGATCAAAAGAGACGGGCGGGGACGTTTGTATGGCTGCGCTAGAGTGAAATTCATGGACCGTAGCCAGACAGACTA
AAGCGAAAGCATTCGGTCAAGCATGTTTTCATTGGCCATGAGCGAAAGTCAGAGGCTCGAAGACGATCAGATACCGTCCT
AGTTCTGACCATAAACGATGCCAACTGACGATCCGTGGTGGTAGTAGTGTTACCTTCCCCACGGGCAGTCCCCGGGAAAC
CTTTAAGTGTTTGGGTTCCGGGGGAAGTATGGTTGCAAAGCTGAAACTTAAAGGAATTGACGGAAGGGCACCACCAGGAG
TGGAGCCTGCGGCTTAATTTGACTCAACACGGGAAAACTCACCCGGGCCGGACACTGTGAGGATTGACAGATTGAGAGCT
CTTTCTTGATTTGGTGGTTGGTGGTGCATGGCCGTTCTTAGTTGGTGGAGCGATTTGTCTGGTTAATTCCGATAACGAAC
GAGGCTCCAGCCTGCTAATTAGTGCGTTTGTCCACTGCGCCTGCGTAGGTGACGCTGGATGACACCACCTGTCACGAGGA
CGTGGCAGCGGCTCTCCTCTCCGCTTCTCCGCTCGAGTGCTCTCCAGCGCCTCCTCCTCCTGCTTCTCCTCCACCTCCTC

CTCCTCCTTCTTCAGCGCCTCATCTAGCTGGCTGGCTGGCTGGCTGGCCGGGTGAGGTGGTGGTGGTGGCGGTGGTG
GTGGTAGCGGTAGGGTGGGGATGGTGCGGGTGGGCGAGTGGATTGTGGTGGAGGTGTGGGGTTGGGTGCGGCGCTCGTGG
CCGGTTAGTCTGGCGTTGGCCTCCGGGTGTGGCGCGAATGCCTACTTCTTAGAGGGACAAGCGGGAGAAGCCGCACGAAA
TAGAGCAATAACAGGTCTGTGATGCCCTTAGATGTCCGGGGCCGCACGCGCGCTACAATGACGGTGTCAGCGAGTCAGGC
CTTCTGGCCCGAAAGGGTTGGGCAAACTGGTCAATCACCGTCGTGACAGGGATCAGGGCTTGGAATTGTTCCCCGTGAAC
GAGGAATTCCTAGTAAGTGCAAGTCATAAGCTTGCGCTGATTACGTCCCTGCCCTTTGTACACACCGCCCGTCGCTACTA
CCGATTGAATGGTTTGGTAAGGTCCTCGGATTGGCGTCATTGCGGCGGTGGCCGAGAGGTCATAGCCCGACGGGCGCTGA
GAAGACGACCGAACTTGATCATTTAGAGGAAGTAAAAGTCGTAACAAG
>GQ260088 – Taenia asiatica 18S ribosomal RNA gene, complete sequence
TCCTGCCAGTAGTCATATGCTTGTCTCAAAGATTAAGCCATGCATGTCTCAGTTCAGGCCCTAGTACGGTGAAACCGCGA
ATGGCTCATTAAATCAGCTATGGTTTATTGGATCGTACCCGTTAAATGGATAACTGTAATAACTCTAGAGCTAATACATG
CCTCGATGCCCTGACTCTGTTAGCCTGCTGCTGCTTGCCTGTGTGTGGGTGGTGGCAGGTAGGCAGGGTGTGGGTGCACT
TATTAGATCGGAAGCCAACCAACTGCTCGAACGTTGCGTGTGCAGTCCACTGCTGTAGGCGTGCGTGCGGGTGTTGAGAG
GAGACCGCTTCTGGTGACTCTGGATAATTGTTACAGATCGCAGTCGGCCTTGAGTCGGCGACGGGTCCTTCAAATGTCTG
CCCTATCAACTGTCGATGGTAGGTGACCTGCCTACCATGGTGATAACGGGTAACGGGGAATCAGGGTTCGATTCCGGAGA
GGGAGCCTGAGAAACGGCTACCACTTCCAAGGGAGGCAGCAGGCGCGCAAATTACCCATTCCCGGCACGGGGAGGTGGTG
ACGAAAAATACCGATGCGGGACTCCCTCAAGAGGCTCCGTAATCGGAATGAGTGGACTCTAAATCCTTTCACGAGGATCA
ATTGGAGGGCAAGTCTGGTGCCAGCAGCCGCGGTAACTCCAGCTCCAATAGCGTATATTAAAGTTGCTGCAGTTAAAAAG
CTCGTAGTTGGATCTCGGTAGCATTGTTGCCTGCTGGTAACTGAGCGGTCGGTGTGTGGTCAACGGTGCGCGGTTGCGTC
AGCGCACGGTCTGCGTGCGGGCGTGTGGTGAGCGCGGGTCGGCTGGTGGGTTGGCAGGGGAGCTGGTTGGTCGTTGTCGC
CGCTGCTGCTGGCATTGTCAGGTAGTTGGCCGGCTGGCTGGCTGGCTGGCTGGCTGGCTGGCTAGGCGGTTGGTTGGCGG
GACGGCTGGGTGGTTTACTTGCTGGCTGGGCTGGCTGGTGCGATGCTGCACTACTACTGCTTGCGTGCAGTGAGTGTGCG
TTGGCAGGGATTGTGTTGCCGTGGCCATGAAGAAGCATTGTCGTGTCAAGCTGGCAAGGCGACGGTGTCACCTTTGAGCC
ATGTCTGTGGTGTAACAGCTGCAGGTGTGGATGGGTGTTGGGCAGCGCTCTGCACACGCTGTGGAGTCTGTCGGCTCGTC
TGCATGCCTTTGGATGCCCTTAAAAAGGTGTCTGCGGGCGGATGGCACGTTTACTTTGAACAAATTTGAGTGCTCAAATC
AGGCCGATGTTGCCTGAAAAGTTTTGCATGGAATAATGGAATAGGACTTCGGCTCTATTTCGTTGGTTTTCGGATCCGAA
GTAATGATCAAAAGAGACGGGCGGGGACGTTTGTATGGCTGCGCTAGAGGTGAAATTCATGGACCGTAGCCAGACAGACT
AAAGCGAAAGCATTCGTCAAGCATGTTTTCATTGGCCATGAGCGAAAGTCAGAGGCTCGAAGACGATCAGATACCGTCCT
AGTTCTGACCATAAACGATGCCAACTGACGATCCGTGGTGGTAGTAGTGTTACCTTCCCCACGGGCAGTCCCCGGGAAAC
CTTTAAGTGTTTGGGTTCCGGGGGAAGTATGGTTGCAAAGCTGAAACTTAAAGGAATTGACGGAAGGGCACCACCAGGAG
TGGAGCCTGCGGCTTAATTTGACTCAACACGGGAAAACTCACCCGGGCCGGACACTGTGAGGATTGACAGATTGAGAGTT
CTTTCTTGATTTGGTGGTTGGTGGTGCATGGCCGTTCTTAGTTGGTGGAGCGATTTGTCTGGTTAATTCCGATAACGAAC
GAGACTCCAGCCTGCTAATTAGTGCGTTTGTCCACTGCGCCTGCGTAGGTGACGCTGGATGGCACCACCTGCCACGAGGA
CGTGGCAGCGGCTCTTCTCTCCGCTTCTCCGCTCGAGTGCTCTCCAGCGCCTCCTCCTCCTGCTTCTCCTCCTCCTCCTT
CTTCAGCGCCTCATCTGGCTGGCTGGCTGGCTGGCCGGGTGAGGTGGCGGTGGTGGTGGTAGCGGTGGGGTGGGGATGGT
GCGGGTGGGCGAGTGGATTGTGGTGGAGGTGTGGGGTTGGGTGCGGCGCTCGTGGCCGGTTAGTCTGGCGTTGGCCTCCG
GGTGTGGCGCGAATGCCTACTTCTTAGAGGGACAAGCGGGAGAAGCCGCACGAAATAGAGCAATAACAGGTCTGTGATGC
CCTTAGATGTCCGGGGCCGCACGCGCGCTACAATGACGGTGTCAGCGAGTCAGGCCTTCTGGCCCGAAAGGGTTGGGCAA
ACTGGTCAATCACCGTCGTGACAGGGATCGGGGCTTGGAATTGTTCCCCGTGAACGAGGAATTCCTAGTAAGTGCAAGTC
ATAAGCTTGCGCTGATTACGTCCCTGCCCTTTGTACACACCGCCCGTCGCTACTACCGATTGAATGGTTTAGTAAGGTCC
TCGGATTGGCGTCATTGCGGCGGCGGCCGAGAGGTCATAGCCCGACGGGCGCTGAGAAGACGACCGAACTTGATCATTTA
GAGGAAGTAAAAGTCGTAACAAG
>DQ925309 – Diphyllobothrium latum 18S ribosomal RNA gene, complete sequence
ACCTGGTTGATCCTGCCAGTAGTCATATGCTTGTCTCAAAGATTAAGCCATGCATGTCTAAGTGCACGCCTTCATACGGT
GAAACCGCGAATGGCTCATTAAATCAGCTATGGTTTATTGGATCATACCCGTTAAATGGATAACTGTAATAACTCTAGAG
CTAATACATGCCCCGAAGCCCTGACCCGCGAGGGAATGGGTGCACTTATTAGATCAGAAGCCAACCAGGTAGTGTCCTCT
CGCTTTCGGGTGGGAGCTCCGCTGCCTGTCGTCCTTCTGGTGACTCTGGATAATTGTTACAGATCGCAGTCGGCCTTGCG
TCGGCGACGGGTCCTTCAAATGTCTGCCCTATCAACTTTCGATGGTAGGTGACCTGCCTACCATGGTGATAACGGGTAAC
GGGGAATCAGGGTTCGATTCCGGAGAGGGAGCCTGAGAAACGGCTACCACTTCCAAGGGAGGCAGCAGGCGCGCAAATTA
CCCACTCCCAGTACGGGGAGGTGGTGACGAAAAATACCGATGCGGGACTCTTATTGAGGCTCCGTAATCGGAATGAGTGA
ACTCTAAATCCTTTCACGAGGATCAATTGGAGGGCAAGTCTGGTGCCAGCAGCCGCGGTAACTCCAGCTCCAATAGCGTA
TATTAAAGTTGCTGCAGTTAAAAAGCTCGTAGTTGGATCTCGGTATCACTGTCGCCTGCCAGTCCTCGGCTGATTGTGTC
TGTGTGTGTAGGTGGGACCGTGCTCTGCGCGGCACCCCTCGCCCAGGCGCGGTTATGCTCGAGTTGGCGTGGTGGTGGTG
TCACCTTTCAGCCATGTCTGTGGTGAAATACCCGCAGGTGTAGGCGAGTGTCAGGCGGTGCTCTGCGACAGTAGGGTCCG
TCGGCTCGTTTGCATGCCTCTGGATGCCCTTCAAAAGGTGTCTATGGGCGGATGGCACGTTTACTTTGAACAAATTTGAG
TGCTCAAACCAGGCCGATGTTGCCTGAAAAGTTTTGCATGGAATAATGGAATAGGACTTCGGTTCTATTTCGTTGGTTTT
CGGATCCGAAGTAATGATCAAAAGAGACAGGCGGGGACGTTTGTATGGTTGCGCTAGAGGTGAAATTCATGGACCGTAAC
CAGACAAACTAAAGCGAAAGCATTCGTCAAGCATGTTTTCATTGGCCATGAGCGAAAGTCAGAGGCTCGAAGACGATCAG
ATACCGTCCTAGTTCTGACCATAAACGATGCCAACTGACGATCCGTGGTGGTAGTATTTTAACCATCCCCACGGGCAGTC
CCCGGGAAACCTTTAAGTCTTTGGGTTCCGGGGGAAGTATGGTTGCAAAGCTGAAACTTAAAGGAATTGACGGAAGGGCA
CCACCAGGAGTGGAGCCTGCGGCTTAATTTGACTCAACACGGGAAAACTCACCCGGCCCGGACACTGTGAGGATTGACAG
ATTGATAGCTCTTTCTTGATTTGGTGGTTGGTGGTGCATGGCCGTTCTTAGTTGGTGGAGCGATTTGTCTGGTTAATTCC
GATAACGAACGAGACTCTAGCCTGCTAATTAGTACGCCTGTCCTCTGTTCCTGTGCTGGCGGTGCTTTGGCGAAACTGCT
CACATATGTCGACTGCTATTGGGACGCGTTCTTGCGTATGCCGCGAGCTCCTCGTGGGCGAGTGGTGTGCGTAGGGGCGT
GAACTGTGGTAGGAGGCGTGTGCGGTGGGTCCGGTGCTTGCCTCCGGGTACGGCGCAGGTGTGTCTACTTCTTAGAGGGA
CAAGCATTACGAATGTGTACGAAATTGAGCAATAACAGGTCTGTGATGCCCTTAGATGTCCGGGGCCGCACGCGCGCTAC
AATGACGGTGCCAACGAGTCTGACCTTCTGGCCCGAAAGGGTTGGGCAAACTGGTCAATCACCGTCATGACAGGGATCGG
GGCTTGGAATTGTTCCCCGTGAACGAGGAATTCCTAGTAAGTGCAAGTCATAAGCTTGCGCTGATTACGTCCCTGCCCTT
TGTACACACCGCCCGTCGCTACTACCGATTGAATGGTTTAGTAAGGTCCTCGGATTGGCGCCATTGCAGTATCAGCCGCA
AGGTTGGTGCTCGACAGGTGCCGAGAAGACGACCAAACTTGATCATTTAGAGGAAGTAAAAGTCGTAACAAGGTTTCCGT
AGGTGAACCAGCGGAAG
>EU344798 – Ancylostoma duodenale 18S ribosomal RNA gene, partial sequence
ACATGCAAAGATTAAGCCATGCAAAGATTAAGCCATGCAAAGATTAAGCCATGCAAAGATTAAGCCATGCAAAGATTAAG

CGATGCAAAGATTAGGCCATGCAAAGGGTAAGCCATGCAAAGATTATCACATGCCTTCGGGTGTAGTTTGCTGATTCTAA
ATAACGCTGCATATCGGCGGCTTGTCCGCCGATATTCCGAAAAAGTGTCTGCCCTATCAACCTGATGGTAGTCTATTAGT
CTACCATGGTTATTACGGGTAACGGAGAATAAGGGTTCGACTCCGGAGAGGGAGCCTTAGAAACGGCTACCACATCCAAG
GAAGGCAGCAGGCGCGTAACTTATCCACTCTTGAAGAGATGAGATAGTGACTAAAAATAAAAAGACCATTCCTATGGAAC
GGTCATTTCAATGAGTTGATCATAAACCTTTTTTCGAGGATCAAGTGGAGGGCAAGTCTGGTGCCAGCAGCCGCGGTAAT
TCCAGCTCCACTAGTGTAAATCGTCATTGCTGCGGTTAAAAAGCTCGTAGTTGGATCTGAGTCGCATGCAGTGGTTCGCC
TTTGGCGTTAATCGCTGTTGCGACTATTTGCTGGTTTTCTACTGAAGTTTCGGCTTCTTTAGTGGCTAGCGAGTTTACTT
TGAATAAATTAGAGTGCTCAGAACAAGCGTTTGCTTGAATGCTCGATCATGGAATAATAAAAGAGGACTTCGGTTCTATT
TATTGGTTCAGGAACTGAAATAATGGTTAAGAGGGACAATTCGGGGGCATTCGTATCCCTGCGCGAGAGGTGAAATTCGT
GGACCGCAGGGGGACGCCCTAAAGCGAAAGCATTTGCCAAGAATGTCTTCATTAATCAAGAACGAAAGTCAGAGGTTCGA
AAGCGATTAGATACCGCCCTAGTTCTGACCGTAAACTATGCCATCTAGCGATCCGATGGGGTATTGTTGCCTTGTCGAGG
AGCTTCCCGGAAACGAAAGTCTTTCGGTTCCTGGGGTAGTATGGTTGCAAAGCTGAAACTTAAAGAAATTGACGGAATGG
CACCACCAGGAGTGGAGCCTGCGGCTTAATTTGACTCAACACGGGAAAACTCACCCGGCCCGGACACCGTAAGGATTGAC
AGATTGAAAGCTCTTTCTCGATTTGGTGGTTGGTGGTGCATGGCCGTTCTTAGTTGGTGGAGCGATTTGTCTGGTTTATT
CCGATAACGAGCGAGACTCTAGCCTGCTAAATAGTGGCTGGATTTTTACGTCCAGTCTACTTCTTAGAGGGATAAGCGGT
GTTTAGCCGCACGAGATTGAGCGATAACAGGTCTGTGATGCCCTTAGATGTCCGGGGCTGCACGCGCGCTACAATGGAAG
AATCAGCTGGCCTATCCATTGCCGAAAGGCATTGGTAAACCGTTGAAACTCTTCCGTGACCGGGATAGGGAATTGTAATT
ATTTCCCTTGAACGAGGAATTCCTAGTAAGTGTGAGTCATCAGCTCACGCTGATTACGTCCCTGCCATTTGTACACACCC
CCCGTCTCTGTCCGGGAATGAGCTGTCTCGAGAGGACTTCGGCCTGCTATATCGAGTCCTTCGGGTCGCGTTAAGGCGGG
AAACAGTACGTCGCAAGGGTAG
>AJ920348 - Necator americanus 18S rRNA gene
CGCTATATGCTCAGTTTAAAGATTAAGCCATGCATGTCGAGTTCATCTAAAGAGAAACTGCGAACGGCTCATTAGAGCAG
ATGTCACTTATTCGGAAAGTCCTTTTGGATAACTGCGGCAATTCTGGAGCTAATACATGCGAATAAACCCTGACTTTCGA
AAGGGTGCAATTATTAGAGCAAATCAATCATCTTCGGATGTAGTTTGCTGACTCTAAATAACGCTGCATATCGGCGGCTT
GTCCGCCGATATTCCGAAAAAGTGTCTGCCCTATCAACCTGATGGTAGTCTATTAGTCTACCATGGTTATTACGGGTAAC
GGAGAATAAGGGTTCGACTCCGGAGAGGGAGCCTTAGAAACGGCTACCACATCCAAGGAAGGCAGCAGGCGCGTAACTTA
TCCACTCTTGAAGAGATGAGATAGTGACTAAAAATAAAAAGACCATTCCTATGGAACGGTCATTTCAATGAGTTGATCAT
AAACCTTTTTTCGAGGATCAAGTGGAGGGCAAGTCTGGTGCCAGCAGCCGCGGTAATTCCAGCTCCACTAGTGTAAATCG
TCATTGCTGCGGTTAAAAAGCTCGTAGTTGGATCTGAGTCGCATGCAGTGATTCGCCATTGGCGTTAATCGCTGTTGCGA
CTATTTGCTGGTTTTCTACTAAAGTTTCGGCTTTTTTAGTGGCTAGCGAGTTTACTTTGAATAAATTAGAGTGCTCAGAA
CAAGCGTTTGCTTGAATGCTCGATCATGGAATAATAAAAGAGGACTTCGGTTCTATTTATTGGTTCAGGAACTGAAATAA
TGGTTAAGAGGGACAATTCGGGGGCATTCGTATCCCTGCGCGAGAGGTGAAATTCGTGGACCGCAGGGGGACGCCCTAAA
GCGAAAGCATTTGCCAAGAATGTCTTCATTAATCAAGAACGAAAGTCAGAGGTTCGAAGGCGATTAGATACCGCCCTAGT
TCTGACCGTAAACTATGCCATCTAGCGATCCGATGGGGTATTGTTGCCTTGTCGAGGAGCTTCCCGGAAACGAAAGTCTT
TCGGTTCCTGGGGTAGTATGGTTGCAAAGCTGAAACTTAAAGAAATTGACGGAATGGCACCACCAGGAGTGGAGCCTGCG
GCTTAATTTGACTCAACACGGGAAAACTCACCCGGCCCGGACACCGTAAGGATTGACAGATTGAAAGCTCTTTCTCGATT
TGGTGGTTGGTGGTGCATGGCCGTTCTTAGTTGGTGGAGCGATTTGTCTGGTTTATTCCGATAACGAGCGAGACTCTAGC
CTGCTAAATAGTGGCTGGATTTTTACGTCCAGTCTACTTCTTAGAGGGATAAGCGGTGTTTAGCCGCACGAGATTGAGCG
ATAACAGGTCTGTGATGCCCTTAGATGTCCGGGGCTGCACGCGCGCTACAATGGAAGAATCAGCTGGCCTATCCATTGCC
GAAAGGCATTGGTAAACCGTTGAAACTCTTCCGTGACCGGGATAGGGAATTGTAATTATTTCCCTTGAACGAGGAATTCC
TAGTAAGTGTGAGTCATCAGCTCACGCTGATTACGTCCCTGCCATTTGTACACACCGCCCGTCGCTGTCCGGGACTGAGC
TGTCTCGAGAGGACTGCGGACTGCTGTCGCGAGGCCTTCGGGTCGCGTTATGGCGGGAAACAGTTCAATCGCAATGGCTT
GAACCGGGTAAAAGTCGTAACAAGGTATCTGTAGGTGAACCTGCAGATGGATCATCG
>AJ287525 - Hymenolepis microstoma 18S rRNA gene
ATCCTGCCAGTAGTCATATGCTTGTCTCAAAGATTAAGCCATGCATGTCTAAGTTCATGCCTTTATACGGTGAAACCGCG
AATGGCTCATTAAATCAGCTATGGTTTATTGGATCGTACTCGTTAAATGGATAACTGTAATAACTCTAGAGCTAATACAT
GCCACGAAGCCCTGACCCCGCTCGTCGGGGAATGGGTGCACTTATTAGAACAGAAGCCAACCAGTTTATGCATTCCTTCG
GGGGTGTGTGAGCTGTAGCCCTTCTGGTGACTCTGGATAATTGTTACAGATCGCAGTCGGCTTTACGTCGGCGACGGGTC
CTTCAAATGTCTGCCCTATCAACTTTCGATGGTAGGTGACCTGCCTACCATGGTGATAACGGGTAACGGGGAATCAGGGT
TCGATTCCGGAGAGGGAGCCTGAGAAACGGCTACCACTTCCAAGGGAGGCAGCAGGCGCGCAAATTACCCACTCCCGGTA
CGGGGAGGTGGTGACGAAAAATACCGATGCGGGACTCATTAACGAGGCTCCGTAATCGGAATGAGTGGACTCTAAATCCT
TTCACGAGGATCAATTGGAGGGCAAGTCTGGTGCCAGCAGCCGCGGTAACTCCAGCTCCAATAGCGTATATTAAAGTTGC
TGCAGTTAAAAAGCTCGTAGTTGGATCTCGGTAGCATTGTTGCCTGCTCAGTTGTCATGCTCTGCGGCGTGCATCATATC
GTGTTTGTTTGCATTATCCACGCTCTCTCCTGAGGGCCGGGTGTGTGGGCATCGCGGTGGGTGTACACTAAAACCGTAGT
GTATTGAGCTGGCGTGGCGATGGTGTCACCTTTGAGCCATGTCTGTGGTCTAACAGCCACAGGTGTAATCGGGTGTCGGG
CAGTGCTCTGCTCCTTTTGTGGAGCCTAGTCGGCTCGGTTGCATGCCAATGAATGCCCTTCAAAAGGTGTTCATGGGCGG
ATGGCACGTTTACTTTGAACAAATTTGAGTGCTCAAATCAGGCCGACGTTGCCTGAAAAGTTTTGCATGGAATAATGGAA
TAGGACTTCGGTTCTATTTCGTTGGTTTTCGGATCCGAAGTAATGATCAAAAGAGACAGGCGGGGACGTTTGTATGGTTG
CGTTAGAGGTGAAATTCATGGCACCGTAGCCAGACAAACTAAAGCGAAAGCATTCGTCAAGCATGTTTTCATTGGCCATG
AGCGAAAGTCAGAGGCTCGAAGACGATCAGATACCGTCCTAGTTCTGACCATAAACGATGCCAACTGACGATCCGTGGCG
GTAGTTTATAAACCTTCCCCACGGGCAGTCCCCGGGAAACCTTTAAGTCTTTGGGTTCCGGGGGAAGTATGGTTGCAAAG
CTGAAACTTAAAGGAATTTACGGAAGGGCACCACCAGGAGTGGAGCCTGCGGCTTAATTTGACTCAACACGGGAAAACTC
ACCCGGGCCGGACACTATGAGGATTGACAGATTGATAGCTCTTTCTTGATTTGGTGGTTGGTGGTGCATGGCCGTTCTTA
GTTGGTGGAGCGATTTGTCTGGTTAATTCCGATAACGAACGAGACTCCAGCCTGCTAATTAGTGCATTTGTCCACTGTAC
CAGCCGAGCGGCGCAGTGTCAACTGCTTTGGCTCAGTGTGGGATCTGGTGCGTTTGCGCATCCTGCGCGGTCGTGCTGCC
TTGTGTGGTGTGGCTGTGTGGGGCTGTGTGCGGCGTGCTTGGCCTGCATGTTGTGCTCGGGCGGATGGCACCACGTTAGC
TAGCAGGTGCGGCGCGAATGCTTACTTCTTAGAGGGACACGCGGGAGAAGCCGCACGAAATAGAGCAATAACAGGTCTGT
GATGCCCTTAGATGTCCGGGGCCGCACGCGCGCTACAATGGCGGTGTCAACGAGTCAGACCTTCTGGCCTGAAAAGGTTG
GGTAAACTGGTCAATCACCGTCATGACAGGGATCGGGGCTTGGAATTGTTCCCCGTGAACGAGGAATTCCTAGTAAGTGC
AAGTCATAAGCTTGCGCTGATTACGTCCCTGCCCTTTGTACACACCGCCCGTCGCTACTACCGATTGAATGGTTTAGTAA
GGTCCTTGGATTGGTGCCATTTAGGTTCCACCGAAAGGTGAGTATCTAGCCGGTGCTGAG
>DQ094173 - Loa loa small subunit ribosomal RNA gene, partial sequence
GCTTGTCTCAAAGATTAAGCCATGCATGTCTAAGTTCAAATAAACCTATAATGGTGAAACCGCGAACGGCTCATTATAAC

AGCTATAATGTACTTGATGTTGATTATCCAACGTGGATAACTGTGGCAATTCTAGAGCTAATACATGCACCAAAGCTCCG
ACTTTTGAACGAGCGCATCTATTAGATTAAAACCAATCGGGTTATTAGCCCGTTAATTGGTGACTCTGAATAGCTATGGC
TGATCGCATGGTCTTGTACCGGCGACGTATCTATCAAGTGTCTGCCTTATCAACTTTCGATGGTAGTTTATGTGCCTACC
ATGGTTGTAACGGGTAACGGAGAATAAGGGTTCGACTCCGGAGAGGGAGCCTGAGAAACGGCTACCACATCCAAGGAAGG
CAGCAGGCGCGCAAATTACCCACTCTCAGAATGAGGAGGTAGTGACGAAAAATAACGAGACCGTTCTCTTTGAGGCCGGT
TATCGGAATGGGTACAATTTAAACCCGTTAACGAGGATCTATGAGAGGGCAAGTCTGGTGCCAGCAGCCGCGGTAATTCC
AGCTCTCAAAGTGTATATCGTCATTGCTGCGGTTAAAAAGCTCGTAGTTGGATCTGCGTCTCAGGACCTGGTCCATCCAT
TGGATGAGAACTGGGCTCCTAGGCTAATATTGCCAGTTTTCCCTACGTTACCTTGATCGGTTGCGTATGGTGGCTAGCAA
GTTTACCTTGAAAAAATTAGAGTGCTCAATGCGGGCTAATGCCTGAATACTCGTGCATGGAATAATGAAATAGGATCTCG
GTTCTATTTTGTTGGTTTTCTGATCTGAGATAATGGTTAAGAGGGACGGACGGGGGCATTCGTATCGCTGCGTGAGAGGT
GAAATTCTTGGACCGTAGCGAGACGTACGACTGCGAAAGCATTTGCCAAGAATGTCTTCATTAATCAAGAACGAAAGTCA
GAGGTTCGAAGGCGATCAGATACCGCCCTAGTTCTGACCGTAAACGATACCAACTAGCGTTCCGTCGGCGGTAAATACGC
CTTGACGGGCAGCTTCCCGGAAACGAAAGTTTTTCGGTTCCGGGGGAAGTATGGTTGCAAAGCTGAAACTTAAAGAAATT
GACGGAAGGGCACCACCAGGAGTGGAGCCTGCGGCTTAATTTGACTCAACACGGGAAAACTCACCTGGCCCGGACACCGT
GAGGATTGACAGATTGAGAGCTCTTTCATGATTCGGTGGTTGGTGGTGCATGGCCGTTCTTAGTTGGTGGAGTGATTTGT
CTGGTTTATTCCGATAACGAGCGAGACTCTAGCCTACTAAATAGTTACTGGATAATTTTGTCCAGACAACTTCTTAGAGG
GACAAGCGGTGTTTAGCCGCATGAAGTTGAGCAATAACAGGTCTGTGATGCCCTTAGATGTCCAGGGCTGCACGCGCGCT
ACACTGGAGGAATCAGCGTGCTGTAACCATTACCGAAAGGTACTGGTAACCCCTTGAAAATCCTCCGTGATCGGGATCGG
GAATTGCAATTATTTCCCTTGAACGAGGAATTCCTAGTAAGTGTGAGTCATCAGCTCACGTTGATTACGTCCCTGCCCTT
TGTACACACCGCCCGTCGCTGCCCGGGACTGAGCCGTTTCGAGAAAAGCGGAGACTGCTGTTTTGATACCTTTCGAGGTG
GAGATTCTTTGGTGGAAACCGCTTTAATCGCAGTGGCTTGAACCGGGCAAAAGTCGTAACAAGGTTTCCGTAGGTGAACC
TGCAGCTGGATCA
>AF473852 - Giardia intestinalis small subunit ribosomal RNA gene, complete sequence
CATCCGGTCGATCCTGCCGGAATCCGACGCTATCCCCAAGGATACAAGCCATGCATGCCCGCACACCCCGAAGGCGGCAG
ACGGCTCAGGACGACGGTTGCACCCCCCGCGGCGGTCCCTGCTAGCCGGACACCGCTGGCAACCCGGCGCCAAGACGTGC
GCGCAAGTGCGGGCGCCCGCGGGCGAGCAGCGTGACGCAGCGACGGCCCGCCCGGGCTTCCGGGGCATCACCCCGATGGC
GCGGTTGCGGCCCGCCATAGGAGCGACGCCTGGCGGAGAATCAGGGTTCGACTCCGGAGAGCGGGCCTGAGAGACGGCCC
GCACATCCAAGGACGGCAGCAGGCGCGGAACTTGCCCAATGCGCGGCGCGCGAGGCAGCGACGGGGAGCGCACAGAGCGA
GGCGGGCCCACACGCCCCGCCGCGGAGCCGAGGGCAAGGTCTGGTGCCAGCAGCCGCGGTAATTCCAGCTCGGCGAGCGT
CGCGTGGCGCTGCTGCAGTTAAAACGCCCGTAGTTGGCCCCCCGCCGCCGCGAGGAAAAGGGAGCGCTCCAGGCAGGCCC
GTTGGACCCGCCGCGCGGGACGGCGCTGCGGGCGCGGCGCGCCGCGGCAGCCCCGAGGAGAGCGGGCGGGGGCACTGGTA
CTGGCCGGGGACGGGTGGAATAGGATGATCCCGCCAAGACCGCCGGGGGCGAAGGCGTCTGCCAAGACCGCCTCTGTCAA
TCAAGGGCGAAGGTCGGGGGCTAGAAGGCGATCAGACACCACCGTATTCCCGACCGTAAACGGTGCCGCCCCGCGGCCGG
CGCGCGTGTCCCGCCGGCCGCCCAGGGAAACCGGGAGGCTCCGGGCTCTGGGGGGAGTATGGCCGCAAGGCTGAAACTTG
AAGGCATTGACGGAGGGGTACCACCAGACGTGGAGTCTGCGGCTCAATCTGACTCAACGCGTGCACCTCACCAGGCCCGG
ACGCGCGGAGGACCGACAGCCGGGTGCGCTTTCGCGATCGCGCGGGCGGTGGTGCATGGCCGCTCCCAGCCCGTGGCGCG
AGCCGTCTGCTCCATTGCGACAACGAGCGAGACCCCAGCCGCGGATGCCGCGGGACTGCCCGCGCGGAGCGGGAGGAAGG
CGGGGCGATAGCAGGTCTGTGATGCCCTCAGACGCCCTGGGCTGCACGCGCGCTACACTGGCGGGGCCAGCCGGCGCCCG
CGAGGACGCGCGGAGCCCCCGCCGTGGCCGGGACCGCGGGCTGGAACGCCCCCGTGCACCAGGAATGTCTTGTAGGCGCC
CGCCACGACCGCGCGCCGGACGCGTCCCTGCCCCTTGTACACACCGCCCGTCGCTCCTACCGACTGGGCGCGCGGGCGAG
CGTCCCGGACCAGCGAAGGGCCGCGAGCCCCCGCGCCTGGAGGAAGGAGAAGTCGTAACAAGGTATCCGTAGGTGAACCT
GCAGAAGGATCAAGCTTGGATCCC
>KP875567 - Halicephalobus gingivalis isolate 2014-10-972 18S ribosomal RNA gene, partial sequence
GTTATTAGATTAAAACCATTGGGCTCTGCCCATATATGTTTACTCTAGATAATGCGCTACCATACAGATTAATTTCTGAT
GGAATGCCGTATTTGTATCTGCCCTATCAACTATATAACCGTGTATTGAACGGTTATGGTGTTGCCGGGTAACGGAGAAT
TAGGGTTCGTCTCCGGAGAGAATGCCTTAAAAACGGCTTTCACATCCAAGGAAGGCAGCAGGCGCGAAAATTACCCACTC
TCAGTGCGAGGAGGTAGTGACGTGAAATGACAAGTTGCATTCTTTATAGGATGGAACATTGGAATGGTTTTATTTTAAAT
CCATTAAAGATTATCAATGAGAGGGCAAGTCTGGTGCCAGCAGCCGCGGTAATTCCAGCTCTCATACTGCATTGAATTGC
TGTTGCGGTTAAAAAGTTCGTAGTTGCAAGTGTACGATGAGGAGTGGTTCGTCTTATTGACGATACTATAACTCTCGTTA
AAGGGTTATATGTTGAAAGACATATACTCTAGTTACTTTGAGTAAATCAGAGTGCTTAGAACAAGCGTATTGCTTGAATG
GTTGTGCATGGAATAACAATACAATGCATTGAGATTTGTTTTGTTGGTTTTAATGTCAATGAATGATTAAAAGGAACAAA
CGGGGGCAAAAGTATCATAAGGCGAGAGGTGAAATTCGTGGACCCTTGTGAGACTGCCTAAAGCGAAAGCATT
>AY193874 - Hymenolepis nana 18S ribosomal RNA gene, complete sequence
GGCTCATTAAATCAGCTATGGTTTATTGGATCATACTCGTTAAATGGATAACTGTAATAACTCTAGAGCTAATACATGCC
ACGAAGCCCTGACCCCGGGCTCCCTCGGGGAATGGGTGCACTTATTAGAACAGAAGCCAACCAGTCTCCGCGTGCATTCC
CTCCTTCGGGAGGTGTGTGCGCGGGCTGCAGCACTTCTGGTGACTCTGGATAATTGTTACAGATCGCAGTCGGCTTTACG
TCGGCGACGGGTCCTTCAAATGTCTGCCCTATCAACTTTCGATGGTAGGTGACCTGCCTACCATGGTGATAACGGGTAAC
GGGGAATCAGGGTTCGATTCCGGAGAGGGAGCCTGAGAAACGGCTACCACTTCCAAGGGAGGCAGCAGGCGCGCAAATTA
CCCACTCCCGGTACGGGGAGGTGGTGACGAAAAATACCGATGCGGGACTCATTTACGAGGCTCCGTAATCGGAATGAGTG
GACTCTAAATCCTTTCACGAGGATCAATTGGAGGGCAAGTCTGGTGCCAGCAGCCGCGGTAACTCCAGCTCCAATAGCGT
ATATTAAAGTTGCTGCAGTTAAAAAGCTCGTAGTTGGATCTCGGTAGCATTGTTGCCTGCTCAGTTGTCATGCTCTGCGG
CGTGCATCACATCGTGTGGGTCGGCTGTGCATTGTCCACGCTCCTATGGGGGCTGGGCGTGTGCGCTTGGCTTCATCACG
GTGGGTGTACACAAAAACCGCAGTGTATTGAGCTGGCGTGGCGATGGTGCCACCTTTGAGCCATGTCTGTGGTGTAACAG
CCACAGGTGTATGCGAGTGTCGGGCAGTGCTCTGCACCTTCTGTGGGGCCTAGCCGGCTCGCCTGCATGCCAGTGGATGC
CCTTTAAAAGGTGTTCATGGGCGGATGGCACGTTTACTTTGAACAAATTTGAGTGCTCAAATCAGGCCGATGTCGCCTGA
AAAGTTTTGCATGGAATAATGGAATAGGACTTCGGTTCTATTTCGTTGGTTTTCGGATCCGAAGTAATGATCAAAAGAGA
CAGGCGGGGACGTTTGTATGGCTGCGCTAGAGGTGAAATTCATGGACCGTAGCCAGACAAACTAAAGCGAAAGCATTCGT
CAAGCATGTTTTCATTGGCCATGAGCGAAAGTCAGAGGCTCGAAGACGATCAGATACCGTCCTAGTTCTGACCATAAACG
ATGCCAACTGACGATCCGTGGCGGTAGTTTCTAAACCTTCCCCACGGGCAGTCCCCGGGAAACCTTTAAGTCTTTGGGTT
CCGGGGGAAGTATGGTTGCAAAGCTGAAACTTAAAGGAATTGACGGAAGGGCACCACCAGGAGTGGAGCCTGCGGCTTAA
TTTGACTCAACACGGGAAAACTCACCCGGCCGGACACTGTGAGGATTGACAGATTGATAGCTCTTTCTTGATTTGGTGG
TTGGTGGTGCATGGCCGTTCTTAGTTGGTGGAGCGATTTGTCTGGTTAATTCCGATAACGAACGAGACTCCAGCCTGCTA
ATTAGTGCATTTGTCCACTGCACCAGCCGAGCGGCGCAGTGTCAACTGCTCTGGCTCAGTGCAGGAACCGGCGCGTTTGT

GCATCCTGCGTGGTCGCACTGCCTCGTGTGGTGTGGCTGTGTGGGGCTATGCGCGGCGTGTTAGGCCTGCATGTTGTGCC
TGGGGCGGATGGCACCACGTTAGCTAGCAGGTGCGGCGCGAATGCTTACTTCTTAGAGGGACACGCGGGAGAAGCCGCAC
GAAATAGAGCAATAACAGGTCTGTGATGCCCTTAGATGTCCGGGGCCGCACGCGCGCTACAATGGCGGTGTCAACGAGTC
AGACCTTCTGGCCTGAAAAGGTTGGGCAAACTGGTCAATCACCGTCATGACAGGGATCGGGGCTTGGAATTGTTCCCCGT
GAACGAGGAATTCCTAGTAAGTGCAAGTCATAAGCTTGCGCTGATTACGTCCCTGCCCTTTGTACACACCGCCCGTCGCT
ACTACCGATTGAATGGTTTAGTAAGGTCCTTGGATTGGTGCCATTTAGGTTCCGCCGAAAGGTGCGTATCTAGCCGGCGC
CGAGA
>AB544347 - Eimeria reichenowi gene for 18S ribosomal RNA, partial sequence, clone: A1_7_74
GTATAAGCTTTTATACGGTGAAACTGCGAATGGCTCATTAAAACAGTTATAGTTTATTTGATGGTCACTTTTACATGGAT
AACCATGGTAATTCTATGGCTAATACATGCGCAAAAGCCACCTTCTTTGGAGGGGCAGTGTTTATTAGATACAAAACCAA
CCCACTTTAGTGGAGCCTTGGTGATTCATAGTAACCGAACGGATCGCAATTTGGCTTTTTGGCCTGCGATAGATCATTCA
AGTTTCTGACCTATCAGCTTTCGACGGTAGGGTATTGGCCTACCGTGGCAGTGACGGGTAACGGGGAATTAGGGTTCGAT
TCCGGAGAGGGAGCCTGAGAAACGGCTACCACATCTAAGGAAGGCAGCAGGCGCGCAAATTACCCAATGAAAACAGTTTC
GAGGTAGTGACGAGAAATAACAATACAGGGCATTTAATGCTTTGTAATTGGGATGATGGGAATGTAAAACCCTTTCAGAG
TAACAATTGGAGGGCAAGTCTGGTGCCAGCAGCCGCGGTAATTCCAGCTCCAATAGCGTATATTAGAGTTGTTGCAGTTA
AAAAGCTCGTAGTTGAATTTCTGTCGGTGCTATCCGGTACCGCCTGTATGGGTGTGCACCAGGATTGGCTTCGGCATTCT
TCCGTAGCTTAATCCCGCTTAATTGCGTGGATTAGGTGTTTCGGAACTTTTACTTTGAGAAAAATAGAGTGTTTCAAGCA
GGCTTGTCGCCTTGAATACTGCAGCATGGAATAATAAGATAGGACCTTGGTTCTATTTTGTTGGTTTCTAGGACTAAGGT
AATGATTAATAGGGACAGTTGGGGGCATTCGTATTTAACTGTCAGAGGTGAAATTCTTAGATTTGTTAAAGACGAACTAC
TGCGAAAGCATTTGCCAAGGATGTTTTCATTAATCAAGAACGACAGTAGGGGGTTTGAAGACGATTAGATACCGTCGTAA
TCTCTACCATAAACTATGCCGACTAGAGATAGGGAAATGCCTACCTTGGCTTCTCCTGCACCTCATGAGAAATCAAAGTC
TCTGGGTTCTGGGGGGAGTATGGTCGCAAGGCTGAAACTTAAAGGAATTGACGGAAGGACACCACCAGGCGTGGAGCCTG
CGGCTTAATTTGACTCAACACGGGGAAACTCACCAGGTCCAGACATGGAAGGATTGACAGATTGATAGCTCTTTCTTGA
TTCTATGGGTGGTGGTGCATGGCCGTTCTTAGTTGGTGGAGTGATCTGTCTGGTTAATTTCGGTAACGAACGAGACCTTA
GCCTGCTAAATAGGATCTAGAACATTATGTTCTGGTATCACTTCTTAGAGGGACTTTGCGTGTCTAACGCAAGGAAGTTT
GAGGCAATAACAGGTCTGTGATGCCCTTAGATGTTCTGGGCTGCACGCGCGCTACACTGATGCACTCAACGAGTTCATAA
CCTTGTCCGGAAGGTCTAGGTAATCTTTTGAGTATGCATCGTGATGGGGATAGATTATTGCAATTATTAATCTTCAACGA
GGAATGCCTAGTAGGCGCAAGTCATCAGCTTGCGCCGATTGCGTCCCTGTGCTTTGTACACACCGCCCGTCGCTGCAACC
GATCGGAGGGTCCTGTGAATTCATCGGACTGACCTGTTGGCCTTTGTGCTAGCTCGTCGGGA
>KP789172 - Eimeria fulva isolate YZ 18S ribosomal RNA gene, partial sequence
GGTGAAACTGCGAATGGCTCATTAAAACAGTTATAGTTTATTTGATGGTCACTTTTACATGGATAACCATGGTAATTCTA
TGGCTAATACATGCGCAAATGCCACCTTCTCTGGAGGGGCAGTGTTTATTAGATACAAAACCAACCCACTATATTGTGGA
GTCTTGGTGATTCATAGTAACCGAACTGATCGCACTTGGCTTCGTGCCCGCGATGGATCATTCAAGTTTCTGACCTATCA
GCTTTCGACGGTAGGGTATTGGCCTACCGTGGCAGTGACGGGTAACGGGGAATTAGGGTTCGATTCCGGAGAGGGAGCCT
GAGAAACGGCTACCACATCTAAGGAAGGCAGCAGGCGCGCAAATTACCCAATGAAAACAGTTTCGAGGTAGTGACGAGAA
ATAACAATACAGGGCATTTATTGCTTTGTAATTGGAATGATGGGAATGTAAAACCCTTTCAGAGTAACAATTGGAGGGCA
AGTCTGGTGCCAGCAGCCGCGGTAATTCCAGCTCCAATAGCGTATATTAGAGTTGTTGCAGTTAAAAAGCTCGTAGTTGG
ATTTCTGTCGGTGCTGTCCTGTTCTGCCTGTATAGGTGTGAATTAGGATTGGCTTCGGCATTTTTCCGTAGCCTTATGTC
ACACTTCAGTGTGTGTCATATGGTGTTTCGGAACTTTTACTTTGAGAAAAATAGAGTGTTTCAAGCAGGCTTGTCGCCTT
GAATACTGCAGCATGGAATAATAGGATAGGACCTTGGTTCTATTTTGTTGGTTTCTAGGGCTAAGGTAATGATTAATAGG
GACAGTTGGGGGCATTCGTATTTAACTGTCAGAGGTGAAATTCTTAGATTTGTTAAAGACGAACTACTGCGAAAGCATTT
GCCAAGGATGTTTTCATTAATCAAGAACGACAGTAGGGGGTTTGAAGACGATTAGATACCGTCGTAATCTCTACCATAAA
CTATGCCGACTAGAGATAGGAAAATGCCTTTCTTGGCTTCTCCTGCACCTCATGAGAAATCAAAGTCTCTGGGTTCTGGG
GGGAGTATGGTCGCAAGGCTGAAACTTAAAGGAATTGACGGAAGGGCACCACCAGGCGTGGAGCCTGCGGCTTAATTTGA
CTCAACACGGGGAAACTCACCAGGTCCAGACATGGGAATGATTGACAGATTGATAGCTCTTTCTTGATTCTATGGGTGGT
GGTGCATGGCCGTTCTTAGTTGGTGGAGTGATCTGTCTGGTTAATTTCGATAACGAACGAGACCTTAGCCTGCTAAATAG
GGTCAGGAACATCTGTTTCTGTATCACTTCTTAGAGGGACTTTGCGTGTCTAACGCAAGGAAGTTTGAGGCAATAACAGG
TCTGTGATGCCCTTAGATGTTCTGGGCTGCACGCGCGCTACACTGATGCATGCAACGAGTTCATAACCTTGGCCGGCAGG
TCTAGGTAATCTTTTGAGTATGCATCGTGATGGGGATAGATTATTGCAATTATTAATCTTCAACGAGGAATGCCTAGTAG
GCGCAAGTCAGCAGCTTGCGCCGATTACGTCCCTGCCCTTTGTACACACCGCCCGTCGCTGCAACCGATCGGAGGGTCCT
GTGAACTCATCGGACTGATCAGTTGTGCTTCACTTCTGGTCGGAAAGTTGCGTAAATAGAGCCCTCTAAAGGATGCAAAA
GTCGTAACACGGTT
>GQ352556 - Trichuris vulpis isolate D56 clone A 18S ribosomal RNA gene, partial sequence; internal transcribed spacer 1, complete sequence; and 5.8S ribosomal RNA gene, partial sequence
ACGGTGAAGCCGCGAATGGCTCATTATAACAGCCATTGTTCGCAAGAACTGATACTCACTTGGATAACTGTGGAAATTCT
AGAGCTAATACATGCCTGAAAGCTTCGGCGCGCAATGCGGCGGAGCGCATTTATTAGTACAAAACCAATCGGGCGTTCGC
TGACGGCGTTCGCCCGCCAACGGTTGGTGAATCGGAATGACTATGCTGATCGCACGGTCCAGCACCGGCGACGAATCTTT
GAAATGACTTGCTCATCAACTTTCGATGGTACGCTACGTGCTTACCATGGTGACAACGGTTAACGGAGAATCAGGGTTCG
ACTCCGGAGAGGGAGCCTGAGAAACGGCTACCACATCCAAGGAAGGCAGCAGGCACGCAAATTACCCACTCCCAGATCGG
GGAGGTAGTGACGAAAAATAACGGAACGTATCTCCATGAGACGCGTTACCGGAACGACCGAGCCGTACACAAGCTCGGCT
AATTCTATTGGAGGGCAAGTCTGGTGCCAGCAGCCGCGGTAATTCCAGCTCCAATAGCGTATATTAAAGTTGCTGCGGTT
AAACCGCTCGTAGTTGGATTTTGGTGAACGACGACGGTCGTCCTAAGCAGGAGTCGCTCCGTCGCTCGTCACCTGTTCAA
TCAAGCTTGTCCTTGATGCTCTTGAGTGAGTGTCCTGGGCGACTTGAAAGTTTACTTTGAGAAAATGGAAGCGCTCAAGG
CAGGCCGTAGTGCTTGAACAGTGGTGCATGGAATAATGAAAGATGGCCTCGGTGCTATTTTGTTGGTTTACGGTGACGAG
GCAATGATTAAAAGAGACAGACGGGGACATTCGTATTGCTGCGTTAGAGGTGAAGTTCTTGGATCGCAGCAAGACGAACA
ATTGCGAAAGCATTTGTCAAGAATGTTTTCATTAATCAAGAACGAAAGTTAGAGGTTCGAAGGCGATCAGATACCGCCCT
AGTTGTGACCGTAAACGATGCCAACCAGCGATTCGGCGACGTTCTTCTTATGACTCGCTGAGCAGCTTCCGGGAAACCAA
AGTTTTTCGGTTCCGGGGGAAGTATGGTTGCAAAGCTGAAACTTAAAGGAATTGACGGAAGGGCACCACCAGGAGTGGAG
CATGCGGCTTAATTTGACTCAACACGGGAAAACTCACCCGTCCCGAACACTGTGAGGATTGACAGATCAAGAGCTCTTTC
TTGATTCAGTGGGTAGTGGTGCATGGCCGTTCTTAGTTGGTGGAGCGATTTGTCTGGCTAATTCCGATAACGAACGAGAC
TCTGGCCTACTAACTAGCGGCGGTGTTCATGCCTCCTGACGGGGGCCGCGTGCGGCAACCGCCGGGCGCGCCCCTTGGAG
CAGCAGCGCCGGCAGCCGCTTCTTAGAGGGACCAGCGACACTTTCGCAAGCCGCACGAGAAAGAGCAATAACAGGTCTGT
GATGCCCTTAGATGTACGGGGCTGCACGCGTGCTACACTGACGGCGTCAGCGTGCGTTCGAGCCCGGCCTGGCAAGGTCG

GGAAATCGGTTGAAACGTTCTCGTGACTGGGACAGGGAATTGCAATTATTTCCCTCGAACGAGGAATTCCCAGTAAGCGC
GAGTCATCAGCTTGCGTTGACTACGTCCCTGCCCTTTGTACACACCGCCCGTCGCTACTACCGATTGGATGATTTAGTGA
GGCCTCTGGACCGATGCCGCAGGTATCCGGCCGCGCTGGTTGGACCGCGGCGTCGGGAAACTGGCCAAACCAAACCATCT
AGAGGAAGTAAAAGTCGTAACAAGGTTTCCGTAGGTGAACCTGCGGAAGGATCATTATCAGAAGGGAAGAAGATGACGAC
AAGCGCCTGCACCGGCGCGCCGCTTAGCGCGACAGCGCGGAGCCTGCTGCTCCGTGGTGGTCGCGCACGTCCAGGTCTGC
CGGCCTGCGGCCGGTTGGGCCGCGGCCGTCGACATTGTGCACCGGTACCTGCTCCGCTTGGGCTCCTGTGGCGGCAGTGT
GGATCTGGCTGTCGCCGTGGCCGCCGGTGGCAGTCTCGCTGCTGGGCGAGACCAGGTTCAAAGAACGGGAGTCGGCGGCG
GCGGCGGTGGCGTCGTTGTCGGCCGCCGCCGTCGACGGCTCGTCGCCGGTTGTAAGGCGGAGCCGACCGACCGACGACAG
TGTCGGTCGGCTCCGCAACCAAGAAAAGAAGGCTTAGGGAAAGTTCGAAAAAGTGCTAGCTTTGAAAAAGAAAAGGAACG
ACATTCCGAACGGCGGATCACTTGGCTCGTAGGTCGTTGAAGAACGACGTGACACTCGAGAATTGATGTGAATTGCAGAC
ACACTGAACTTGAATACTTTGAACGCACATTGC
>AB699092 - Trichuris trichiura gene for 18S ribosomal RNA, partial sequence, isolate: Tt1-Macaque
CAGCAGTTGTTGTTCGCTAGAACTGATGTCCACTTGGATAACTATGGAAATGCTAGAGCTAATACATGCCTCGAAGCTCA
GTCGCGCTCTGCGCGTCGGAGCGCGTTTATTTGAACAAAACCAATCGGACGCAGGCTAGCTATTGGTCTGAGTCCGCGAA
ATGTTGGTGAATCGGAATAACTATGCTGATCGCACGGTCCAGTACCGGCGACGATGCTTTGAAACGACTTGCTCATCAAC
TTTCGATGGTACGCTACGTGCTTACCATGGTGACAACGGTTAACGGAGAATCAGGGTTCGGCTCCGGAGAGGGAGCCTGA
GAAACGGCTACCACATCCAAGGAAGGCAGCAGGCACGCAAATTACCCACTCCCAGATCGGGGAGGTAGTGACGAAAAATA
ACGGAACGTTTCTCCATGAGACGCGTTACCGGAACGATCGAGCCGTACATAAGTTCGGCTAAATCTATTGGAGGGCAAGT
CTGGTGCCAGCAGCCGCGGTAATTCCAGCTCCAATAGCGTATATTAAAGTTGCTGCGGTTAAACCGCTCGTAGTTGGATT
GCGGATGTCGACGACGGTCGTCCTAAGCAGGAGTCGTTCCGTCGCTCGTCACCTGTTCGATCAAGATTGCCCTGGATGCT
CTTCAGTGAGTGTCCTTGGCGACTTGAAAGTTTACTTTGAGAAAATGAGAGCGCTCAAGGCAAGCCGTAGTGCTTGAACC
GTGGTGCATGGAATAATGAAAGATGGCCTCAGTGCTATTTTGTTGGTTTACGGCTACGAGGCAATGATTAAAAGAGACTG
ACGGGGACATTCGTATTGCTGCGTTAGAGGTGAAATTCTTGGATCGCAGCAAGACGACCAATTGCGAAAGCATTTGTCAA
GAATGTTTTCATTAATCAAGAACGAAAGTTAGAGGTTCGAAGGCGATCAGATACCGCCCTAGTTGTGACCGTAAACGATG
CCAACCAGCGATTCGGCGATGTTCTTTTTGTGACTCGCTGAGCAGCTTCCGGGAAACCAAAGTGTTTCGGTTCCGGGGGA
AGTATGGTTGCAAAGCTGAAACTTAAAGGAATTGACGGAAGGGCACCACCAGGAGTGGAGCATGCGGCTTAATTTGACTC
AACACGGGAAAACTCACCCGTCCCGAACACTGTGAGGATTGACAGATCAAGAGCTCTTTCTTGATTCAGTGGGTAGTGGT
GCATGGCCGTTCTTAGTTGGTGGAGCGATTTGTCTGGCTAATTCCGATAACGAACGAGACTCTGGCCTACTAACTAGCGG
CGGTATTCACGTCTCCCGCGCGACGCCGCGCTGCGCTTCGGCTGAGCACAGTCGTCGCCGTTTGCGCGAGCGCTCCGCGG
AGCACCTGTAGTGCCGGCAGCCGCTTCTTAGAGGGACCAGCGACACTTTCGCAAGCCGCACGAGAAAGAGCAATAACAGG
TCTGTGATGCCCTTAGATGTACGGGGCTGCACGCGTGCTACACTGACGGCGTCAGCGTGCGTTCAAGCCCGGCCTGGCAA
GGCCAGGAAATCGGTTGAAACGTTCTCGTGACTGGGACAGGGAATTGCAATTATTTCCCTCGAACGAGGAATTCCCAGTA
AGCGCGAGTCATCAGCTTGCGTTGACTACGTCCCTGCCCTTTGTACACACCGCCCGTCGCTACTACCGATTGGATGACTT
AGTGAGGCCTTTGGACCGATGCCGCCGGCGTTCGGCCGTCAGGTTGAACCACGGCGTTGGAAAACTGGCCGAACCAAGCC
ATCTAGAGGAAGTAAAAGT
>EF514913 - Angiostrongylus costaricensis 18S ribosomal RNA gene, partial sequence
AGGAGTTCAGCTTCAAGTGAAACTGCGAACGGCTCATTAGAGCAGATGTGATTTATTCGGAAAATCCTATTGGATAACTG
CGGTAATTCTGGAGCTAATACATGCGTATAAACCCTGACTTTCGAAAGGGTGCAATTATTAGAGCAAATCAATCATTTTC
GGATGCAGTTTGCTGACTCTGAATAACGCAGCATATCGGCGGCTTGTTCGCTGATAATCCGAAAAAGTGTCTGCCCTATC
AACCTGATGGTAGTCTATTAGTCTACCATGGTTATTACGGGTAACGGAGAATAAGGGTTCGACTCCGGAGAGGGAGCCTT
AGAAACGGCTACCACATCCAAGGAAGGCAGCAGGCGCGAAACTTATCCAATCTTGAATAGATGAGATAGTGACTAAAAAT
AAAAAGACCATTCCTATGGAACGGTTATTTCAATGAGTTGATCATAAACCTTTTTTCGAGTATCAAGTGGAGGGCAAGTC
TGGTGCCAGCAGCCGCGGTAATTCCAGCTCCACTAGTGTAAATCGTCATTGCTGCGGTTAAAAAGCTCGTAGTTGGATCT
GAGTTACATGCAATGATTCGCCTTTGGCGTTAATCATTGTTGTGACTATTTGCTGGTTTTCTATTGAAATCTCGATTTCT
TTAGTGGCTAGCGAGTTTACTTTGAATAAATTAGAGTGCTCAGAACAAGCGTTTGCTTGAATGGTCGATCATGGAATAAT
AAAAGAGGACTTCGGTTCTATTTATTGGTTCAGGAACGGAAGTAATGATTAAGAGGGACAATTCGGGGGCATTCGTATCC
CTGCGCGAGAGGTGAAATTCGTGGACCGCAGGGGGACGCCCTAAAGCGAAAGCATTTGCCAAGAATGTCTTCATTAATCA
AGAACGAAAGTCAGAGGTTCGAAGGCGATTAGATACCGCCCTAGTTCTGACCGTAAACTATGCCATCTAGCGATCCGATG
GGGTATTGTTGCCTTGTCGAGGAGCTTCCCGGAAACGAAAGTCTTTCGGTTCCTGGGGTAGTATGGTTGCAAAGCTGAAA
CTTAAAGAAATTGACGGAATGGCACCACCAGGAGTGGAGCCTGCGGCTTAATTTGACTCAACACGGGAAAACTCACCCGG
CCCGGACACCGTAAGGATTGACAGATTGAAAGCTCTTTCTCGATTTGGTGGTTGGTGGTGCATGGCCGTTCTTAGTTGGT
GGAGCGATTTGTCTGGTTTATTCCGATAACGAGCGAGACTCTAGCCTGCTAAATAGTGACTAGATTACTGTGTCTAGTCT
ACTTCTTAGAGGGATAAGCGGTGTTTAGCCGCACGAGATTGAGCGATAACAGGTCTGTGATGCCCTTAGATGTCCGGGGC
TGCACGCGCGCTACAATGGAAGAATCAACTGGCCTATCCATTGCCGAAAGGTATTGGTAAACCGTTGAAACTCTTCCGTG
ACCGGGATAGGGAATTGTAATTATTTCCCTTGAACGAGGAATTCCTAGTAAGTGTGAGTCATCAGCTCACGCTGATTACG
TCCCTGCCATTTGTACACACCGCCCGTCGCTGTCCGGGACTGAGCTGTCTCGAGAGGACTGCGGACTACTGTATTGAGGC
CTTCGGGTCGCGATATAGCGGGAAACAGTTCAATCGCAATGGCTTGAACCGGGTAAAAGTCGTAACAAGGTATCTG
>KR063346 - Angiostrongylus cantonensis 18S ribosomal RNA gene, partial sequence
TATTAGAGCAAATCAATCATTTTCGGATGTAGTTTGCTGACTCTGAATAACGCAGCATATCGGCGGCTTGTTCGCCGATA
ATCCGAAAAAGTGTCTGCCCTATCAACCTGATGGTAGTCTATTAGTCTACCATGGTTATTACGGGTAACGGAGAATAAGG
GTTCGACTCCGGAGAGGGAGCCTTAGAAACGGCTACCACATCCAAGGAAGGCAGCAGGCGCGAAACTTATCCAATCTTGA
ATAGATGAGATAGTGACTAAAAATAAAAAGACCATTCCTATGGAACGGTTATTTCAATGAGTTGATCATAAACCTTTTTT
CGAGTATCCAGTGGAGGGCAAGTCTGGTGCCAGCAGCCGCGGTAATTCCAGCTCCACTAGTGTAAATCGTCATTGCTGCG
GTTAAAAAGCTCGTAGTTGGATCTGAGTTGCATGCAATGATTCGCCTTTGGCGTTAATCATTGTTGTGACTATTTGCTGG
TTTTCTATTGAAATTTCGATTTCTTTAGTGGCTAGCGAGTTTACTTTGAATAAATTAGAGTGCTCAGAACAAGCGTTTGC
TTGAATGGTCGATCATGGAATAATAAAAGAGGACTTCGGTTCTATTTATTGGTTCAGGAACTGAAGTAATGATTAAGAGG
GACAATTCGGGGGCATTCGTATCCCTGCGCGAGAGGTGAAATTCGTGGACCGCAGGGGGACGCCCTAAAGCGAAAGCATT
TGCCAAGAATGTCTTCATTAATCAAGAACGAAAGTCAGAGGTTCGAAAGGCGATTAGATACCGCCCTAGTTCTGACCGTA
AACTATGCCATCTAGCGATCCGATGGGGTATTGTTGCCTTGTCGAGGAGC
>DQ464371 - Ancylostoma ceylanicum 18S ribosomal RNA gene, partial sequence
TCGAAAGGGTGCAATTATTAGAGCAAATCAATCACCTTCGGGTGTAGTTTGCTGACTCTAAATAACGCTGCATATCGGCG
GCTTGTCCGCCGATATTCCGAAAAAGTGTCTGCCCTATCAACCTGATGGTAGTCTATTAGTCTACCATGGTTATTACGGG
TAACGGAGAATAAGGGTTCGACTCCGGAGAGGGAGCCTTAGAAACGGCTACCACATCCAAGGAAGGCAGCAGGCGCGTAA

CTTATCCACTCTTGAAGAGATGAGATAGTGACTAAAAATAAAAAGACCATTCCTATGGAACGGTCATTTCAATGAGTTGA
TCATAAACCTCTTTTCGAGGATCAAGTGGAGGGCAAGTCTGGTGCCAGCAGCCGCGGTAATTCCAGCTCCACTAGTGTAA
ATCGTCATTGCTGCGGTTAAAAAGCTCGTAGTTGGATCTGAGTCGCATGCAGTGGTTCGCCTTTGGCGTTAATCGCTGTT
GCGGCTATTTGCTGGTTTTCTACTGAAGTTTCGGCTTCTTTAGTGGCTAGCGAGTTTACTTTGAATAAATTAG
>DQ503458 - Thelazia lacrymalis 18S ribosomal RNA gene, partial sequence
AGCCATGCATGTCTAAGTTCAAATAACCTAAAATGGTGAAACCGCGAACGGCTCATTATAACAGCTATAATGTACTTGAT
GTTGATTTATCCAACGTGGATAACTGTGGCAATTCTAGAGCTAATACATGCACCAAAGCTCAAACTTACGGATGAGCGCA
TCTATTAGAATGAAAAAACCAATGGGGTTTATGCAAATAAGCTCGCAAAAAAAAAATTGGTGAATCTGAATAGCTATAGC
TGATCGCATGGTCTTGTACCGGCGACGTATCTCACAAGTGTCTGCCTTATCAACTTTCGATGGTAGTTTATGTGCCTACC
ATGGTTGTAACGGGTAACGGAGAATAAGGGTTCGACTCCGGAGAGGGAGCCTGAGAAACGGCTACCACATCCAAGGAAGG
CAGCAGGCGCGCAAATTACCCACTCTCGGCATGAGGAGGTAGTGACGAAAAATAACGAGACCGTTCTCTTTGAGGCCGGT
TATCGGAATGGGTACAATTTAAACCTGTTAACGAGGATCTATGAGAGGGCAAGTCTGGTGCCAGCAGCCGCGGTAATTCC
AGCTCTCAAAGTGTATATCGTCATTGCTGCGGTTAAAAAGCTCGTAGTTGGATCTGCGTCTTAGGTGTTGGTCTATTCAA
ATAGAATATGAACTAATAACCTAGACTAATTAATAGCTAGTTTTATTATATGTTACCTTAACTGGTTGCATATAATGGCC
GGCATGTTTACCTTGAAAAAATTAGAGTGCTTAAAGCGGGCTTAAAATGCTTGAATATTTGTGCATGGAATAATGAAATA
GGATCTTGATTGTTGTATGCCTATTTTATTGGTTTTGTAACATTTTGCTACAATTGAGATAATGGTTAAGAGGGACGGAC
GGGGGCATTCGTATCGCTGCGTGAGAGGTGAAATTCTTGGACCGTAGCGAGACGCCCAACTGCGAAAGCATTTGCCAAGA
ATGTCTTCATTAATCAAGAACGAAAGTCAGAGGTTCGAAGGCGATCAGATACCGCCCTAGTTCTGACCGTAAACGATACC
AACTAGCGTTCCGTTGGCGGTAAATACGCCTTGACGGGCAGCTTCCCGGAAACGAAAGTTTTTCGGTTCCGGGGGAAGTA
TGGTTGCAAAGCTGAAACTTAAAGAAATTGACGGAAGGGCACCACCAGGAGTGGAGCCTGCGGCTTAATTTGACTCAACA
CGGGAAAACTCACCTGGCCCGGACACCGTGAGGATTGACAGATTGAAAGCTCTTTCATGATTCGGTGGTTGGTGGTGCAT
GGCCGTTCTTAGTTGGTGGAGTGATTTGTCTGGTTTATTCCGATAACGAGCGAGACTCTAGCCTACTAAATAGTTTATGC
TGGATAAATGTATGAGTAATACGCTAGTCCAGACAACTTCTTAGAGGGACAAGCGGTGTTTAGCCGCATGAAGTTGAGCA
ATAACAGGTCTGTGATGCCCTTAGATGTCCAGGGCTGCACGCGCGCTACACTGGAGGAATCAGCGTGCTGTAACCATTGC
TGAAAAGTTATGGTAACCCCTTGAAAATCCTCCGTGATCGGGATCGGGAATTGCAATTATTTCCCTTGAACGAGGAATTC
CTAGTAAGTGTGAGTCATCAGCTCACGTTGATTACGTCCCTGCCCTTTGTACACACCGCCCGTCGCTGCCCGGGACTGAG
CCGTTTCGAGAAAAGCGGGGACTGCTGTTTCAAAACCTTCAATTATTGATAAGGTGGAGATTCTTTGGTGGAAACCGCTT
TAATCGCAGTGGCTTGAACCGGGCAAAAGTCGTAACAAGGTTTCCGTAGGTGAACCTGCCAGAAGGATCAA
>JN256973 - Toxocara cati isolate Pb1 18S ribosomal RNA gene, partial sequence
AGCCATGCATGTCTAAGTTCAAATGGCCTTTAAAGGTGAAACCGCGAACGGCTCATTATAACAGCTATTATATACTTGAT
CTTGATGTCCTACGTGGATAACTGTGGTAATTCTAGAGCTAATACATGCACCAAAGCTCCGATTTTCTGACGAGCGCATC
TATTAGATTAAAACCAATCGGGTTTCGGCCCGTAAATTGGTGACTCTGAATAACTGTAGCTGATCGCATGGTCCAGAACC
GGCGACGTGTCTATCAAGTGTCTGCCTTATCAACTGTCGATGGTAGTTTATGTGCCTACCATGGTTGTAACGGGTAACGG
AGAATAAGGGTTCGACTCCGGAGAGGGAGCCTGAGAAACGGCTACCACATCCAAGGAAGGCAGCAGGCGCGCAAATTACC
CACTCTCGGCATGAGGAGGTAGTGACGAAAAATAACGAGACCGTTCTCTATGAGGCCGGTTATCGGAATGGGTACAATTT
AAACCCGTTAACGAGGATCTATGAGAGGGCAAGTCTGGTGCCAGCAGCCGCGGTAATTCCAGCTCTCAAAGTGTATATCG
TCATTGCTGCGGTTAAAAAGCTCGTAGTTGGATCTGCGCCTCAGGACTTGGTCCGCCCACTGGGCGAGAACTGGGCTCCT
GGGCTAGTTCTGCTGGTTTTCCCTACGTTGCCTTCATCGGTCGCGTAGGGTGGCTAGCGAGTTTACTTTGAAAAAATTAG
AGTGCTTCACGCGGGCTTATGTCTGAATACTCGTGCATGGAATAATAGAATAGGATCTCGGTTCTATTTTGTTGGTTTTC
TGATCTGAGATAATGGTTAAGAGGGACGGACGGGGGCATTCGTATCGCTGCGTGAGAGGTGAAATTCTTGGACCGTAGCG
AGACGTCCGACTGCGAAAGCATTTGCCAAGAATGTCTTCATTAATCAAGAACGAAAGTCAGAGGTTCGAAGGCGATCAGA
TACCGCCCTAGTTCTGACCGTAAACGATACCAACTAGCGTTCCGTCGGCGGTAAATACGCCTTGACGGGCAGCTTCCCGG
AAACGAAAGTCTTTCGGTTCCGGGGGAAGTATGGTTGCAAAGCTGAAACTTAAAGAAATTGACGGAAGGGCACCACCAGG
AGTGGAGCCTGCGGCTTAATTTGACTCAACACGGGAAAACTCACCTGGCCCGGACACCGTGAGGATTGACAGATTGAGAG
CTCTTTCTTGATTCGGTGGTTGGTGGTGCATGGCCGTTCTTAGTTGGTGGAGTGATTTGTCTGGTTTATTCCGATAACGA
GCGAGACTCTAGCCTACTAAATAGTCATCGGATAAACAAGTGCGGAAGACTTCTTAGAGGGACAAGCGGTGTTCAGCCGC
ATGAAGTTGAGCAATAACAGGTCTGTGATGCCCTTAGATGTCCAGGGCTGCACGCGCGCTACACTGGAGGAATCAGCGTG
CTGTAACCATTGCCGAAAGGTATTGGTAACCCCTTGAAAATCCTCCGTGATCGGGATCGGGAATTGCAATTATTTCCCTT
GAACGAGGAATTCCTAGTAAGTGTGAGTCATCAGCTCACGTTGATTACGTCCCTGCCCTTTGTACACACCGCCCGTCGCT
GCCCGGGACTGAGCCGTTTCGAGAAAAGCGGGGACTGCTGTTTCGAGACCTTCCGAGGTGGAGATTCTTTGGTGGAAACC
GCCTTAATCGCAGTGGCTTGAACCG
>JN256977 - Toxocara canis isolate Al2 18S ribosomal RNA gene, partial sequence
AGCCATGCATGTCTAAGTTTCAATGGCCTATAAAGGTGAAACCGCGAACGGCTCATTACAACAGCTATTATATACTTGAT
CTTGATCTCCTACGTGGATAACTGTGGTAATTCTAGAGCTAATACATGCACCAAAGCTCAGATTTTCTGACGAGCGCATT
TATTAGATTAAAACCAATCGGGTTTCGGCCCGTCAATTGGTGACTCTGAATAACTATTGCTGATCGCATGGTCTCGAACC
GGCGACGTGTCTATCAAGTGTCTGCCTTATCAACTGTCGATGGTAGTTTATGTGCCTACCATGGTTGTAACGGGTAACGG
AGAATAAGGGTTCGACTCCGGAGAGGGAGCCTGAGAAACGGCTACCACATCCAAGGAAGGCAGCAGGCGCGCAAATTACC
CACTCTCGGCATGAGGAGGTAGTGACGAAAAATAACGAGACCGTTCTCTATGAGGCCGGTTATCGGAATGGGTACAATTT
AAACCCGTTAACGAGGATCTATGAGAGGGCAAGTCTGGTGCCAGCAGCCGCGGTAATTCCAGCTCTCAAAGTGTATATCG
TCATTGCTGCGGTTAAAAAGCTCGTAGTTGGATCTGCGCCTCAGGACCTGGTCCGCCCACTGGGCGAGAACTGGGCTCCT
GGGCTAGTTCTGCTGGTTTTCCCTACGTTGCCTTCATCGGTCGCGTAGGGTGGCTAGCGAGTTTACTTTGAAAAAATTAG
AGTGCTTCACGCGGGCTTATGTCTGAATACTCGTGCATGGAATAATAGAATAGGATCTCGGTTCTATTTTGTTGGTTTTC
TGATCTGAGATAATGGTTAAGAGGGACGGACGGGGGCATTCGTATCGCTGCGTGAGAGGTGAAATTCTTGGACCGTAGCG
AGACGTCCGACTGCGAAAGCATTTGCCAAGAATGTCTTCATTAATCAAGAACGAAAGTCAGAGGTTCGAAGGCGATCAGA
TACCGCCCTAGTTCTGACCGTAAACGATACCAACTAGCGTTCCGTCGGCGGTAAATACGCCTTGACGGGCAGCTTCCCGG
AAACGAAAGTCTTTCGGTTCCGGGGGAAGTATGGTTGCAAAGCTGAAACTTAAAGAAATTGACGGAAGGGCACCACCAGG
AGTGGAGCCTGCGGCTTAATTTGACTCAACACGGGAAAACTCACCTGGCCCGGACACCGTGAGGATTGACAGATTGAGAG
CTCTTTCTTGATTCGGTGGTTGGTGGTGCATGGCCGTTCTTAGTTGGTGGAGTGATTTGTCTGGTTTATTCCGATAACGA
GCGAGACTCTAGCCTACTAAATAGTCATCGGATAAACAAGTCCGGAAGACTTCTTAGAGGGACAAGCGGTATTCAGCCGC
ATGAAGTTGAGCAATAACAGGTCTGTGATGCCCTTAGATGTCCAGGGCTGCACGCGCGCTACACTGGAGGAATCAGCGTG
CTGTAACCATTGCCGAAAGGTATTGGTAACCCCTTGAAAATCCTCCGTGATCGGGATCGGGAATTGCAATTATTTCCCTT
GAACGAGGAATTCCTAGTAAGTGTGAGTCATCAGCTCACGTTGATTACGTCCCTGCCCTTTGTACACACCGCCCGTCGCT
GCCCGGGACTGAGCCGTTTCGAGAAAAGCGGGGACTGCTGTTTCGAGACCTTTCGAGGTGGAGATTCTTTGGTGGAAACC

```
GCCTTAATCGCAGTGGCTGAACCG
>KM079654 - Ascaris suum isolate PUG-8.2 18S ribosomal RNA gene, partial sequence
ATGGCCTATAAAGGTGAAACCGCGAACGGCTCATTACAACAGCTATTATATACTTGATCTTGATATCCTACGTGGATAAC
TGTGGTAATTCTAGAGCTAATACATGCACCAAAGCTCCGATTTTCTGACGAGCGCATCTATTAGATTAAAACCAATCGGG
TTTCGGCCCGTCAATTGGTGACTCTGAATAACTATAGCTGATCGCATGGTCTCGAACCGGCGACGTGTCTATCAAGTGTC
TGCCTTATCAACTGTCGATGGTAGTTTATGTGCCTACCATGGTTGTAACGGGTAACGGAGAATAAGGGTTCGACTCCGGA
GAGGGAGCCTGAGAAACGGCTACCACATCCAAGGAAGGCAGCAGGCGCGCAAATTACCCACTCTCGGCATGAGGAGGTAG
TGACGAAAAATAACGAGACCGTTCTCTATGAGGCCGGTTATCGGAATGGGTACAATTTAAACCCGTTAACGAGGATCTAT
GAGAGGGCAAGTCTGGTGCCAGCAGCCGCGGTAATTCCAGCTCTCAAAGTGTAT
>KM079641 - Ascaris lumbricoides isolate HUG-3.1 18S ribosomal RNA gene, partial sequence
ATGGCCTATAAAGGTGAAACCGCGAACGGCTCATTACAACAGCTATTATATACTTGATCTTGATATCCTACGTGGATAAC
TGTGGTAATTCTAGAGCTAATACATGCACCAAAGCTCCGATTTTCTGACGAGCGCATCTATTAGATTAAAACCAATCGGG
TTTCGGCCCGTCAATTGGTGACTCTGAATAACTATAGCTGATCGCATGGTCTCGAACCGGCGACGTGTCTATCAAGTGTC
TGCCTTATCAACTGTCGATGGTAGTTTATGTGCCTACCATGGTTGTAACGGGTAACGGAGAATAAGGGTTCGACTCCGGA
GAGGGAGCCTGAGAAACGGCTACCACATCCAAGGAAGGCAGCAGGCGCGCAAATTACCCACTCTCGGCATGAGGAGGTAG
TGACGAAAAATAACGAGACCGTTCTCTATGAGGCCGGTTATCGGAATGGGTACAATTTAAACCCGTTAACGAGGATCTAT
GAGAGGGCAAGTCTGGTGCCAGCAGCCGCGGTAATTCCAGCTCTCAAAGTGTAT
>Z11590 - S.japonicum small subunit ribosomal RNA gene (partial)
TCTAGAGCTAATACATGCCTTGAATCCCTGACCCGCAAGGGGACRGGTGCATTTATTAGAACAGAACCAACCGGGTGCGG
CTTATGCTGTGCCTGTTACATTCTGTGATGACTCTGGATAACTTTACTGATCGCAGTCGGCCTTGTGTGTCGGCGACGGA
TCTTTCAAATGTCTGCCCTATCAATTTGTTGGTAGGTGATTTGCCTACCATGATGATAACGGGTAACGGGGAATCAGGGT
TCGATTCCGGAGAGGGAGCCTGAGAAATGGCTACCACATCCAAGGACGGCAGCAGGCGCGAAAATTACCCACTCCCAGCA
CGGGGAGGTAGTGACGAAAAATACGGATACGGGACTCAATTGAGGCTCCGTAATTCGAATGAGTACAATTTAAATCCTTT
AACGAGGACCAATTGGAGGGCAAGTCTGGTGCCAGCAGCCGCGGTAACTCCAGCTCCAAAAGCGTATATTAAAGTTGCTG
CAGTTAAAAAGCTCGTAGTTGGATCTGGGTAGTGCGATCGCATGCTGTTGCTTGTTCACGGTCTTGGTTACGATCAGGGC
AGTGGTCAGCTCGGCGTAGTGGTTGTGCAACCTTTCAGCCGTGTCTGTGTTWTATTAAACAGGTGTCGATGGGTTAATGA
GTATTGTATCTTATTGACCTGTTGGCATGCTTTCGGATGCCTTTAAACGGGTGTCGGGGGCGGACGGCATCTTTACTTTG
AACAAATTTGAGTGCTCAAAGCAGGCCTATGCGCCTGAAAATTCTTGCATGGAATAATGAAATAGGACTTCGGTTCTATT
TTGTTGGTTTTCGGATCCGAAGTAATGGTTAAGAGGGACAGACGGGGGCATTTGTATGGCGGTGTTAGAGGTGAAATTCT
GGGATCGCCGCCAGACAAACTACAGCGAAAGCATTTGCCAAGAATGTTTTCATTGATCAGGAGCGAAAGTCAGAGTTTCG
AAGACGATCAGATACCGTCGTAGTTCTGACCATAAACGATGCCAACTGACGATCCGCGTTGGTTCTATAATTGACATCGC
GGGCAGTCCCCGGGAAACCTTTAAGTCTTTGGGCTCCGGGGGGAGTATGGTTGCAAAGCTGAAACTTAAAGGAATTGACG
GAAGGGCACCACCAGGAGTGGAGCCTGCGGTTTAATTCGACTCAACACGGGAAAACTCACCCGGCCCGGACACTGTGAGG
ATTGACAGATTGAAAGCTCTTTCTTGATTCGGTGGTTGGTGGTGCATGGCCGTTCTTAGTTGGTGGAGCGATTTGTCTGG
TTAATTCCGATAACGAACGAGACTTTAACCTACTAAATAGTAGACTGGTCCTCTGTGCTCGTTCAGGGCGCGGCTTCTAC
TGCTTCTTTATGGAGTAGTGTGGTCGTGATCCGGCGGGTGCGGTGCCAGTTTTTACTTCTTAGAGGGACAAGCGGCACAC
TTAAGTCGCACGAAATTGAGCAATAACAGGTCTGTGATGCCCTTAGATGTCCGGGGCCACACGTGCGCTACAATGACGGT
GCCAGCGAGCCTGGAAACCTGGCCCGAAAGGGTTGGGCAAACTGTTTCATCACCGTCGTGACTGGGATCGGGGCTTGCAA
TTATTCCCCGTGAACGAGGAATTCCTGGTAAGTGCAAGTCATAAGCTT
>DQ354363 - Schistosoma intercalatum strain NHM3397 18S ribosomal RNA gene, partial sequence
GCTCATTAAATCAGCTATGGTTCCTTAGATCGTAAATGCTACATGGATAACTGTAGTAATTCTAGAGCTAATACATGCCT
TGAATCCCTGACCCGCAAGGGAACGGGTGCATTTATTAGAACAGAACCAATCGGGCGCGGCTTCGGCTGTGCCTGTTACA
TTCTGTGATGACTCTGGATAACTTTACTGATCGCAGTCGGCCTTGTGTCGGCGACGGATCTTTCAAATGTCTGCCCTATC
AATTTGTTGGTAGGTGATTTGCCTACCATGATGATAACGGGTAACGGGGAATCAGGGTTCGATTCCGGAGAGGGAGCCTG
AGAAATGGCTACCACATCCAAGGACGGCAGCAGGCGCGAAAATTACCCACTCCCGGCACGGGGAGGTAGTGACGAAAAAT
ACGGATACGGGACTCAATTGAGGCTCCGTAATTCGAATGAGTACAATTTAAATCCTTTAACGAGGACCAATTGGAGGGCA
AGTCTGGTGCCAGCAGCCGCGGTAATTCCAGCTCCAAAAGCGTATATTAAAGTTGCTGCAGTTAAAAAGCTCGTAGTTGG
ATCTGGGTCGTGCGGTCGCATGCCGCTGCTTGTTCACGGTTTTGGTTACAATCAGGACGTGTTCAGCTCGGTGTAGTGGC
TGTGCAGCCTTTCAGCCGTGTCTGTGTTAAACGGGTGCTGGTGGGTTGACGAGTTCGTCTTGTTGACCTGTCGGCATGCT
TCCGGATGCCTTTAAACGGGTGTCGGGAGCGGACGGCATCTTTACTTTGAACAAATTTGAGTGCTCAAAGCAGGCCTGTG
CGCCTGAAAATTCTTGCATGGAATAATGAAATAGGACTTCGGTTCTATTTTGTTGGTTTTCGGATCCGAAGTAATGGTTA
AGAGGGACAGACGGGGGCATTTGTATGGCGGTGTTAGAGGTGAAATTCTGGGATCGCCGCCAGACAAACTACAGCGAAAG
CATTTGCCAAGAATGTTTTCATTGATCAGGAGCGAAAGTCAGAGTTTCGAAGACGATCAGATACCGTCGTAGTTCTGACC
ATAAACAATGCCGACTGATGATCCGCGTTGGTTCTATAATTGACATCGCGGGCAGTCCCCGGGAAACCTTTAAGTCTTTG
GGCTCCGGGGGGAGTATGGTTGCAAAGCTGAAACTTAAAGGAATTGACGGAAGGGCACCACCAGGAGTGGAGCCTGCGGT
TTAATTCGACTCAACACGGGAAAACTCACCCGGCCCGGACACTGTGAGGATTGACAGATTGATAGCTCTTTCTTGATTCG
GTGGGTGGTGGTGCATGGCCGTTCTTAGTTGGTGGAGCGATTTGTCTGGTTAATTCCGATAACGAACGAGACTTTAACCT
GCTAAATAGTAGACTGGTCCTCTGTGCTCGTTTAGGGCGCGGCTTCTATTGCTTCTTTATGGAGTAGTGTGGTCGTGATC
CGGCGGGTGCGGTGCCAGTTTTTACTTCTTAGAGGGACAAGCGGCACACTTAAGTCGCACGAAATTGAGCAATAACAGGT
CTGTGATGCCCTTAGATGTCCGGGGCCACACGTGCGCTACAATGACGGTGCCAGCGAGTCTGGGAACCTGGCCCGAAAGG
GTTGGGCAAACTGTTTCATCACCGTCGTGACTGGGATCGGGGCTTGCAATTATTCCCCGTGAACGAGGAATTCCTGGTAA
GTGCAAGTCATAAGCTTGCGCTGATTACGTCCCTGCCCTTTGTACACACCGCCCGTCGCTACTACCGATTGAATGGTTTA
GTGAGGTCGTTGGATTGGTGTCGTTGTAGTGGCGTTGCCGCTCGACTGATGCTGAGAAGATGACCTAATTTGACTATTTA
GAGGAAGTAAAAGTC
>AB731643 - Dipylidium caninum gene for 18S rRNA, partial sequence, sample code: Dcan
CTATGGTTTATTGGATCTTACCCGTTAAATGGATAACTGTAATAACTCTAGAGCTAATACATGCCTCGATGCCCTGACCG
CGCTGACCGCTTGGCGGGTCTCTCGACCTGCCGGCTGGACGGCGGGGATGGGTGCACTTATTAGATCAGAAGCCAACCGA
CTGCGTGCATGCGGCTTTGAGTGGTCGCTGCGCGGGTTGAGGCACTTCTGGTGACTCTGGATAATTGTTACAGATCGCAG
TTGGCCGTGTGTCGGCGACGGATCCTTCAAATGTCTGCCCTATCAACTTTCGATGGTAGGTGACCTGCCTACCATGGTGA
TAACGGGTAACGGGGAATCAGGGTTCGATTCCGGAGAGGGAGCCTGAGAAACGGCTACCACTTCCAAGGGAGGCAGCAGG
CGCGCAAATTACCCACTCCCAGCACGGGGAGGTGGTGACGAAAAATACCGATGCGGGACTCCTTAAAGAGGCTCCGTAAT
CGGAATGAGTGGACTCTAAATCCTTTCACGAGGATCAATTGGAGGGCAAGTCTGGTGCCAGCAGCCGCGGTAACTCCAGC
TCCAATAGCGTATATTAAAGTTGCTGCAGTTAAAAAGCTCGTAGTTGGATCTCGGTTGCATTGTCGCCTCCCGGTACTTG
```

GGCGGCTGGTGTGTGGCCTACGGTGCACTGTAGTGGTGTTGCTGCGCCGGCCCGTCTGTCTGTTCGCCTGTTCGTTGGCT
CGCCTTGTCGCTGATCGCCTGATCGCCCGCCCGTTATGAGGTGCGCGTGACTGCGTGCGGAGTGTCGCCTGGCGGTCTGT
GTTCCGCCATCGTGTGGCCTCTTCGCGTGGGCCATTGTGTGCTTGCGGTGTTGACGGTGTTGACGATTTTGGCGGTGTTG
GCGAGCGGCGCCAGCGGGGGCATTTGGTGGTGGCGGCGCTGTGGCGGTGTTGCCGGGGCCATTAATTAGCACTGTCGCGT
CAAGCTGGGGGGTCGGCGGTGCCACCTTTGAGCCATGTCTGTGGCCTCACGGTCGCAGGTGTGGGCGGGTGTCGGATAGG
GCCCTGCATACGCTGTGGGGCCTGTCGGCCCGTCTGCATGCCTTTGGATGCCCTTCAAAAGGTGTCTGTGGGCGGATGGC
ACGTTTACTTTGAACAAATTTGAGTGCTCAAATCAGGCCGAGGTTGCCTGAAAAGTTTTGCATGGAATAATGGAATAGGA
CTTCGGTTCTATTTCGTTGGTTTTCGGATCCGAAGTAATGATCAATAGAGACAGGCGGGGACGTTTGTATGGCTGCGCTA
GAGGTGAAATTCATGGACCGTAGCCAGACAGACTAAAGCGAAAGCATTCGTCAAGCATGTTTTCATTGGCCATGAGCGAA
AGTCAGAGGCTCGAAGACGATCAGATACCGTCCTAGTTCTGACCATAAACGATGCCAACTGACGATCCGTGGTGGTAGTG
CAGAAGCAACCTTCCCCACGGGCAGTCCCCGGGAAACCCTTAAGTCTTTGGGTTCCGGGGGAAGTATGGTTGCAAAGCTG
AAACTTAAAGGAATTGACGGAAGGGCACCACCAGGAGTGGAGCCTGCGGCTTAATTTGACTCAACACGGGAAAACTCACC
CGGGCCGGACACTATGAGGATTGACAGATTGATGGCTCTTTCTTGATTTGGTGGTTGGTGGTGCATGGCCGTTCTTAGTT
GGTGGAGCGATTTGTCTGGTTAATTCCGATAACGAACGAGACTCCAACCTGCTAATTAGTGCGTTTGTCCACTGCTCCTG
TGTAGGCGGTGCTGGACGAGGCTGCTGTCGTGGGCGTGATGCCTTCTCCATGTCCTCGTCCTCCTCCTCCTCCTCCT
CCTCCTCCTCGCGTCCTTCCTCCGCCTCCTCCGCCTCCTTCGGATGGATGGGTGGACGGATGGATGGATGGATGGATGGA
TGGATGGGTGGGTGGGTGGACGGTTGGGCGGATGGACGTGGGGTTGGTGCTTGCGGCGGTCGGCCGGTCGGGCGTTAGCC
ACCGGGTGCGGCGCGAATGCCTACTTCTTAGAGGGACAAGCGGGAGAAGCCGCACGAAATAGAGCAATAACAGGTCTGTG
ATGCCCTTAGATGTCCGGGGCCGCACGCGCGCTACATTGACGGTGCCAGCGAGTGAGACCTTCTGGCCTGAGAGGGCTGG
GTAAACTGGTCAATCACCGTCACGACAGGGATCGGGGCTTGGAATTGTCCCCCGTGAACGAGGAATTCCTAGTAAGTGCA
AGTCATAAGCTTGCGCTGATTACGTCCCTGCCCTTTGTACACACCGCCCGTCGCTACTACCGATTGAATGGTTTAGTAAG
GTCCTTGGATCGGTGCCATTGTGACGCTCGCCGAGAGGGGATCGTCCGGCAGGCGCTGAGAAGACGACCGAACTTGATCA
TTTAGA
>Z96946 – Gnathostoma binucleatum 18S ribosomal RNA
TCTCCGATTGATTCTGTCGGCGATTATATGCTTGTCTCAAAGGTTAAGCCATGCATGTCTGCGTGCCTACTCTTGAAAAG
TGAAACCGCGAATGGCTCATTACGACAGCTATGATTTACTTGATCTTGATATTCTACTTGGATATCTGTGGTAATTCTAG
AGCTAATACATGCACCAAAGCTCCGACTCTGTGACGAGCGCATTTATTAGAACAAAACCAATCGAGCTTCGGCTCGGTTG
TTGGTGACTCTGAATAACTACGGCTGATCGCACGGTCTCGTACCGGCGACATGTCAATCAAGTATCTGCCTTATCAACTT
TCGATGGTAGGTTATGTGCCTACCATGGTTGTAACGGGTAACGGAGAATAAGGGTTCGACTCCGGAGAGGGAGCCTGAGA
AACGGCTACCACATCCAAGGAAGGCAGCAGGCGCGCAAATTACCCACTCTCGGCATGAGGAGGTAGTGACGAAAAATAAC
GAAACCGATCTCAATGAGGCCGGTTATCGGAATGAGTCACGCTTAAACCCTCTAACGAGGATCTATGAGAGGGCAAGTCT
GGTGCCAGCAGCCGCGGTAATTCCAGCTCTCAAAGTGTATATCGTCATTGTTGCGGTTAAAAAGCTCGTAGTTGGATCTG
CGCCTGAGGACTCGGTCCGTCCACTGGGCGTGAACTGAGCTCCTAGGCAGTTTTGCTGGTTTTCCCTTCGTTGCCTTAAC
TGGTAGCGTCGGGTGGCTAGCGAGCTTACTTTGAAAAAATTAGAGTGCTCCAAACAGGTTTATGCCTGTATAGCCGTGCA
TGGAATAATGGAATAGGATCTCGGTTCTATTTTGTTGGTTTCTGAACCGAGATAATGGTTAAGAGGGACAAGACGGGGGC
ATTCGTATCGCTGCGTGAGAGGTGAAATTCTTGGACCGTAGCGAGACGCCCGACTGCGAAAGCATTTGCCAAGAATGTCT
TCATTAATCAAGAACGAAAGTCAGAGGTTCGAAGGCGATCAGATACCGCCCTAGTTCTGACCGTAAACTATGCCAACTAG
CGATCCGTCGCGGTAATTACGCCTTGACGGGCAGCTTCCCGGAAACGAAAGTCTTCCGGTTCCGGGGGAAGTATGGTTGC
AAAGCTGAAACTTAAAGAAATTGACGGAAGGGCACCACCAGGAGTGGAGCCTGCGGCTTAATTTGACTCAACACGGGAAA
ACTCACCTGGCCCGGACACCGCGAGGATTGACAGATTGATAGCTCTTTCTTGATTCGGTGGATGGTGGTGCATGGCCGTT
CTTAGTTGGTGGAGTGATTTGTCTGGTTTATTCCGATAACGAGCGAGACTCTAGCCTATTAAATAGATATCGGATTGACG
CGTCCGTTATACTTCCTAGAGGAACAAGCGGTGCTCAACCGCACGAAATTGAGCAATAACAGGTCTGTGATGCCCTTGGA
TGTCCAGGGCTGCACGCGCGCTACACTGGAAGAATCAACGTGCCGTAACCATTGCCGAAACGCATTGGTAACCACGTGAA
AATCTTCCGTGATCGGAATCGGGAATTGCAATTGTTTCCCTTGAACGAGGAATTCCCAGTAAGTGTGAGTCATCAGCTCG
CGTTGATTACGTCCCTGCCCTTTGTACACACCGCCCGTCGCTGCCCGGGACTGAGCCGTTTCGAGAAATGCGGGGATCAT
TGTTTCGAAGTCTTCGGGACGGAGACTCTTGGTGAGAACCGCCTTAATCGCAGTGGCTTGAACCGGGTAAAAGTCGTAAC
AAGGTATCTGTAGGTGAACCTGCAGATGGATCATCG

Appendix B.

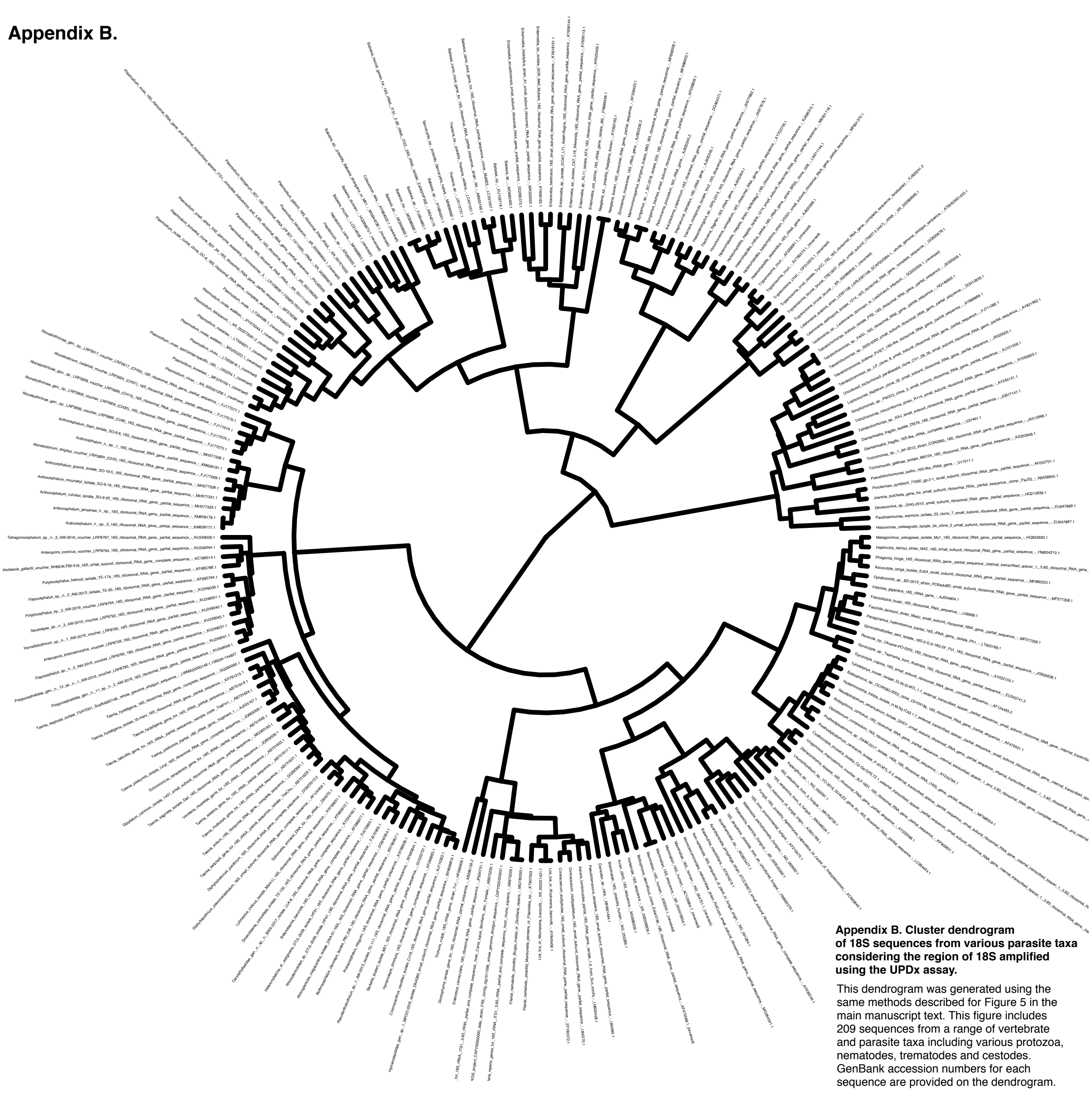

Appendix B. Cluster dendrogram of 18S sequences from various parasite taxa considering the region of 18S amplified using the UPDx assay.

This dendrogram was generated using the same methods described for Figure 5 in the main manuscript text. This figure includes 209 sequences from a range of vertebrate and parasite taxa including various protozoa, nematodes, trematodes and cestodes. GenBank accession numbers for each sequence are provided on the dendrogram.
